# Characterizing acute ischemia via ECG: A study of severity, extent, and spatial variability in myocardial infarction and non-infarction groups

**DOI:** 10.1371/journal.pone.0361220

**Published:** 2026-10-29

**Authors:** Jimena Rodríguez-Carbó, Ana Mincholé, Esther Pueyo

**Affiliations:** 1 Aragon Institute of Engineering Research, IIS Aragón, Universidad de Zaragoza, Zaragoza, Spain; 2 CIBER de Bioingeniería, Biomateriales y Nanomedicina, Instituto de Salud Carlos III, Zaragoza, Spain; Bangabandhu Sheikh Mujib Medical University, BANGLADESH

## Abstract

Ischemic heart disease remains the leading cause of mortality worldwide. Its diagnosis largely relies on detecting dynamic changes in the ST segment of the electrocardiogram (ECG). However, alterations in other ECG waves and intervals have also been reported. One factor that is often overlooked is the presence of a prior myocardial infarction and its influence on the myocardial response to a new ischemic event. This study aimed to quantify the magnitude and temporal dynamics of ECG changes during acute myocardial ischemia and to evaluate how a previous infarction modifies these changes. Twelve-lead ECG recordings obtained before and during acute ischemia induced by percutaneous coronary intervention in one of the three major coronary arteries were analyzed. Dynamic heartbeat averages were computed to extract and track features related to ECG depolarization (QRS width, amplitude, and area) and repolarization (T-wave amplitude and area, ST-segment deviation and area, and T-to-QRS amplitude ratio). The results show that, beyond ST-segment deviation, multiple ECG markers evolve over time during coronary artery occlusion. Repolarization features showed earlier and more pronounced alterations than depolarization features, reaching approximately 37-fold and 23-fold changes at 4 minutes of occlusion compared with baseline, respectively. Males showed faster and more sustained ST increases (up to ~47-fold vs ~30-fold in females); older patients had abrupt early ST peaks (~38-fold at ~2 min after occlusion), whereas younger patients exhibited a more gradual increase. Moreover, patients with a prior infarction showed a stronger association between ECG changes and both the extent and severity of ischemia, reaching maximum correlation coefficients of 0.75 for repolarization markers and 0.57 for depolarization markers. Distinct spatial lead patterns were also identified according to the occluded artery and the presence of a prior infarction. In conclusion, acute ischemia induces dynamic changes across multiple ECG markers beyond the ST segment, where repolarization changes precede and exceed depolarization. These responses are modulated by age and sex; furthermore, prior infarction tightens their correlation with ischemic burden and alters spatial lead patterns.

## Introduction

Cardiovascular diseases (CVD) remain the leading cause of mortality worldwide for both men and women [1]. Among all CVD, ischemic heart disease accounts for the highest number of deaths, with mortality nearly threefold higher than that attributed to the second most common cause, ischemic stroke [2].

Myocardial ischemia constitutes the underlying pathophysiological mechanism of ischemic heart disease and is characterized by a reduction in blood supply to the myocardium as a result of the partial or complete obstruction of one or more coronary arteries. This obstruction most frequently arises from thrombosis or acute pathological changes in coronary atherosclerotic plaques [3].

Acute ischemia induces a series of cellular-level alterations primarily related to hypoxia. Under hypoxic conditions, myocardial energy demands cannot be fulfilled, leading to decreased adenosine triphosphate (ATP) availability [4]. A compensatory increase in anaerobic glycolysis leads to the accumulation of other metabolites, such as lactate, resulting in intracellular acidosis [3]. The reduction in ATP activates ATP-sensitive potassium channels, increasing extracellular potassium (K^+^) concentration. As a consequence, action potentials in ischemic myocardial tissue become shorter in duration and less negative in the resting state [4,5]. When ischemia is prolonged or sustained, irreversible changes may occur, including cardiomyocyte death and the development of myocardial infarction (MI) [4,6].

The electrical manifestations of acute ischemia are highly heterogeneous and depend on multiple factors, including the anatomical location of the occluded coronary artery [7,8], whether the obstruction is proximal or distal [4], and the extent and severity of the ischemic insult [9]. An additional, often underappreciated factor affecting both the temporal evolution of ischemia and its electrocardiographic (ECG) manifestations is the presence of a prior MI [10]. Structural remodeling, scar tissue formation, and altered conduction properties following a previous infarction can significantly modify the myocardial response to a subsequent ischemic event. Clinical investigations have shown that patients experiencing recurrent MI exhibit higher mortality than those with a first MI, even when medical care is sought more quickly [10], underscoring the importance of improved diagnostic tools in this population.

Prompt diagnosis of myocardial ischemia is essential to enable timely intervention aimed at restoring coronary blood flow to the affected area and minimizing cell death. Early reperfusion represents the most effective strategy for preserving viable ischemic myocardium [11]. One of the most commonly employed reperfusion approaches is percutaneous coronary intervention (PCI) [12], which involves balloon inflation within a coronary artery to temporarily induce controlled ischemia, followed by the restoration of blood flow upon deflation. This setting provides an opportunity to investigate the temporal evolution of ischemia-induced ECG changes under well-defined conditions.

In clinical practice, the diagnosis of ischemia in patients with suspected coronary artery disease relies on the evaluation of dynamic changes in the resting 12-lead ECG [12,13]. Acute ischemia most frequently manifests in the ECG as repolarization abnormalities, particularly deviations of the ST-segment [13]. When ST-segment elevation is present in two contiguous leads, the condition is classified as ST-segment elevation myocardial infarction (STEMI) [14]. Nonetheless, other ECG abnormalities may accompany acute ischemia, with or without ST-segment elevation, as observed in non-STEMI presentations [14]. These changes include a reduction in the downward slope of the QRS complex [15] and an increase in the T-wave amplitude [7]. Furthermore, ECG findings such as the absence of acute repolarization changes on resting recordings, together with abnormalities in the Q or R waves, may indicate the presence of a previous MI [13]. Despite their potential diagnostic value, the temporal behavior and clinical significance of ECG markers beyond ST-segment deviation remain insufficiently characterized, particularly in patients with a history of MI.

The main objectives of this study are: 1) to quantify the temporal evolution of ECG markers other than ST-segment deviation during acute myocardial ischemia; 2) to evaluate the influence of prior MI, sex, and age on this evolution; 3) to characterize the relationship between ischemia-induced ECG changes and the extent and severity of coronary occlusion in patients with and without a history of MI; and 4) to describe spatial lead profiles associated with different occluded arteries. We hypothesize that a prior MI occurring in the same myocardial region as the current ischemic event, advanced age, and male sex substantially influence both the temporal and spatial features of ECG alterations, as well as their association with the extent and severity of ischemia.

## Materials and methods

### Study population

This study was conducted using the STAFF III database [16], which comprises ECG recordings of 102 patients undergoing elective PCI. This database is available at https://physionet.org/content/staffiii/1.0.0/. The patients had a mean age of 60.7 ± 11.52 years, and the interventions targeted one of the major coronary arteries: the left circumflex artery (LCx), the right coronary artery (RCA), or the left anterior descending artery (LAD). The occlusion distribution included 21 LCx, 47 RCA, and 34 LAD cases. Although some patients presented with multiple occlusions, only the first balloon inflation of the complete PCI procedure was analyzed. Balloon inflation durations ranged from 1.1 to 9.92 minutes, with an average duration of 4.4 ± 1.32 minutes. Additionally, 34 patients had a history of prior MI, most frequently involving the same myocardial region supplied by the artery targeted during PCI.

Prior MI was identified using ECG-based screening criteria applied to a standard ECG recorded at hospital admission [16]. These screening criteria were based on a subset of the Selvester QRS scoring system [17], which assigns weighted points according to the duration and amplitude of individual QRS deflections across the ECG leads. The screening criteria were: (1) a Q-wave duration ≥30 ms in lead aVF; (2) an R-wave duration ≤10 ms and R-wave amplitude ≤0.1 mV in lead V2; and (3) an R-wave duration ≥40 ms in lead V1. The remaining criteria of the full 32-point Selvester QRS scoring system [18] were considered non-screening criteria. Patients were classified as having no prior MI if none of the three screening criteria was present in any available cardiac cycle and the total score from the non-screening criteria was ≤2 points. Prior MI was defined by the presence of at least one screening criterion in all available cardiac cycles or by a total score ≥4 points from the non-screening criteria, with at least two of these points derived from leads other than V4–V6. Patients who met neither the no-MI nor the MI criteria were classified as borderline for previous MI and excluded from further analysis [19].

Twelve-lead ECG recordings, including both precordial and limb leads, were acquired before the procedure (control recording, CR) and during balloon inflation (PCI recording, PCIR). Standard electrode positioning was used for precordial leads, while limb leads followed the Mason-Likar configuration. All ECG signals were digitized at a sampling frequency of 1000 Hz.

Scintigraphic assessment was performed in a subset of 35 patients using tomographic imaging with 99mTc-sestamibi to quantify the extent and severity of coronary occlusion [20]. Each of these patients underwent two scans: one during arterial occlusion and a control scan acquired 24 hours later. The extent of occlusion was defined as the relative area in which perfusion during occlusion fell below 25% of the corresponding control values and it refers to the physical size of the area affected by the occlusion. The severity was quantified as the percentage reduction in perfusion within the affected area as compared to the left ventricle, calculated as the difference in counts between occlusion and control images relative to the control counts of the left ventricular myocardium in the control study and measures the global perfusion loss of the occlusion on the left ventricle.

### ECG processing

ECG preprocessing consisted of band-pass filtering between 0.5 and 40 Hz, QRS detection using a wavelet-based delineation algorithm [21], and baseline-wander reduction by beat-to-beat cubic spline interpolation, using the fiducial points minus 80 ms as baseline reference points [22]. After preprocessing, ECG signals were segmented into individual heartbeats according to the previously detected QRS fiducial points. Beats were temporally aligned using either the R or S wave, depending on which wave exhibited the largest amplitude during the recording. Cross-correlation analysis was then used to retain only those beats whose similarity to a template beat exceeded a predefined threshold, as described below. The selected beats were subsequently averaged to reduce noise and improve the robustness of ECG feature measurements. Ensemble averaging was applied to CR recordings, whereas exponential averaging was used for PCIR recordings [15,23].

For ensemble averaging in CR recordings, each heartbeat was compared with the median beat computed from the entire recording, and only beats with a correlation coefficient greater than 0.9 were retained. The accepted beats were then averaged within non-overlapping 10-second segments to obtain representative beats for each segment for the CR ECG characterization.

For PCIR, heartbeats were first grouped into 10-second windows. Within each window, a median beat was computed, and beats in that time window with a correlation coefficient greater than 0.9 with the median beat were selected. Exponential averaging was then applied to the selected beats to track dynamic ECG changes during coronary occlusion, as follows:


$$\begin{array}{c} {\overset{―}{x}}_{i}(n)=(1-\alpha )\;{\overset{―}{x}}_{i-1}(n)+\alpha \;x_{i}(n), \end{array}$$ (1)

where α=0.25, *i* denotes the beat index, $x_{i}(n)$, n∈{0,...,N−1} represents the current beat, and ${\overset{―}{x}}_{i}(n)$ denotes the updated averaged beat. For the PCIR ECG characterization, beats were extracted from the final exponentially averaged signal at 10-second intervals.

### ECG markers

The averaged beats obtained from the CR and PCIR recordings were delineated using a wavelet-based algorithm [16] to identify the onsets, peaks, and ends of the ECG waveforms. Based on these delineation points, the following ECG markers were extracted: QRS width ($QRS_{w}$), measured from QRS onset to QRS end; QRS peak-to-peak amplitude ($QRS_{am}$), measured as the difference between the maximum and minimum amplitudes within the QRS complex; QRS area ($QRS_{ar}$), calculated as the integral of the absolute value of the voltage deflections over the duration of the QRS complex; T-wave peak-to-peak amplitude ($T_{am}$), measured as the difference between the maximum and minimum amplitudes within the T wave; T-wave area ($T_{ar}$), calculated as the integral of the absolute value of the voltage deflections over the T wave duration; ST-segment deviation ($ST_{dev}$), measured as the mean value of the segment from the J point to the J point + 50 ms, with the J point calculated as in [24]; ST area ($ST_{ar}$), calculated as the integral of the absolute value of the voltage deflections over the ST segment; and the ratio between the T-wave and QRS amplitudes (*T*/*QRS*).

To evaluate ischemia-related changes during PCIR, we quantified deviations relative to the baseline variability observed during CR using the performance index $R_{\iota }$ [15]. This index was computed for each ECG marker across the 12 leads at discrete time points $t_{j}$ throughout the PCI procedure (Fig 1). $R_{\iota }(t_{j})$ and defined as:


$$\begin{array}{c} R_{\iota }(t_{j})=\frac{\Delta_{\iota }(t_{j})}{\sigma_{\iota }},\;\text{with}\;\Delta_{\iota }(t_{j})=\gamma_{j}t_{j}. \end{array}$$ (2)

**Fig 1:**
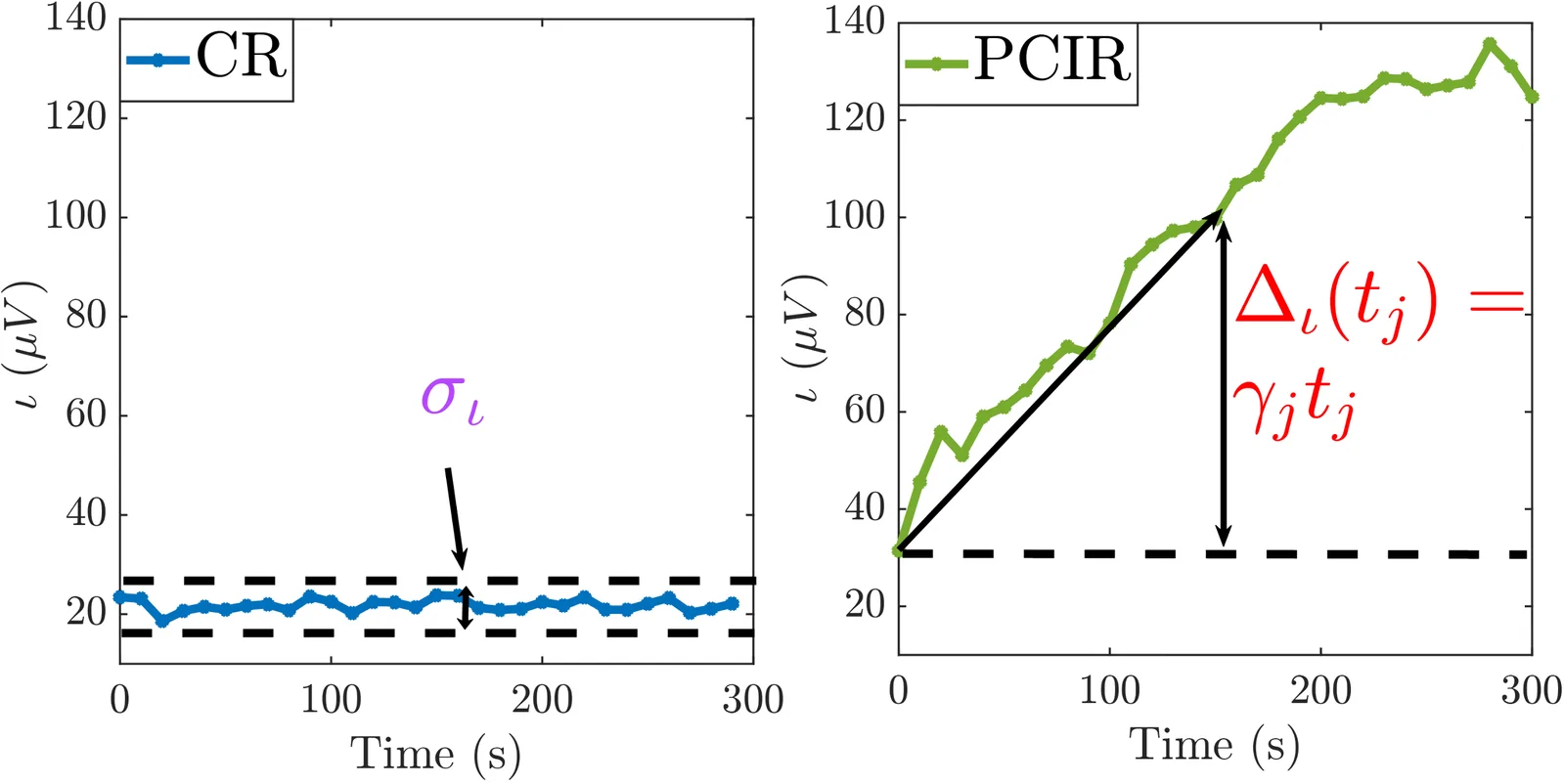
Schematic illustration of the computation of ι from an ECG marker. This computation in shown here for $T_{am}$, in the CR (left) and in the PCIR (right).

Here, $\Delta_{\iota }(t_{j})$ was obtained from the PCIR by fitting a linear model to the temporal evolution of the ECG marker ι from the onset of occlusion (*t* = 0) to time $t=t_{j}$. The time points $t_{j}$ were taken every 10 seconds from the beginning of the occlusion. The parame*t*er $\gamma_{j}$ corresponds to the slope of the fitted linear regression over the interval *t* = 0 to $t=t_{j}$. The normalization factor $\sigma_{\iota }$ represents the standard deviation of the marker ι during the CR.

Clinically, $R_{\iota }(t_{j})$ represents the magnitude of ischemia-induced changes relative to baseline variability, such that larger absolute values indicate deviations that are more likely attributable to ischemia rather than physiological fluctuations.

Fig 2 presents an illustrative example showing the values of the marker $T_{am}$ across the 12 ECG leads during CR (left panel) and PCIR (middle panel), along with the corresponding relative changes quantified by the absolute value of the performance index $R_{\iota }(t_{j})$ ($\left|R_{\iota }(t_{j})\right|$) (right panel) for a patient with RCA occlusion (Fig 2). As can be observed, $T_{am}$ exhibits minimal variability during the CR, whereas pronounced changes occur during the PCIR, particularly in some ECG leads. In this example, leads aVF, V1, III, and aVL show the most prominent ischemia-induced alterations. The relative changes captured by $\left|R_{\iota }\right|$ indicate that changes in $T_{am}$ during ischemia were up to 30 times larger than those observed under baseline conditions.

**Fig 2:**
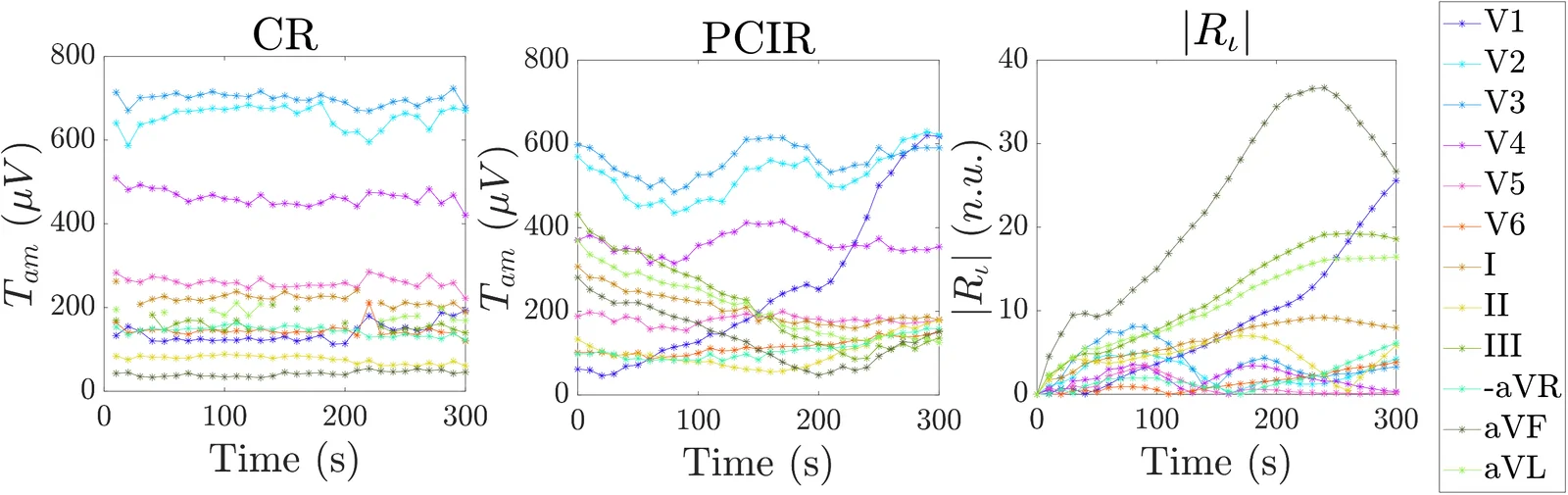
Illustrative example of the marker $T_{am}$ during CR and PCIR and corresponding relative changes. $T_{am}$ values in the CR (left) and in the PCIR (middle), along with the corresponding relative changes quantified by $\left|R_{\iota }\right|$ (right), across all 12 ECG leads for a representative patient with RCA occlusion.

To characterize the overall patient-level response of each ECG marker ι, the $90^{th}$ percentile of $R_{\iota }$ across the 12 leads was computed for each patient at each time point $t_{j}$. These values were then summarized by reporting the median across patients. To account for multiple hypothesis testing, p-values were adjusted using the Benjamini–Hochberg procedure, applied within each family of related comparisons. Because statistical significance is influenced by sample size, effect sizes were additionally reported to quantify the magnitude of the observed differences. Specifically, the effect size *r* was reported for the Wilcoxon rank-sum test, whereas $\eta_{H}^{2}$ was reported for the Kruskal–Wallis test [25]. Following the guidelines in [25], the Wilcoxon rank-sum effect size *r* ranges from −1 to 1 and was interpreted based on its absolute value as small (0.1≤|r|<0.3), medium (0.3≤|r|<0.5), or large (|r|≥0.5). Similarly, the Kruskal–Wallis effect size $\eta_{H}^{2}$ ranges from 0 to 1 and was classified as small ($0.01\leq \eta_{H}^{2}<0.06$), medium ($0.06\leq \eta_{H}^{2}<0.14$), or large ($\eta_{H}^{2}\geq 0.14$), with larger values indicating greater effect magnitude [25].

Finally, to assess the relationship between ischemic burden and ECG changes, Pearson correlation coefficients ρ were calculated between scintigraphically derived measures of extent or severity and $\left|R_{\iota }(t_{j})\right|$. Correlations were evaluated at two clinically relevant time points: $t_{j}=30$ s and $t_{j}=150$ s after the onset of occlusion, representing the early and advanced phases of ischemia during the PCI procedure.

All data analysis was performed using MATLAB R2022b [26]. Statistical significance was set at *p* < 0.05.

## Results

### Temporal dynamics and magnitude of ischemia-induced ECG changes

Table 1 shows the baseline characteristics table that summarizes the study population; it shows the percentage of patients and the median values with the interquartile range. Extent and severity percentages were significantly different between the occluded arteries groups, with LAD occlusion patients having higher values, followed by RCA and the smallest values for LCx occlusion patients. Fig 3 illustrates the temporal evolution of the ECG markers during the first 4 minutes of coronary occlusion across the entire cohort. For each patient, the $90^{th}$ percentile of $\left|R_{\iota }\right|$ across the 12 ECG leads was calculated and subsequently summarized using the group median. As shown in Fig 3, all ECG markers exhibited increases of at least one order of magnitude relative to their control values during PCI.

**Table 1:** Baseline demographic and clinical characteristics of the study population.

Parameters		N	Sex (Female)	Age (years)	Occlusion time (s)	Prior MI (yes)	Extent (%)	Severity (%)
All		102	38.2% (39)	60 (52.7,69.2)	4.9 (3.3,5.0)	33.3% (34)	17.5 (7.8,30.0)	5.5 (1.9,12.3)
Occluded	LAD	34	44.1% (15)	62 (53,70)	3.9 (3,5)	26.5% (9)	47.0 (37.9,48.9)	20.9 (17.6,22.7)
artery	LCx	21	42.9% (9)	66 (52,75)	5 (4.8,5.0)	42.9% (9)	21 (9.8,27.0)	7.1 (3,9.5)
	RCA	47	31.9% (15)	58.5 (65.7,53)	5 (5.0,4)	34.0% (16)	12.1 (17.6−5)	3.4 (5.8-1.1)
Sex	F	39	100% (39)	63 (53,70)	4.8 (3.3,5)	23.1% (9)	17.9 (15.6,30.2)	6.8 (4.9,11.5)
	M	63	0% (0)	60 (51,67)	5 (3.4,5.0)	39.7% (25)	13.6 (7.8,29.6)	4.5 (1.8,12.7)
Prior MI	Yes	34	26.4% (9)	58 (52,67)	4.9 (3.8,5.0)	100% (34)	24.6 (8.4,29.2)	8.1 (1.9,12.3)
	No	68	44.1% (30)	62 (52.5,70)	4.9 (3.1,5)	0% (0)	16.5 (7.2,32.8)	5.3 (1.9,11.9)
Scintigraphy	Yes	35	37.1% (13)	63 (53,73.5)	4.9 (4.8,5)	31.4% (11)	17.5 (7.8,30.0)	5.5 (1.9,12.3)
	No	67	38.8% (26)	59.5 (52.2,67)	4.9 (3,5)	34.3% (23)	- - -	- - -
Age	>60 yrs	49	42.9% (21)	70 (65,75)	5 (3.0,5.0)	24.5% (12)	21 (4.6,33.9)	7.1 (1.1,14.7)
	<60 yrs	51	35.3% (18)	53 (48,56.5)	4.9 (3.9,5)	41.2% (21)	17.4 (8.4,30.0)	4.9 (2.1,12.3)

Baseline characteristics of the study cohort during the PCI procedure. Values are reported as median (IQR). Extent and severity percentages showed statistically significant differences among the occluded artery groups.

**Fig 3:**
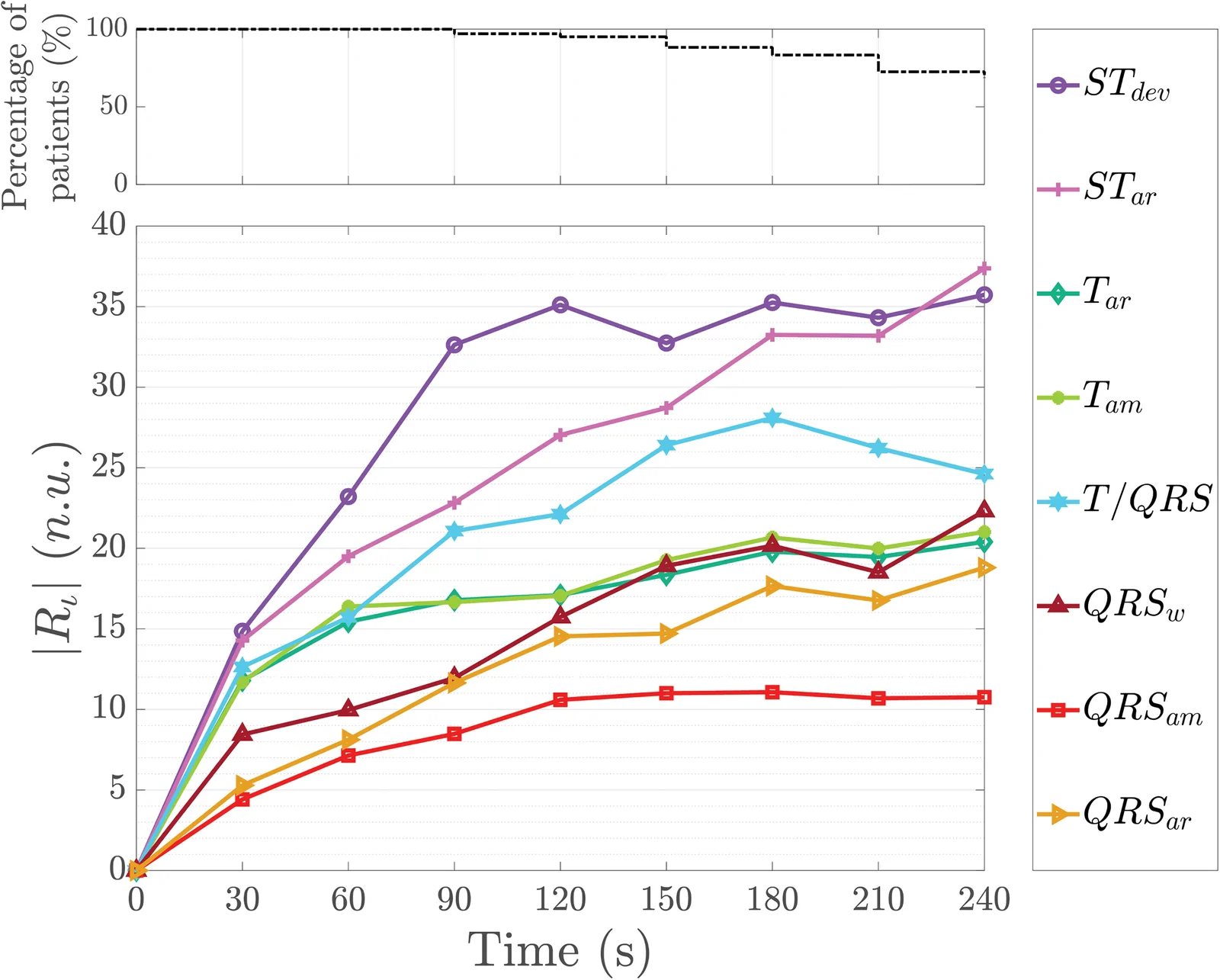
Temporal evolution of the ECG markers during the first 4 minutes of coronary occlusion. Top: Percentage of patients remaining under coronary occlusion over a 4-minute period. Bottom: Median values of $\left|R_{\iota }\right|$ across all patients under occlusion, where each patient’s value is derived from the $90^{th}$ percentile of the 12 analyzed ECG leads. Values are reported every 30 seconds from the onset of occlusion.

Repolarization-related markers ($ST_{dev}$, $ST_{ar}$, and *T*/*QRS*) demonstrated the most rapid response, with pronounced changes occurring within the first 30 seconds of occlusion, showing a change of between 12- and 15-fold compared to baseline conditions. These markers reached peak values of $\left|R_{\iota }\right|$ of 37.38, 35.74, and 24.61, respectively, at 4 minutes, reflecting large fold increases in magnitude relative to baseline. In contrast, depolarization-related QRS markers initially evolved more gradually, with changes of less than 10-fold compared to baseline. However, by the end of the occlusion period, $QRS_{ar}$ and $QRS_{w}$ attained magnitudes comparable to those of $T_{am}$, $T_{ar}$, and *T*/*QRS*.

The upper panel of Fig 3 shows the percentage of patients contributing data at each time point, corresponding to the median $\left|R_{\iota }\right|$ values shown below. S1 Fig (S1 File) presents the same analysis, excluding patients with a prior MI. The behavior of the repolarization and depolarization markers remained unchanged; however, the $ST_{dev}$ and $ST_{ar}$ reached lower values at 4 minutes of occlusion, suggesting that patients with a prior MI have an increased response to ischemia.

### Relationship between ECG changes and sex / age

Having characterized the overall ischemia-induced ECG dynamic, we next examined whether sex and age influenced the temporal patterns or magnitude of ECG responses during coronary occlusion.

#### Sex-related differences.

Fig 4A) shows the temporal evolution of ischemia-induced ECG changes over the first 4 minutes of occlusion, stratified by sex. In both female and male patients, depolarization markers evolved more slowly than repolarization markers, consistent with observations in the full cohort. However, the overall rate of ECG change was higher in males than in females, particularly during the initial 90 seconds of occlusion.

**Fig 4:**
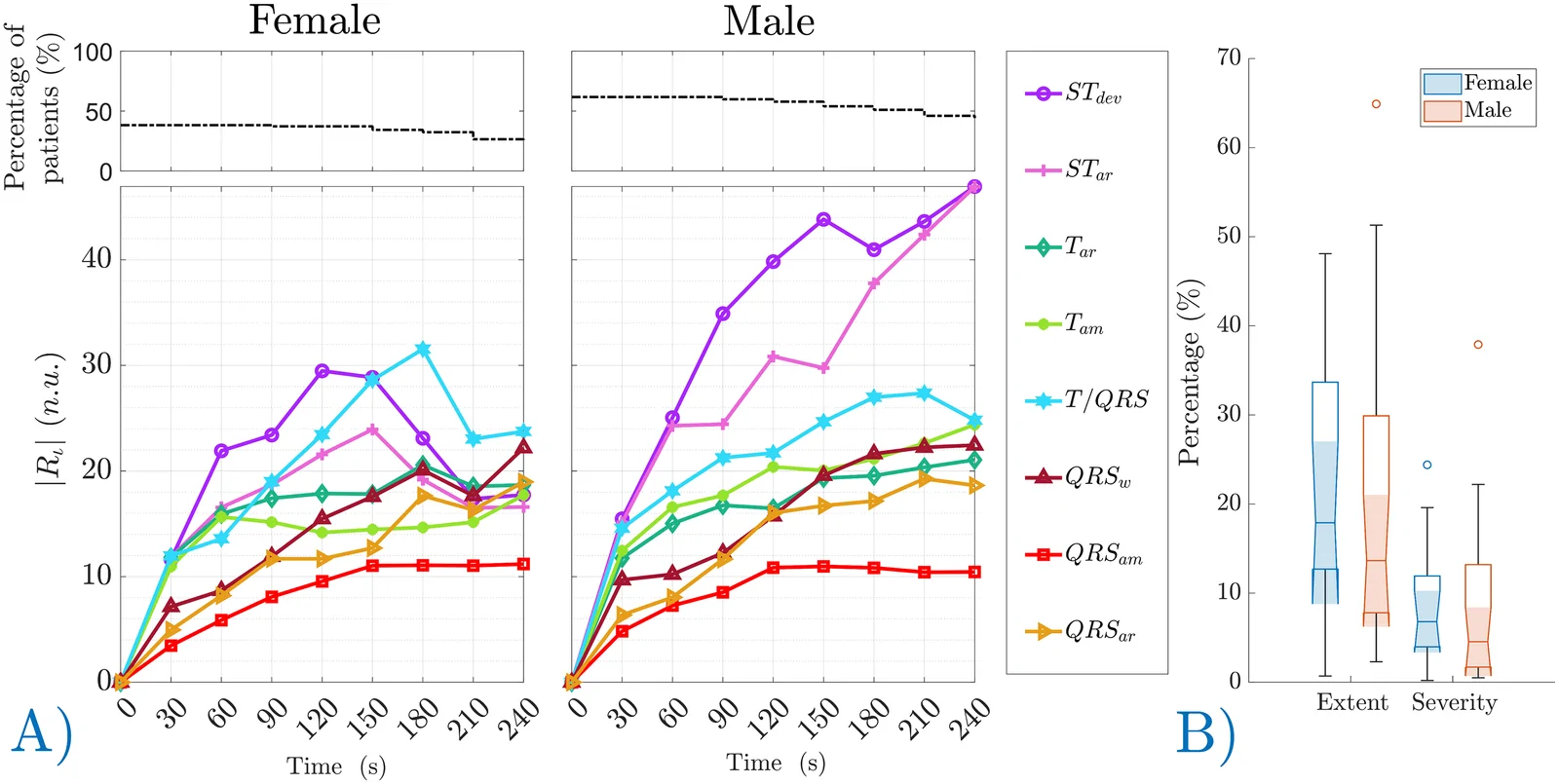
Temporal evolution of the ECG markers during the first 4 minutes of coronary occlusion, stratified by sex. A) Top: Percentage of patients under occlusion over 4 minutes. Bottom: Median $\left|R_{\iota }\right|$ across patients under occlusion, with each patient’s value computed from the $90^{th}$ percentile of the 12 analyzed leads. Values are calculated every 30 seconds from the onset of occlusion, shown separately for female (left) and male (right) patients. B) Box plots showing extent and severity of ischemia for female and male patients.

Sex-specific differences were most evident in repolarization markers. In male patients, $ST_{dev}$ and $ST_{ar}$ increased in an approximately monotonic fashion throughout the 4-minute occlusion and showed the most pronounced responses, reaching peak values up to 47-fold greater than baseline. In contrast, in females, these markers reached peak values (around 30-fold greater than baseline) at approximately 150 seconds and subsequently declined. At 240 seconds, the dominant ischemia-induced response in females was observed in *T*/*QRS*, reaching values approximately 24 times greater than during the control phase.

In contrast to repolarization markers, no marked sex-related differences were observed in either the behavior or magnitude of depolarization markers, suggesting a broadly similar response in females and males.

To exclude the possibility that these differences were driven by variations in ischemic burden, we compared the extent and severity of ischemia between sexes. As shown in Fig 4B), no significant differences were observed, suggesting that the observed ECG differences reflect intrinsic sex-related electrophysiological responses rather than variations in ischemia severity. S2 Fig (S1 File) shows the same analysis after excluding patients with a prior MI, which confirms that the differences in behavior observed previously are not caused by the presence of a prior MI. Although the behavior remains unchanged, ST markers reached lower values, further suggesting that the presence of a prior-MI accentuates the response to ischemic injury.

#### Age-related differences.

To further assess the influence of age on ECG responses, patients were divided into two groups based on the cohort’s median age: younger than 60 years and 60 years or older. Fig 5A) presents the temporal evolution of ECG markers for the younger (left) and older (right) groups. In line with findings in the overall population, repolarization markers in both age groups responded more rapidly than depolarization markers.

**Fig 5:**
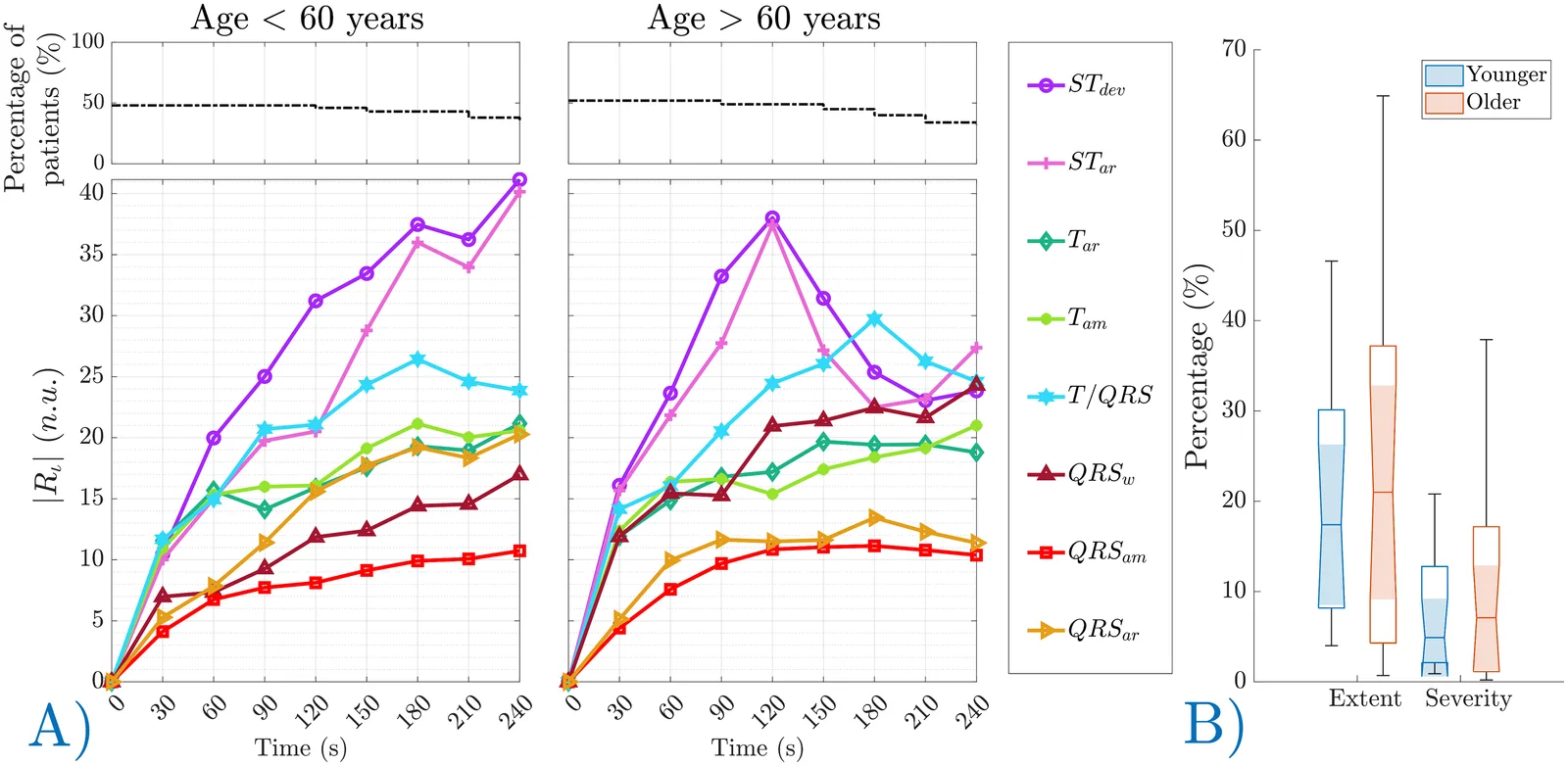
Temporal evolution of the ECG markers during the first 4 minutes of coronary occlusion, stratified by age. A) Top: Percentage of patients under occlusion over 4 minutes. Bottom: Median $\left|R_{\iota }\right|$ across patients under occlusion, with each patient’s value computed from the $90^{th}$ percentile of the 12 analyzed leads. Values are calculated every 30 seconds from the onset of occlusion, shown separately for young (left) and old (right) patients. B) Box plots showing extent and severity of ischemia for young and old patients.

Age-related differences were most evident in the temporal evolution of $ST_{dev}$, $ST_{ar}$, $QRS_{w}$, and $QRS_{ar}$. In older patients, $ST_{dev}$ and $ST_{ar}$ exhibited more abrupt relative changes (16-fold compared to baseline) at the beginning of occlusion, reaching peak values at approximately 2 minutes, followed by a decline. In contrast, younger patients displayed a more gradual increase in these markers, which continued throughout the 4-minute occlusion. Regarding depolarization indices, $QRS_{w}$ increased by approximately 24-fold in older patients compared with 17-fold in younger patients, whereas $QRS_{ar}$ showed more pronounced alterations in younger patients (20 vs 16).

No significant differences in the extent or severity of ischemia were observed between the two age groups (Fig 5B). Moreover, the female-to-male ratio was similar in both groups (18 females/30 males in the younger group and 21 females/31 males in the older group), supporting the interpretation that these observed differences are attributable to age rather than sex or ischemic burden.

S3 Fig (S1 File) presents the same results, excluding patients with a prior MI. The previously reported findings were maintained, except for the modification of ST markers at the end of occlusion in younger patients, during which they reached lower values, again showing an exacerbated response to ischemia when they had a prior-MI.

### Relationship between ECG changes, history of prior MI and ischemia extent / severity

To explore the influence of a prior MI on ischemia-induced ECG alterations, we analyzed the temporal evolution of ECG markers separately for patients with and without a prior MI (Fig 6A). In terms of relative changes, the most apparent difference between the groups was observed for the ST-related markers at 4 minutes of occlusion. In patients with a prior MI, these markers increased progressively, reaching approximately 50-fold their baseline values at 4 minutes. In contrast, patients without a prior MI exhibited their highest values between 120 and 180 seconds, followed by a subsequent decrease to approximately 20- to 30-fold their baseline values at 4 minutes. In patients without a prior MI, repolarization markers consistently dominated the ischemic response, whereas depolarization markers exhibited lower $\left|R_{\iota }\right|$ values, with $QRS_{w}$ being the only depolarization index reaching magnitudes comparable to T-wave markers. In contrast, in patients with a prior MI, the depolarization markers $QRS_{ar}$ and $QRS_{w}$ attained $\left|R_{\iota }\right|$ values similar to those observed for $T_{ar}$ and $T_{am}$ (around 35-fold), indicating a greater relative contribution of depolarization-related changes in patients with prior MI.

**Fig 6:**
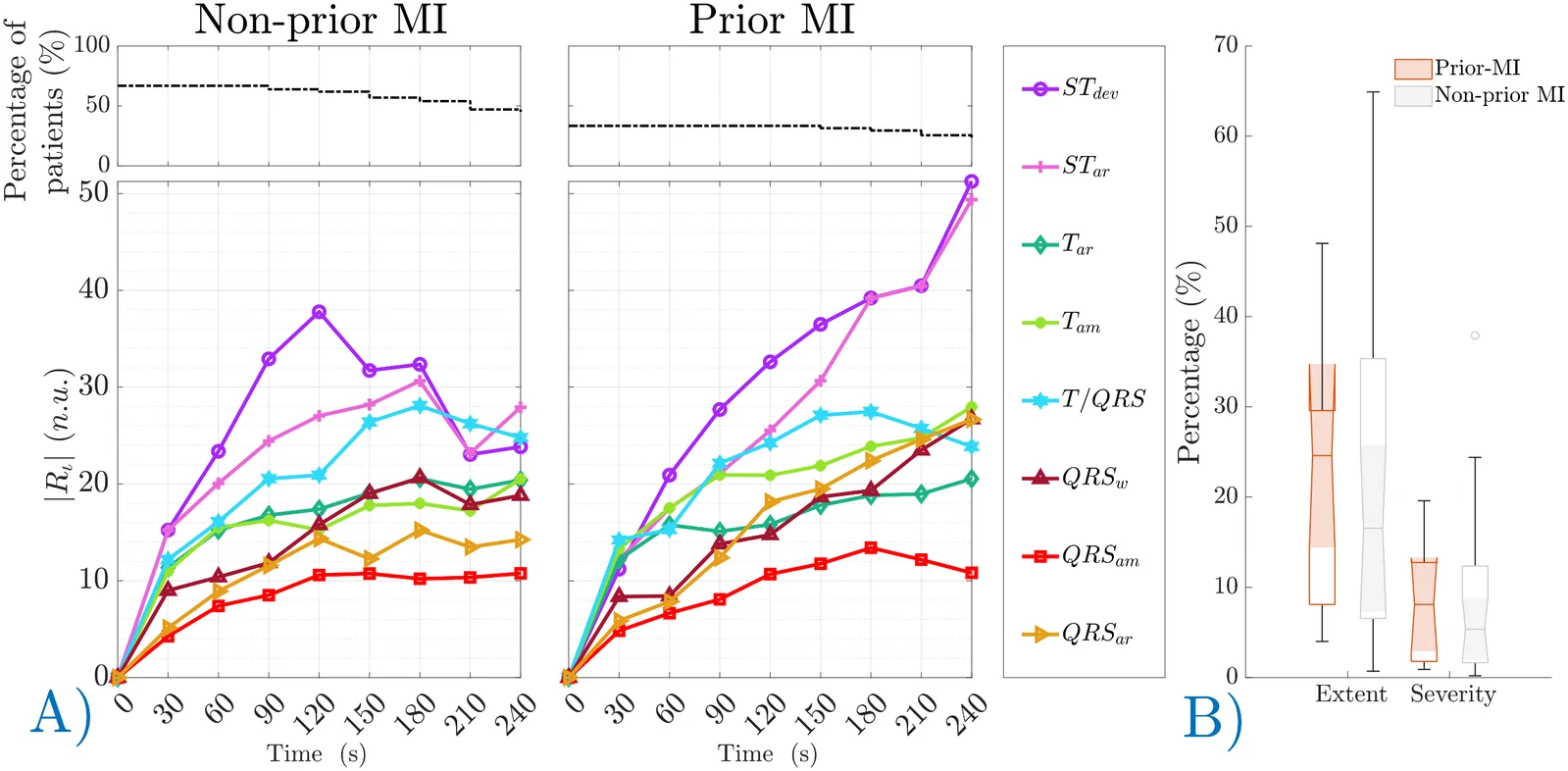
Temporal evolution of the ECG markers during the first 4 minutes of coronary occlusion, stratified by prior MI. A) Top: Percentage of patients under occlusion over 4 minutes. Bottom: Median $\left|R_{\iota }\right|$ across patients under occlusion, with each patient’s value computed from the $90^{th}$ percentile of the 12 analyzed leads. Values are calculated every 30 seconds from the onset of occlusion, shown separately for patients with (left) or without (right) prior MI. B) Box plots showing extent and severity of ischemia for patients with and without prior MI.

At 240 seconds after occlusion onset, several markers, including $QRS_{w}$, $QRS_{ar}$, $T_{am}$, and ST-related indices, continued to increase in patients with a prior MI (Fig 6A), indicating ongoing ischemic progression. However, some indices, such as $QRS_{ar}$ and *T*/*QRS*, appeared to reach a plateau in patients without a prior MI.

Comparisons of the extent and severity of ischemia between patients with and without a prior MI revealed no statistically significant differences; however, slightly higher median values were observed in patients with a prior MI (Fig 6B).

To further investigate the relationship between ECG changes and ischemic burden in the presence or absence of prior MI, we examined the correlation between ischemia extent/severity and changes in ECG markers at 30 and 150 seconds following occlusion onset. Fig 7 shows the scatter plots relating ischemia extent to $\left|R_{\iota }\right|$ for $ST_{dev}$, $T_{am}$, and $QRS_{w}$ in patients with (red squares) and without (gray circles) prior MI. These three indices were selected to illustrate changes in different phases of the cardiac cycle, including the QRS complex, the ST segment, and the T wave. At 30 seconds, repolarization markers demonstrated stronger correlations with ischemia extent and lower variability than depolarization markers (see Fig 7, left). $ST_{dev}$ and $T_{am}$ had ρ values of 0.75 and 0.62 in prior MI patients, with slopes of 1.13 and 0.21, respectively, while $QRS_{w}$ had a non-significant ρ value of −0.36 and a slope of −0.26. By 150 seconds, both depolarization and repolarization markers exhibited increased dependence on the extent of ischemia, accompanied by greater dispersion (Fig 7, right panels). These observations are consistent with the slower temporal response of depolarization markers during coronary occlusion. $ST_{dev}$ and $T_{am}$ had ρ values of 0.54 and 0.38 in prior MI patients, with slopes of 2.93 and 0.96, respectively, while $QRS_{w}$ had a ρ value of 0.57 and a slope of 0.50. For patients without prior MI, the slopes for $ST_{dev}$ and $T_{am}$ were lower than those observed for patients with prior MI (0.74 and 0.56, respectively), whereas the slope for $QRS_{w}$ was comparable between groups (0.43). When the results were analyzed according to ischemia severity, the findings were consistent with those reported for ischemia extent, as shown in S4 Fig (S1 File).

**Fig 7:**
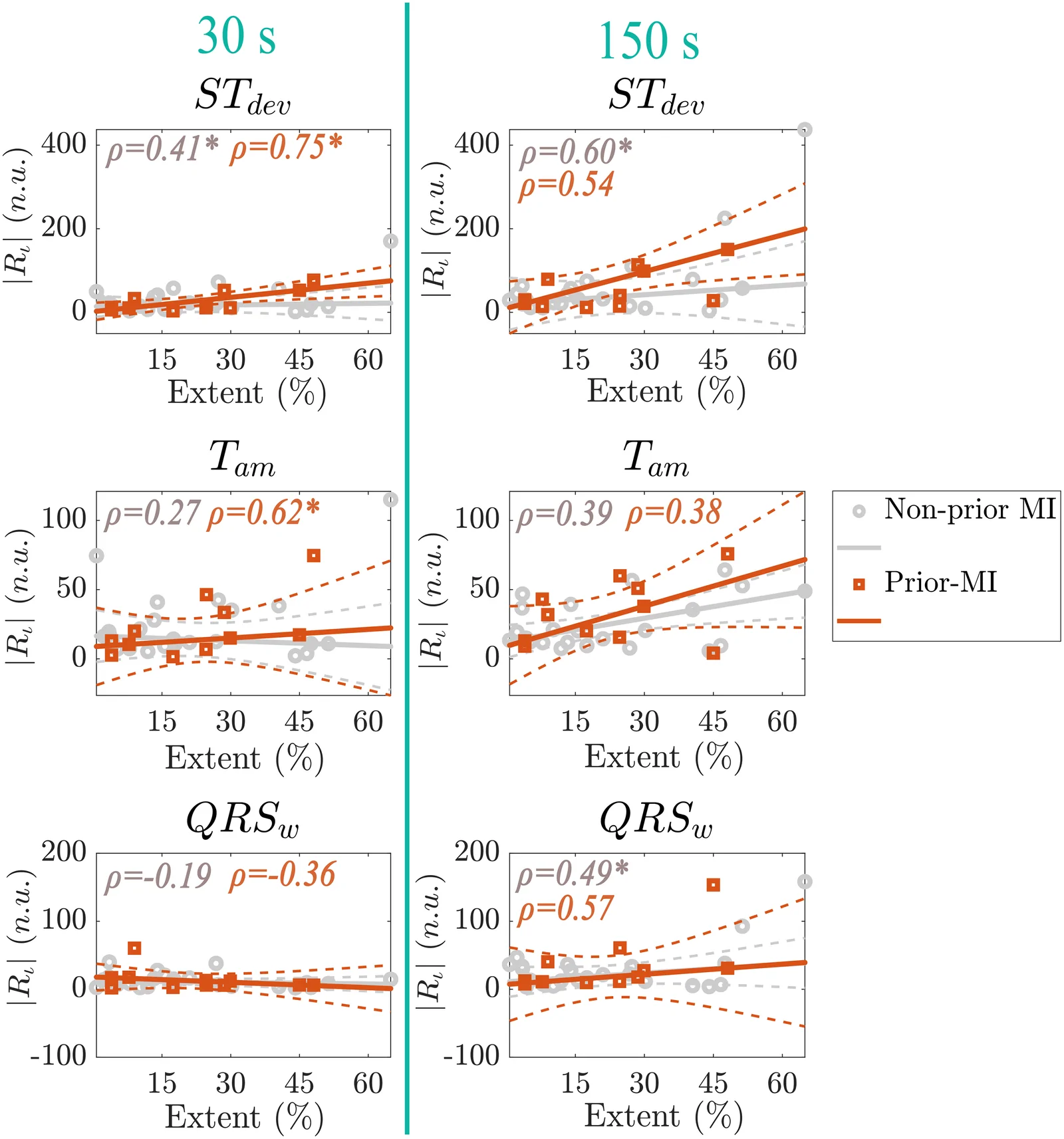
Scatter plots showing the relationship between the extent of ischemia and $\left|R_{\iota }\right|$ for $ST_{dev}$, $T_{am}$, and $QRS_{w}$. The $\left|R_{\iota }\right|$ values are calculated as the $90^{th}$ percentile across the 12 leads for each patient at 30 seconds (left panels) and 150 seconds after the onset of occlusion (right panels). Results are shown for patients with prior MI (red squares) and without prior MI (gray circles). The solid and dotted lines depict the linear regression and its corresponding 95% confidence interval, respectively. Correlation coefficients (ρ) are reported; * denotes statistically significant correlations.

Fig 8 shows averaged heartbeats during ischemia progression for a patient without prior MI and a patient with prior MI, both presenting LAD occlusion and comparable ischemia extent (48.1% and 51.3%, respectively) and ischemia severity (19.6% and 22.2%, respectively). ECG heartbeats at the beginning of the PCI recording are shown in black, whereas progressive gray shading represents advancing occlusion, with the lightest gray shade corresponding to approximately 3.5 minutes from the start of occlusion. As shown in the figure, the patient with prior MI exhibits more pronounced ST-segment changes than the patient without prior MI, whereas QRS-complex changes are more comparable between the two patients. Overall, this example highlights the steeper relationship between ECG repolarization changes and both ischemia extent and severity in patients with prior MI.

**Fig 8:**
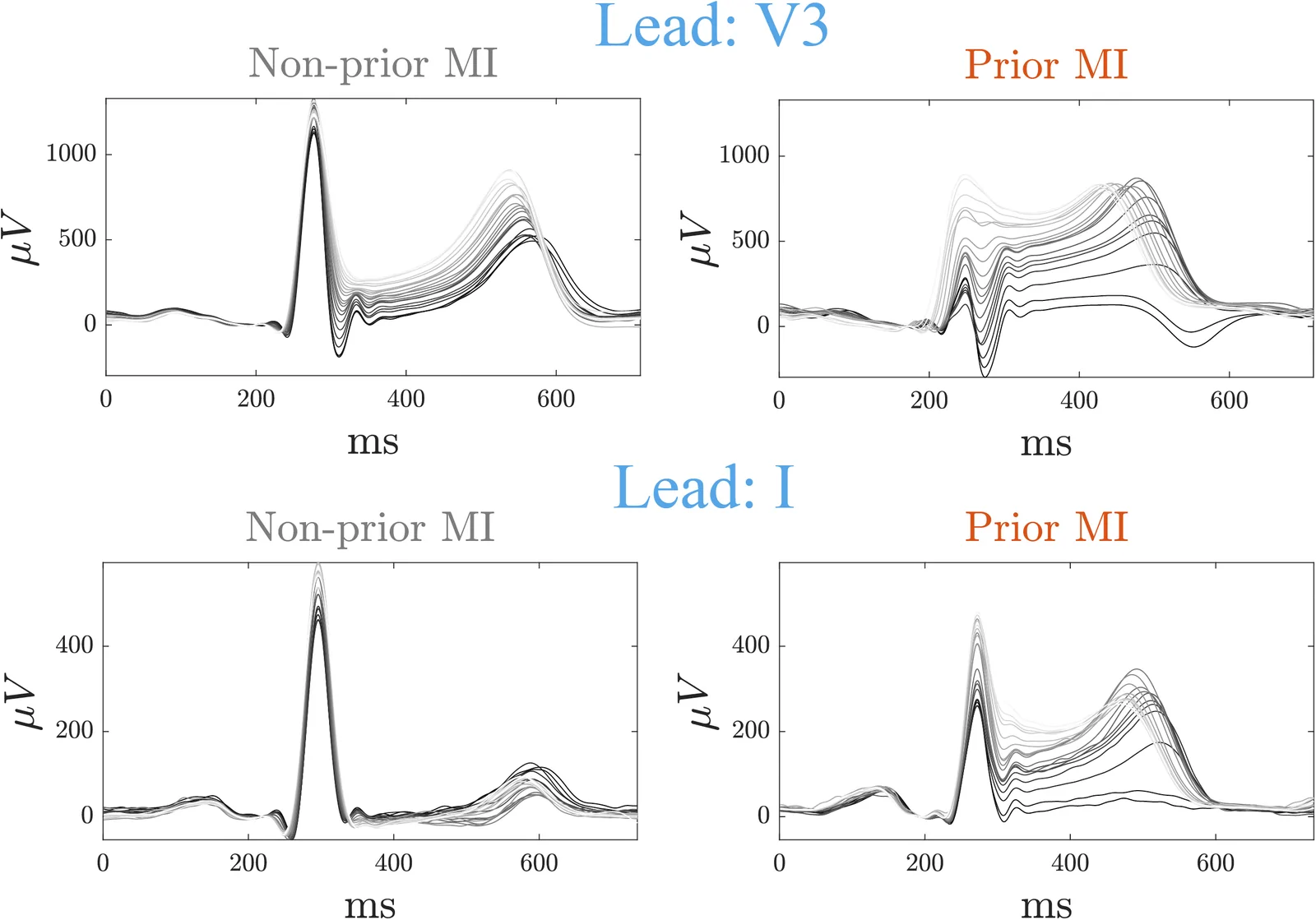
ECG heartbeats during ischemia progression for a patient without prior MI (left) and a patient with prior MI (right), both with LAD occlusion, shown in leads V3 (top) and I (bottom). Black indicates the beginning of the occlusion, and progressively lighter gray shades indicate ischemia progression, with the lightest gray corresponding to approximately 3.5 minutes from the start of the occlusion.

A summary of the entire study population, expressed as the maximum changes in $ST_{dev}$, $T_{am}$, and $QRS_{w}$ during the first four minutes of occlusion and quantified in both absolute $\left|\Delta_{\iota }\right|$ and relative $\left|R_{\iota }\right|$ terms, is presented in Table 2 as median (IQR) values for patients with and without prior MI.

**Table 2:** Median (IQR) values of the patients’ maximum relative $\left|R_{\iota }\right|$ and absolute $\left|\Delta_{\iota }\right|$ changes in $ST_{dev}$, $T_{am}$, and $QRS_{w}$ during the first four minutes of occlusion, stratified by prior MI status.

$\left|R_{\iota }\right|$	Non-prior MI (N = 68)	Prior MI (N = 34)
$ST_{dev}$	39.7915 [19.47, 73.67]	49.83 [15.20, 98.92]
$T_{am}$	23.29 [15.85, 42.71]	30.67 [17.31, 53.56]
$QRS_{w}$	29.63 [14.11, 50.68]	31.11 [11.83, 99.53]
$\left|\Delta_{\iota }\right|$	Non-prior MI	Prior MI
$ST_{dev}$ [μV]	155.79 [72.65, 304.67]	168.19 [95.49, 245.53]
$T_{am}$ [μV]	212.84 [145.83, 299.52]	212.21 [131.15, 361.85]
$QRS_{w}$ [*ms*]	31.54 [21.51, 45.73]	38.94 [15.30, 67.33]

Additional statistical analyses were performed to quantify the relative contribution of prior MI, age, sex, and ischemia extent and severity to ischemia-induced ECG changes using univariable and multivariable regression models. When considering prior MI as the sole independent variable, the proportion of explained variance for each ECG marker at 150 s of occlusion was negligible, with *R*^2^ = 0.003, 0.034, and 0.005 for $ST_{dev}$, $T_{am}$, and $QRS_{w}$, respectively. In contrast, ischemia extent and severity accounted for a substantially larger fraction of the variability, with *R*^2^ values of 0.337/0.512, 0.141/0.175, and 0.255/0.340, respectively. Including prior MI together with ischemic extent or severity in multivariable models resulted in marginal improvements in explained variance, with more accentuated effects for $T_{am}$. Notably, the combination of ischemia extent and severity yielded higher explanatory power (*R*^2^ = 0.711, 0.188, and 0.395), while adding prior MI as an additional covariate produced a slight increase, most evident for $T_{am}$, with *R*^2^ = 0.236. These results indicate that, although prior MI has a limited direct contribution to the overall variability in ECG changes, its presence appears to modestly enhance the strength of the relationship between ECG changes and ischemic extent and severity. Including sex, age, prior MI, and ischemia extent and severity as independent variables in a multivariable model increased *R*^2^ values to 0.727, 0.303, and 0.466 for $ST_{dev}$, $T_{am}$, and $QRS_{w}$, respectively. In this fully adjusted model, ischemia extent and severity were independently associated with $ST_{dev}$, whereas ischemia severity was independently associated with $QRS_{w}$. Age, sex, and prior MI were not independently associated with any of the evaluated ECG markers. For $T_{am}$, the overall multivariable model did not reach statistical significance.

### Relationship between ECG changes and site of occlusion

To examine whether the site of coronary occlusion influences the magnitude and spatial distribution of ischemia-induced ECG alterations, Fig 9 presents selected ECG markers ($ST_{dev}$, $T_{am}$, and $QRS_{w}$) measured 150 seconds after occlusion for patients with LAD, RCA, and LCx artery occlusions. The left panels show the box plots of $R_{\iota }$ for each marker, computed as the $90^{th}$ percentile across the 12 ECG leads at 150 s post-occlusion for each artery group. Repolarization markers ($ST_{dev}$ and $T_{am}$) exhibited the highest median $\left|R_{\iota }\right|$ values in patients with LAD occlusion, followed by RCA, with the lowest values observed in the LCx group. For example, in $ST_{dev}$, patients with LAD occlusion reached median values of 68-fold compared to baseline, 29-fold in patients with RCA occlusion, and 21-fold for patients with LCx occlusion. Statistically significant differences in $\left|R_{\iota }\right|$ were observed across artery groups for $ST_{dev}$, with a medium overall effect size ($\eta_{H}^{2}=0.08$). The significant LAD versus LCx comparison also showed a medium effect size (|*r*| = 0.40). Differences in depolarization markers were less pronounced, although $QRS_{w}$ showed slightly higher median values in the LCx group.

**Fig 9:**
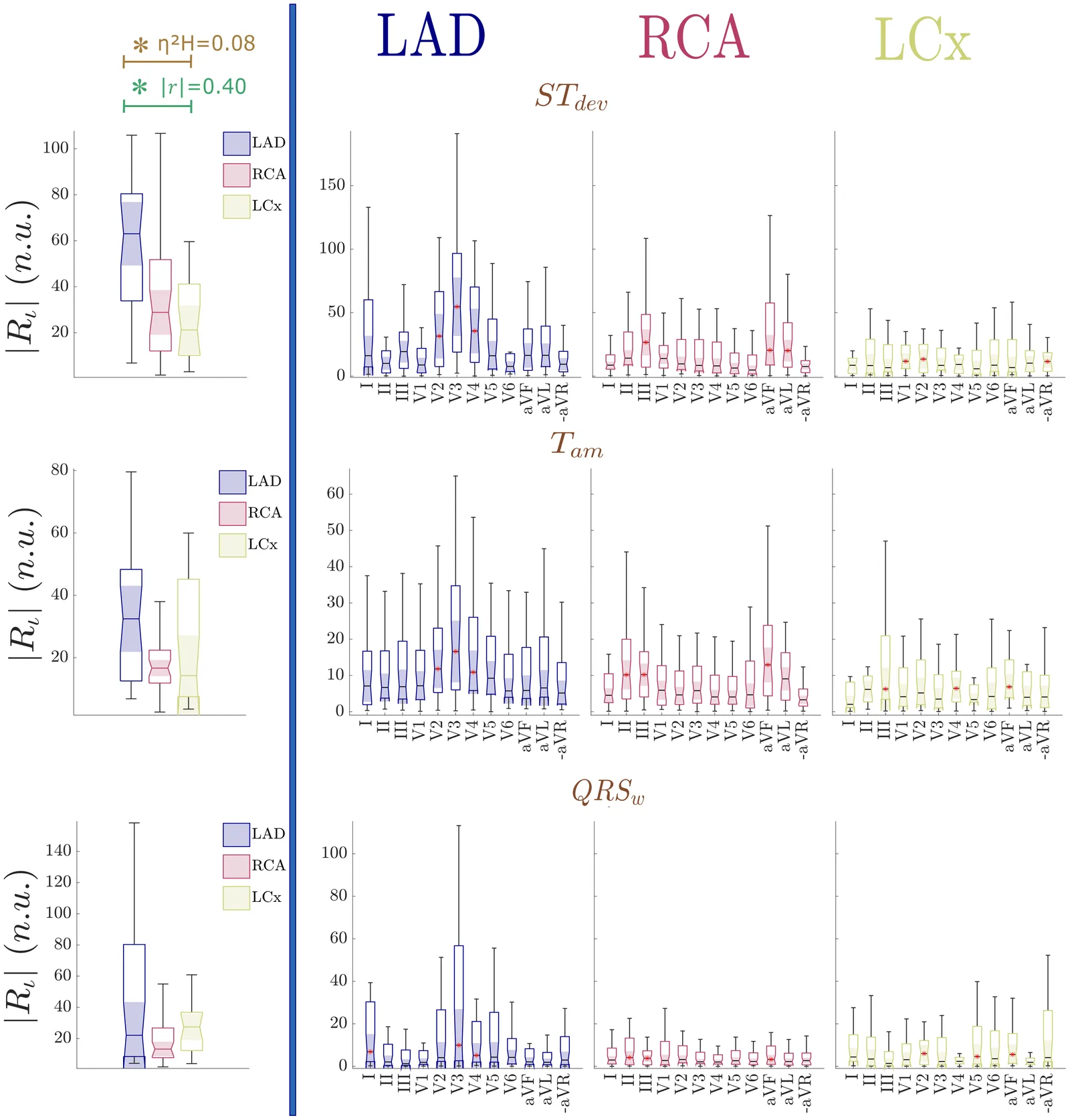
Box plots of $\left|R_{\iota }\right|$ stratified by LAD, RCA, and LCx occlusions. Left: Box plots of $\left|R_{\iota }\right|$ across all patients, with patient-specific values computed from the $90^{th}$ percentile of the 12 analyzed leads at 150 seconds after the onset of occlusion, for $ST_{dev}$, $T_{am}$ and $QRS_{w}$, stratified by LAD, RCA, and LCx occlusions. * denotes statistically significant differences. Effect size |*r*| and $\eta_{H}^{2}$ are reported. Right: Lead-wise $\left|R_{\iota }\right|$ values across all patients at 150 seconds from the onset of occlusion for all 12 analyzed leads, shown for $ST_{dev}$, $T_{am}$ and $QRS_{w}$ for each occluded artery.

The right panels of Fig 9 illustrate the spatial distribution of $\left|R_{\iota }\right|$ across the 12 leads for each artery group. Distinct lead-specific patterns emerged depending on the occluded artery. LAD occlusion produced prominent changes in leads V2–V5, consistent with anterior myocardial ischemia. RCA occlusion was associated with the largest changes in leads II, III, and aVF, whereas LCx occlusion did not yield a clearly distinctive spatial pattern.

Consistent with these ECG findings, patients with LAD occlusion exhibited the highest median ischemia extent and severity, followed by LCx and RCA occlusions (Fig 10). Significant differences were observed across artery groups for both ischemia extent and severity, with medium overall effect sizes ($\eta_{H}^{2}=0.09$ for both variables). Pairwise comparisons involving LAD showed small effect sizes (|*r*| = 0.16–0.17). No significant age differences were observed among the artery groups, suggesting that age was unlikely to account for these differences.

**Fig 10:**
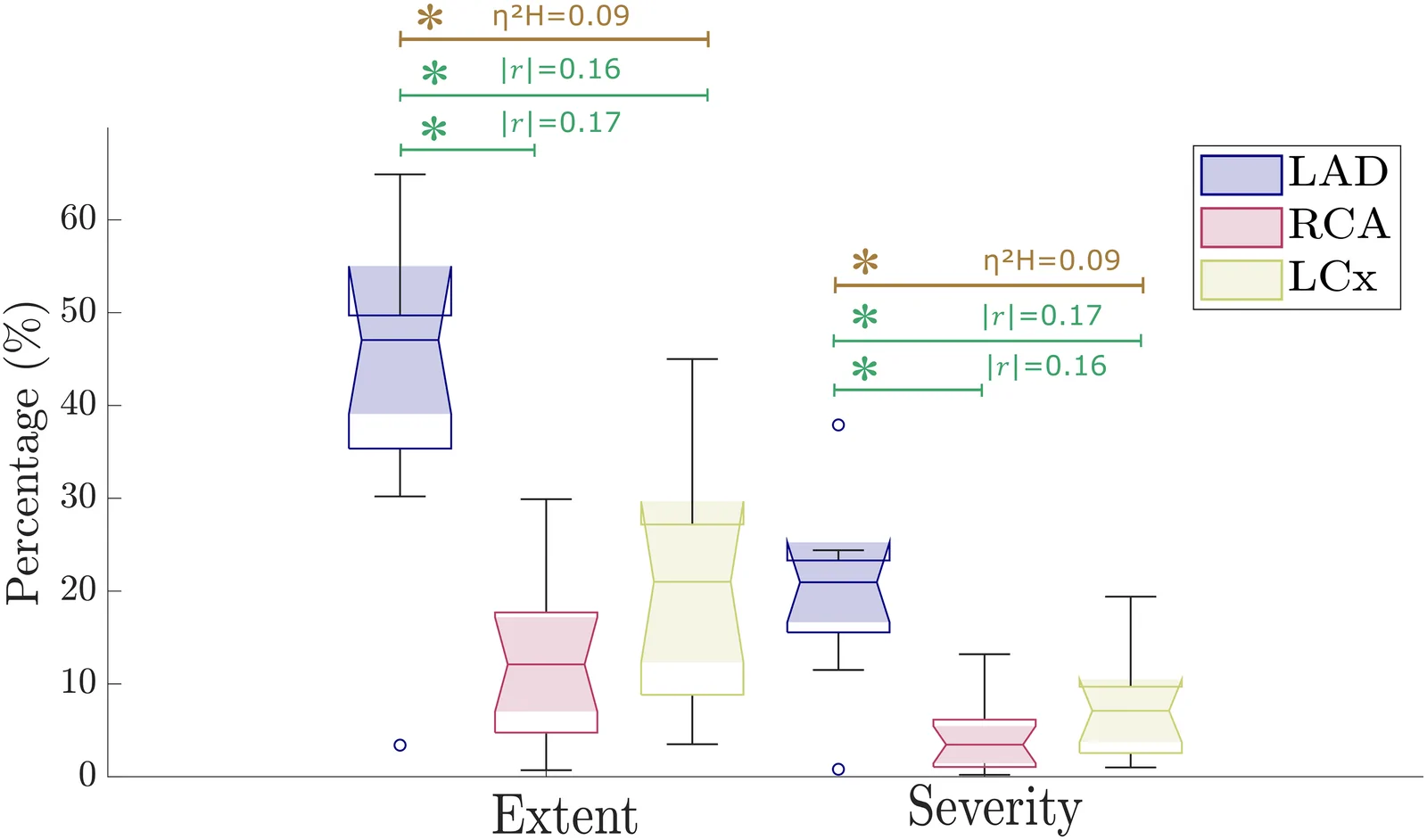
Box plots illustrating extent and severity of ischemia for patients with occlusions in the LAD, RCA, and LCx coronary arteries. Statistical significance of the comparisons between artery groups is shown for ischemia extent and severity. * denotes statistically significant differences. Effect size |*r*| and $\eta_{H}^{2}$ are reported.

Analyses excluding patients with prior MI are provided in the Supplementary Material. S5 Fig (S1 File) shows a similar overall pattern to Fig 9, although differences in $T_{am}$ may reflect greater heterogeneity among patients with prior MI. Similarly, S6 Fig (S1 File) shows patterns comparable to those in Fig 10, with some differences likely attributable to the lower number of patients included in the analysis. S7 Fig (S1 File) presents the temporal evolution of ECG markers during the first 4 minutes of occlusion for LAD, RCA, and LCX occlusions, while S8 Fig (S1 File) shows the corresponding analysis restricted to patients without prior MI. Overall, the differences in ECG changes among occlusion sites were consistent with the corresponding differences in ischemia extent and severity values, with LAD occlusion associated with the greatest magnitude of ischemia-induced changes across the evaluated ECG markers.

## Discussion

The study demonstrated that myocardial ischemia induces rapid and marked alterations in multiple ECG markers extending beyond the ST segment. These changes exhibit a clear temporal pattern, with repolarization-related markers showing earlier and larger responses than depolarization-related markers. Importantly, the magnitude and time course of these electrical alterations were not uniform but were significantly modulated by the underlying myocardial substrate, including sex, age, prior myocardial infarction, and occlusion location. Specifically, men showed larger and faster ECG changes, particularly in ST-based markers, while younger patients exhibited more sustained increases in repolarization markers. Patients with a prior MI displayed amplified and persistent elevations in both depolarization ($QRS_{ar}$, $QRS_{w}$) and repolarization markers, supporting their role as early electrical indicators of ischemic burden. Moreover, repolarization changes correlated more strongly and earlier with the extent and severity of ischemia than depolarization changes.

### Temporal evolution of ECG changes

Relative changes in ECG markers during ischemia were quantified using the normalized index $R_{\iota }(t_{j})$, which tracks acute ischemic deviations relative to each patient’s own physiological fluctuations. Unlike traditional absolute ECG measurements, which are inherently confounded by high inter-individual baseline variability, $R_{\iota }(t_{j})$ unmasks subtle temporal dynamics. Using this relative measure, we observed that ventricular repolarization markers responded more rapidly to ischemia than depolarization markers during the initial minutes of coronary occlusion. This observation aligns with the electrophysiological mechanisms underlying acute ischemia.

During ischemia, hypoxia-induced depletion of intracellular ATP activates $I_{K_{ATP}}$ channels, promoting *K*^+^ efflux and elevating the resting membrane potential. This reduces sodium channel availability, slows phase 0 depolarization, decreases conduction velocity, and accelerates phase 3 repolarization. The combined effect is a shortened action potential and reduced excitability within ischemic regions [5]. These electrophysiological changes manifest on the ECG as early and prominent ST-segment and T-wave alterations. In contrast, depolarization-related changes, reflected in QRS width, amplitude, and area, develop more gradually as conduction slowing and electrical dispersion progress, resulting in a delayed and less pronounced response.

Our results are consistent with this physiological framework. Ischemia-induced ECG changes first emerged in repolarization markers, followed by depolarization markers, supporting the concept of the ischemic cascade. Similar results were reported by Pueyo et al. [27], who identified early repolarization changes preceding QRS perturbations. In agreement with prior studies, QRS perturbations were preceded by ST-segment and T-wave modifications.

While previous work noted lead-dependent variability without distinguishing occluded arteries [27], our results demonstrate that lead-specific changes correspond to the anatomical differences among occluded vessels: frontal leads predominated in LAD occlusions, inferior leads in RCA occlusions, and no consistent pattern was observed for LCx occlusions. Additionally, [15] reported ischemia-related changes in QRS slopes, particularly a reduced downstroke steepness attributed to depolarization dispersion. These slope changes were linked to QRS widening or reduced $QRS_{am}$. In our study, both $QRS_{am}$ and $QRS_{w}$ were significantly altered during ischemia, with increasing divergence from control recordings as occlusion duration increased (Fig 3).

From a clinical perspective, these findings suggest that repolarization markers such as $ST_{dev}$, $ST_{ar}$, and *T*/*QRS* may serve as sensitive early indicators of acute myocardial ischemia, capturing electrical alterations within seconds of coronary occlusion. However, whether this rapid response translates into improved ischemia detection in clinical practice, particularly in emergency or pre-hospital settings, remains unknown. In contrast, the more gradual evolution of depolarization markers suggests a complementary role in tracking the progression of ischemia over time. Together, these observations indicate that combining early-responsive repolarization indices with more slowly evolving depolarization markers may provide complementary information for the detection and temporal characterization of ischemic events. Nevertheless, the clinical utility of such a combined approach remains to be studied in prospective clinical studies.

### Role of sex and age in ischemic ECG dynamics

#### Sex.

Men exhibited faster and larger ischemia-induced ECG changes, especially in ST-segment markers ($ST_{dev}$ and $ST_{ar}$). These findings are consistent with previous observations showing greater ST-segment deviation in men during angioplasty and acute MI [28]. Importantly, these differences occurred despite comparable ischemic extent and severity, indicating an intrinsic sex-related electrophysiological modulation rather than differences in ischemic burden.

Female sex has been identified as a predictor of mortality following STEMI [29]. However, the attenuated ECG response observed in women in our study may reflect a less extensive ischemic burden, as suggested by Fig 10. These sex-based differences have important diagnostic implications. Current guidelines recommend lower ST-elevation thresholds for women [30], and our findings support this adjustment, given the reduced magnitude of ischemia-induced ECG changes in women. Notably, guideline thresholds are based on absolute values rather than relative changes, as pre-ischemic ECGs are rarely available in emergency settings. Nonetheless, when prior tracings are available, baseline ECGs may be used to calculate relative changes, as low intraindividual variability in control recordings has been demonstrated [31,32].

Women are also less frequently referred for diagnostic testing and secondary prevention therapies [13,33]. While comorbidities may contribute, sex-specific ECG manifestations, such as less pronounced ST-segment changes, may partially explain under-recognition and under-treatment.

No significant sex differences were observed in ischemia extent, severity, or occluded artery distribution. Although men had a higher prevalence of prior MI, exclusion of these patients did not eliminate sex-related differences, supporting an independent biological contribution. Hormonal influences on depolarization and repolarization have been described [34] and may contribute to divergent ischemic responses, warranting further investigation.

These results suggest that fixed, absolute ECG thresholds may not fully capture sex-related differences in the magnitude and temporal evolution of ischemia-induced ECG changes. This observation is consistent with current guideline recommendations for sex-specific ST-elevation criteria and suggests that incorporating relative or dynamic ECG measures warrants further investigation as a means of improving diagnostic sensitivity. Validation in larger and more diverse cohorts is required to determine whether these differences have implications for the diagnosis and management of acute coronary syndromes in female patients.

#### Age.

Age is a well-established predictor of short- and long-term mortality after MI and is incorporated into several risk stratification tools [35]. Older patients are also known to present with more nondiagnostic ECG findings [36]. Accordingly, the diagnostic criteria for STEMI include age-adjusted ST-elevation thresholds, particularly in anterior leads for men.

In our study, older patients exhibited a rapid initial ECG response to ischemia, followed by a slower rate of change, whereas younger patients demonstrated a more gradual and sustained increase over time. This attenuation may reflect age-related limitations in myocardial responsiveness or the higher burden of comorbidities commonly observed in older populations [36].

The distinct temporal dynamics observed across age groups suggest that clinical interpretation should consider not only the magnitude but also the rate of change of ischemia-induced alterations. The more abrupt but transient responses in older patients may increase the likelihood of missed or delayed diagnoses if ECG recordings are not obtained early, whereas the steady and more sustained progression in younger patients may provide a wider diagnostic window. Continuous, time-dependent ECG monitoring is therefore critical to capture these divergent dynamic profiles, particularly in older individuals where ischemic patterns plateau rapidly.

### Role of occluded artery in acute ischemic ECG dynamics

Ischemia-induced ECG changes varied according to the location of the occluded coronary artery. Patients with LAD occlusion demonstrated the widest range of ECG changes, likely reflecting their substantially greater ischemic extent and severity. As shown in Fig 10, median extent and severity values in LAD occlusions were more than threefold higher than in other arteries, with RCA occlusions showing the lowest values.

These findings are consistent with prior studies reporting ST-segment deviations and QRS angle changes during coronary occlusion [37,38]. In line with previous observations, LAD occlusions primarily affected anterior leads, RCA occlusions affected inferior leads, and LCx occlusions exhibited less consistent patterns. The limited detectability of LCx ischemia likely reflects the lack of standard leads oriented toward the posterior left ventricular wall, supporting recommendations to record posterior leads when LCx infarction is suspected [30,39].

In terms of their potential clinical relevance, our results indicate that quantitative ECG markers capture differences related to the anatomical location of coronary occlusion. The more pronounced and spatially localized changes observed in LAD occlusions suggest that the analyzed ECG indices may contain information relevant to identifying the affected coronary territory. Conversely, the less distinctive patterns associated with LCx occlusion are consistent with known limitations of the standard 12-lead ECG and support the potential value of additional posterior leads or complementary diagnostic tools when LCx involvement is suspected.

### Role of prior MI and ischemic extent and severity in acute ischemic ECG dynamics

Previous MI is known to alter baseline ECG morphology, particularly through Q waves and T-wave inversions [30]. However, its impact on ECG responses during acute ischemia remains poorly understood. Our results reveal a modest trend toward more pronounced and sustained ischemia-induced ECG responses in patients with a prior MI compared to those without, even when ischemic extent and severity are similar.

Contrary to our initial hypothesis that scar-related nonviable myocardium would attenuate ischemic responses, patients with prior MI exhibited a modest yet consistent increase in changes, particularly in repolarization markers. This finding suggests that infarct-related structural remodeling may potentiate electrical sensitivity to acute ischemia. This observation is consistent with prior reports of increased T-wave alternans during ischemia in patients with prior MI, reflecting greater temporal and/or spatial repolarization dispersion [40].

A plausible mechanistic explanation is the presence of a heterogeneous border zone surrounding infarcted tissue, characterized by irregular fibrosis distribution and altered conduction properties [41]. Such structural and electrophysiological heterogeneity may amplify electrical dispersion during superimposed ischemia, thereby increasing the magnitude of both repolarization and depolarization alterations. Additionally, patients with prior MI often have higher rates of other comorbidities, including hypertension and diabetes, which may further modulate the ECG response to ischemia [42,43].

Collectively, these findings indicate that while the pre-existing myocardial substrate may modulate the electrical expression of acute ischemia, this modulating effect remains modest. Nevertheless, these observations suggest that relative changes in ECG markers may remain informative even in patients with pre-existing ECG abnormalities due to prior MI. This may be particularly relevant for characterizing ischemia-induced ECG changes in the presence of an altered baseline ECG. Moreover, the modest amplification of ischemia-induced responses observed in these patients may reflect increased electrical vulnerability, potentially providing additional insight into the electrophysiological consequences of prior MI. However, further validation in larger patient populations and in other clinical settings is needed before the clinical significance and potential utility of these findings can be established.

## Limitations and future work

Several limitations should be considered when interpreting the present findings:

- A limitation of this study is the reliance on a baseline ECG acquired prior to coronary occlusion, which may not always be available in routine clinical practice. Although alternative baseline recordings may be obtained from the earliest ECG recorded during patient transport or hospital admission, or from previous ECG examinations, such recordings may not fully represent a true pre-ischemic state. The present approach may therefore be most applicable in settings where serial ECG recordings are available, allowing assessment of intra-individual changes over time. While inter-individual variability limits direct comparison of marker values across patients, the relatively stable behavior of these markers within individuals suggests potential utility for longitudinal ischemia assessment. However, the feasibility and clinical value of this approach require further evaluation in real-world settings.
- The sample size is relatively small, reflecting the limited availability of this specific type of ECG recordings, which were collected in the early 2000s. Nevertheless, this database represents a unique human model of nearly five minutes of controlled ischemia induced during PCI. Given the small size of certain subcohorts, including patients with LAD artery occlusions (*n* = 34, 33.3%) and those with prior MI (*n* = 34, 33.3%), as well as the subgroup for whom information on ischemia extent and severity was available, the statistical findings, in particular those derived from subgroup and multivariate analyses, should be interpreted with appropriate caution.
- Occlusions occurred at varying anatomical levels (proximal, mid, or distal), and data on the extent of ischemia were unavailable for a substantial proportion of patients. These procedural differences may have influenced the magnitude and temporal evolution of the ECG response and could not be fully accounted for in the present analysis.
- Potential confounders, including left ventricular hypertrophy, left anterior fascicular block, long QT syndrome, other comorbidities, and pharmacological therapy, may have influenced the observed results. Because the investigated markers were evaluated relative to each patient’s own baseline ECG, these factors were present in both recordings, and their impact may therefore have been partially mitigated. Nevertheless, it remains possible that comorbidities and medication influenced the electrophysiological response to acute ischemia. Further studies are needed to assess the effects of these factors on the proposed ECG markers.
- Information regarding medical therapy in patients with a prior MI, as well as the timing of the previous infarction relative to the ischemic event, was not available, limiting a more detailed assessment of their potential influence on the ECG response.
- As noted above, the recordings used in this study originate from a highly specific human model of acute ischemia induced during PCI, which enabled controlled assessment of ischemia-related ECG changes. However, the controlled nature of this model may limit the generalizability of the findings to routine clinical practice. External validation in a comparable independent cohort is currently challenging, as datasets including similarly prolonged, controlled coronary occlusions during PCI are not readily available under current clinical practice. Further studies in contemporary real-world clinical settings and larger patient cohorts are therefore needed to confirm the robustness, generalizability, and applicability of these observations.
- Although recordings were acquired in the supine position, in which the standard 12-lead ECG may represent cardiac orientation differently than in the standing position, this factor is unlikely to account for the artery-specific spatial patterns observed in the present study.
- Finally, the documented location of the prior MI was less precise than that of the acute ischemic event, limiting detailed interpretation.

Beyond these methodological considerations, the present findings also point toward several potential avenues for clinical translation. The use of normalized indices such as $R_{\iota }$, which account for baseline variability, may facilitate the assessment of intra-individual ECG changes compared with conventional fixed thresholds. In addition, the integration of multiple ECG markers capturing both depolarization and repolarization processes may provide complementary information on the temporal evolution of acute ischemia. More broadly, combining ECG-derived features with clinical variables such as age, sex, and prior MI may support the development of multivariable models for improved characterization of acute ischemia. Further prospective studies in larger patient cohorts and across diverse clinical settings are warranted to determine the diagnostic utility and incremental value of these approaches in real-world practice.

## Conclusions

By comparing ECG recordings obtained before and during PCI-induced ischemia, we show that acute myocardial ischemia produces significant alterations not only in ST-segment deviation but also in multiple ECG markers reflecting ventricular depolarization and repolarization. The magnitude and temporal dynamics of these changes vary according to the occlusion site, patient sex, and the presence of a prior MI. Furthermore, ischemia-induced ECG modifications correlate with both the extent and severity of ischemia, with this relationship potentially more pronounced in patients with a history of MI.

## Supporting information

S1 File Supplementary Figures S1–S8. (PDF)
