# Supplementary material for: Characterizing acute ischemia via ECG: A study of severity, extent, and spatial variability in myocardial infarction and non-infarction groups
Source: PLoS One. 2026 Oct 29;21(10):e0361220. doi: 10.1371/journal.pone.0361220 (PMC13719058; doi:10.1371/journal.pone.0361220)
Supplement: Supplementary file 1 [file pone.0361220.s001.pdf]

Characterizing acute ischemia via ECG: a study of severity, extent, and spatial variability in myocardial infarction and non-infarction groups

Supplementary Information

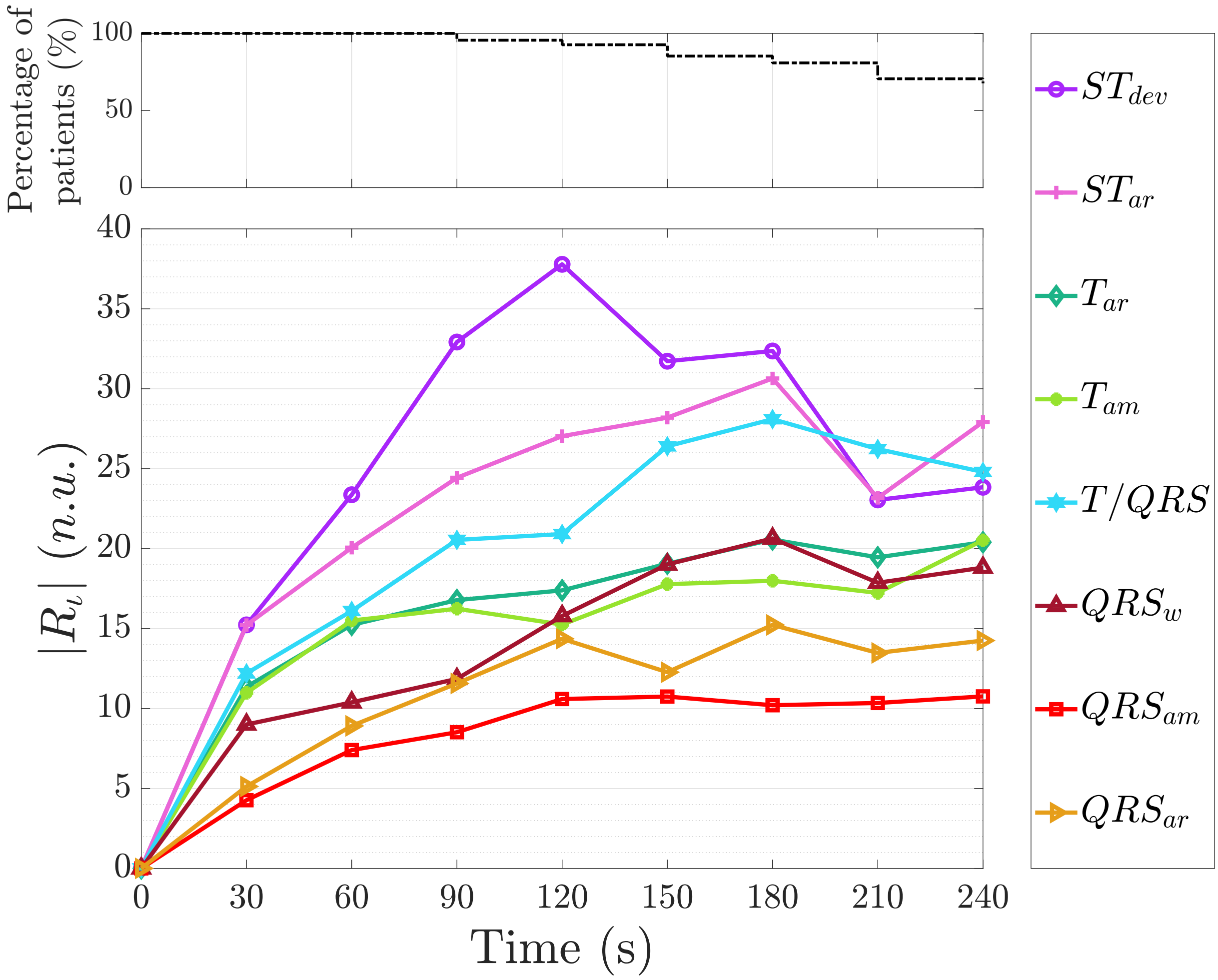

S1_Fig. **Temporal evolution of the ECG markers during the first 4 minutes of coronary occlusion across patients without a prior MI.** Top: Percentage of patients remaining under occlusion over a 4-minute period. Bottom: Median $|R_\iota|$ values across patients without a prior MI, with each patient's value computed from the 90th percentile of the 12 analyzed ECG leads. Values are reported every 30 seconds from the onset of occlusion.

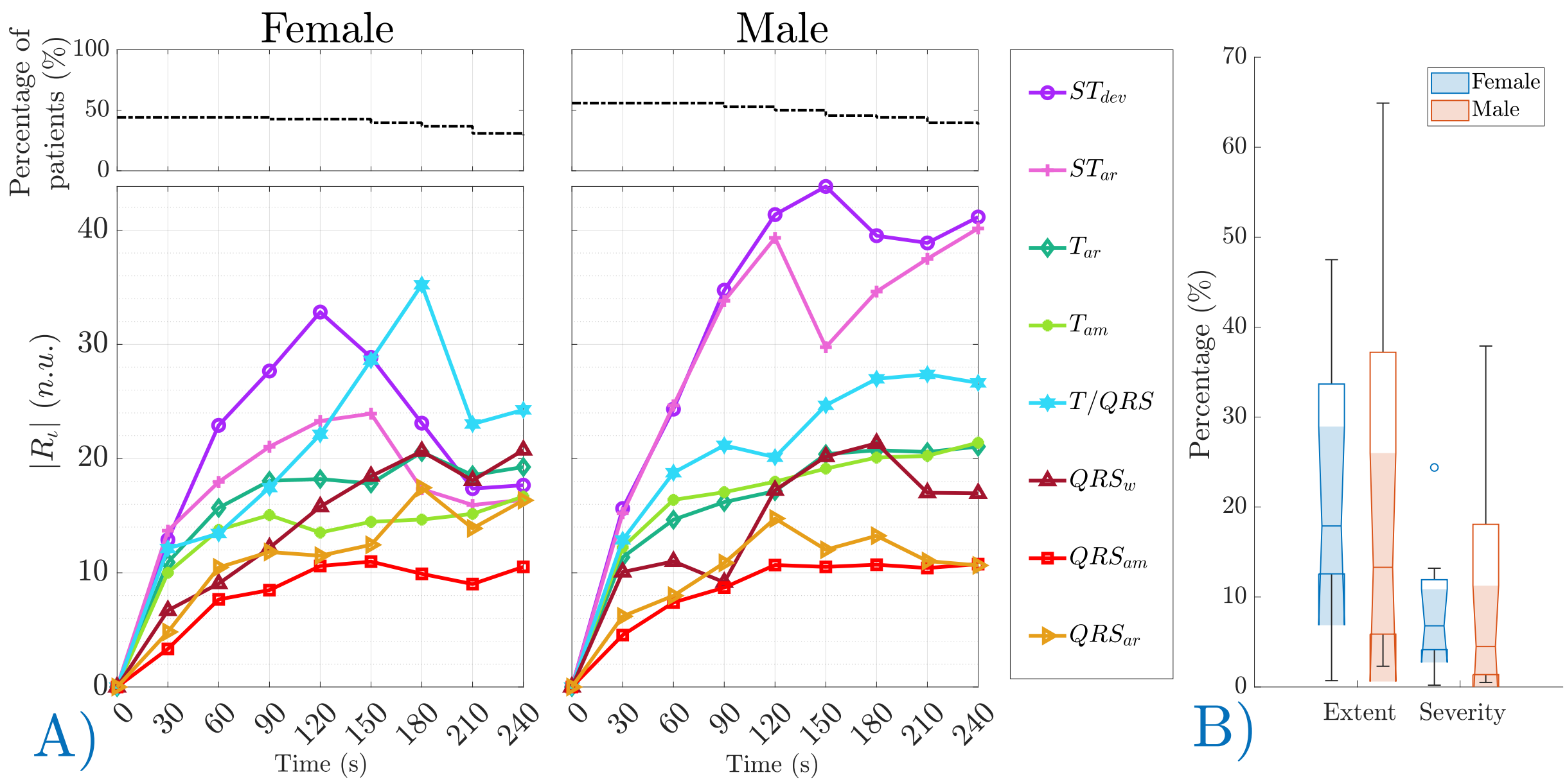

S2_Fig. **Temporal evolution of the ECG markers during the first 4 minutes of coronary occlusion across patients without a prior MI, stratified by sex.** A) Top: Percentage of patients under occlusion over 4 minutes. Bottom: Median $|R_\iota|$ values across patients without a prior MI, computed from the 90th percentile of the 12 analyzed leads and reported every 30 seconds from the onset of occlusion, shown separately for female (left) and male (right) patients. B) Box plots of the extent and severity of ischemia for female and male patients without a prior MI.

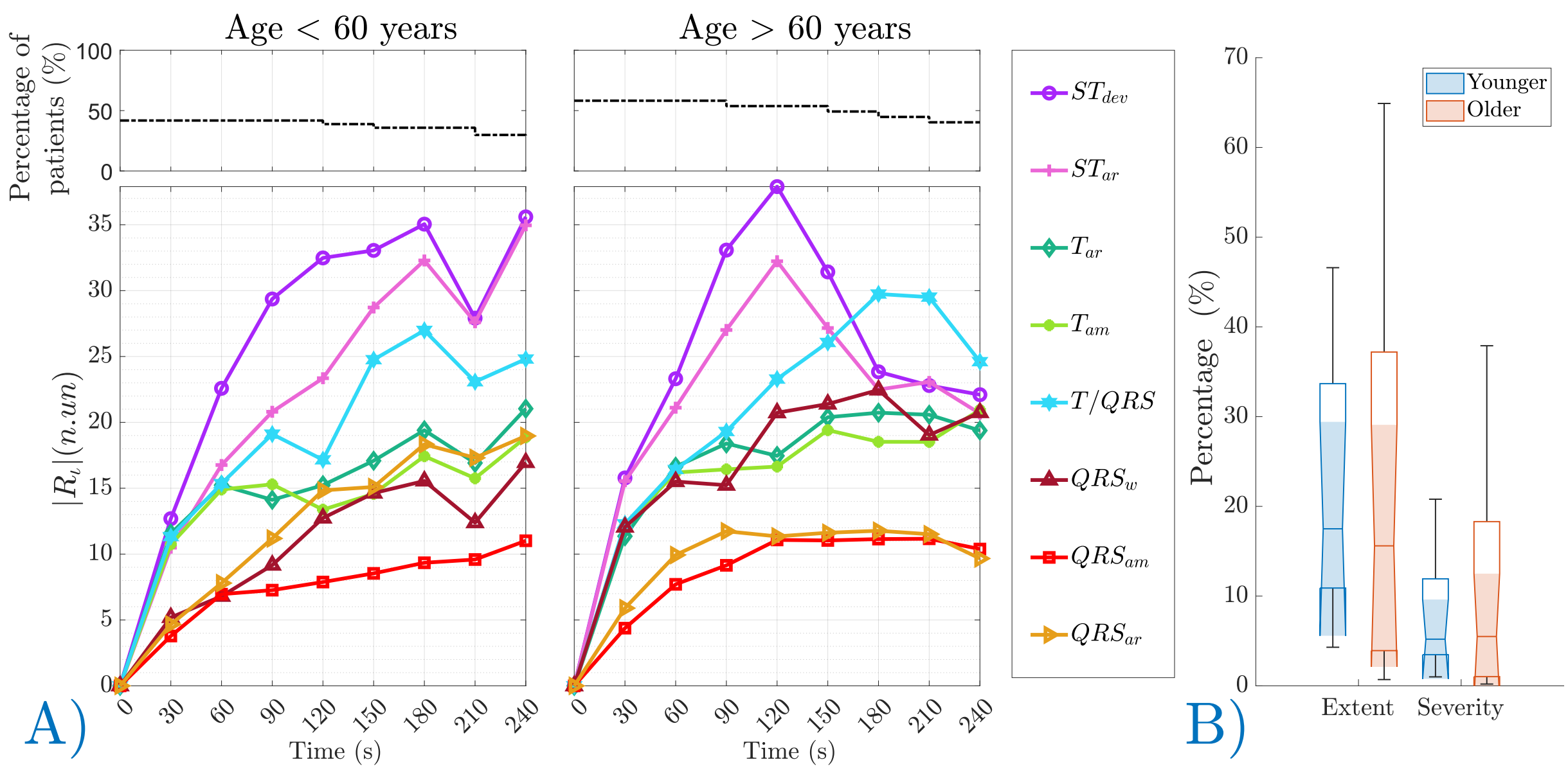

S3_Fig. **Temporal evolution of the ECG markers during the first 4 minutes of coronary occlusion across patients without a prior MI, stratified by age.** A) Top: Percentage of patients under occlusion over 4 minutes. Bottom: Median $|R_\iota|$ values across patients without a prior MI, computed from the 90th percentile of the 12 analyzed leads and reported every 30 seconds from the onset of occlusion, shown separately for younger (age < 62 years) and older (age ≥ 62 years) patients. B) Box plots of the extent and severity of ischemia for younger and older patients without a prior MI.

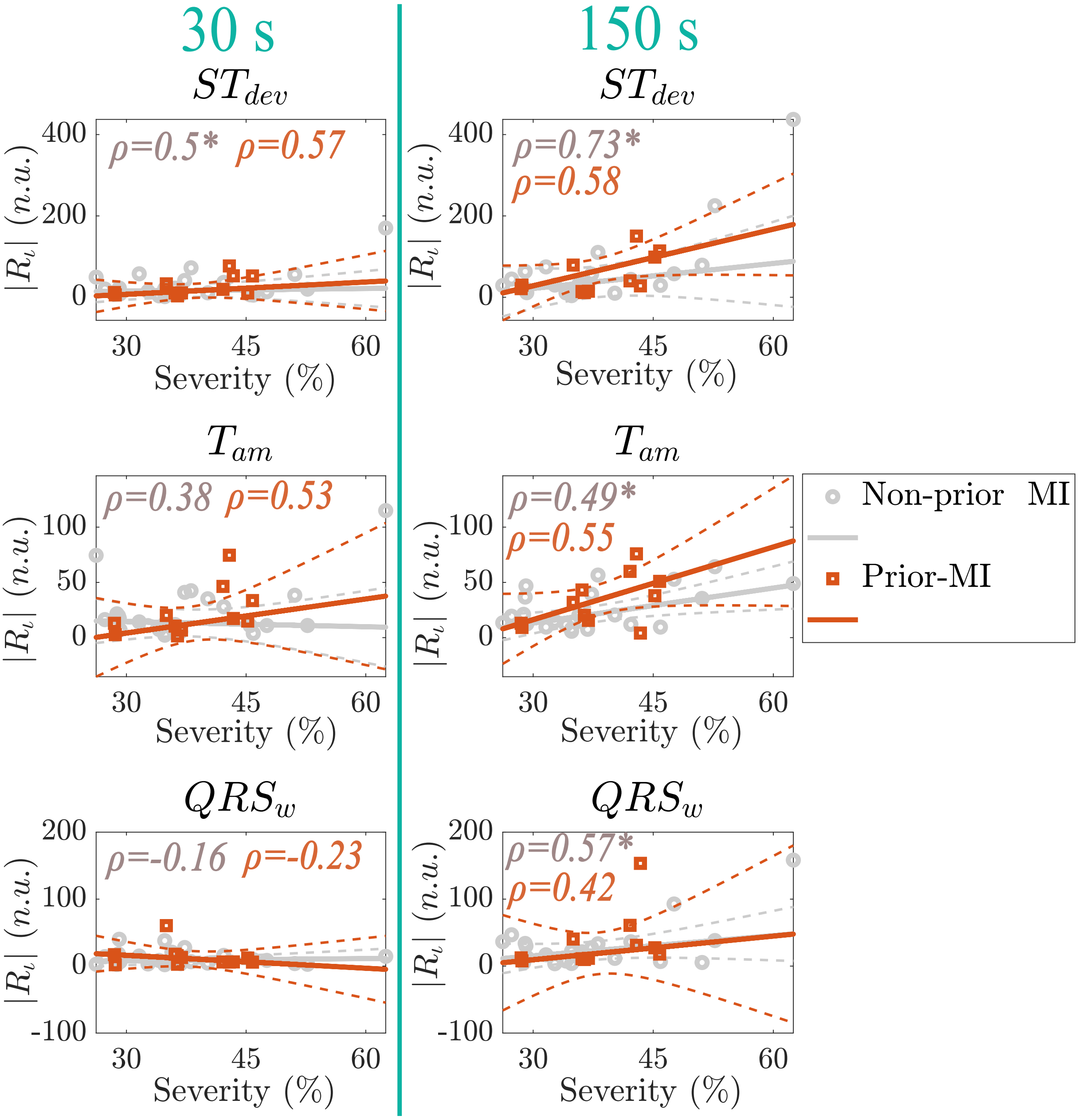

S4_Fig. **Scatter plots showing the relationship between the severity of ischemia and $|R_\iota|$ for ST_{dev}, T_{am}, and QRS_w.** The $|R_\iota|$ values are calculated as the 90th percentile across the 12 leads for each patient at 30 seconds (left panels) and 150 seconds after the onset of occlusion (right panels). Results are shown for patients with prior MI (red squares) and without prior MI (gray circles). The solid and dotted lines depict the linear regression and its corresponding 95% confidence interval, respectively. ρ values of the correlations are reported; * corresponds to significant correlations ($p<0.05$).

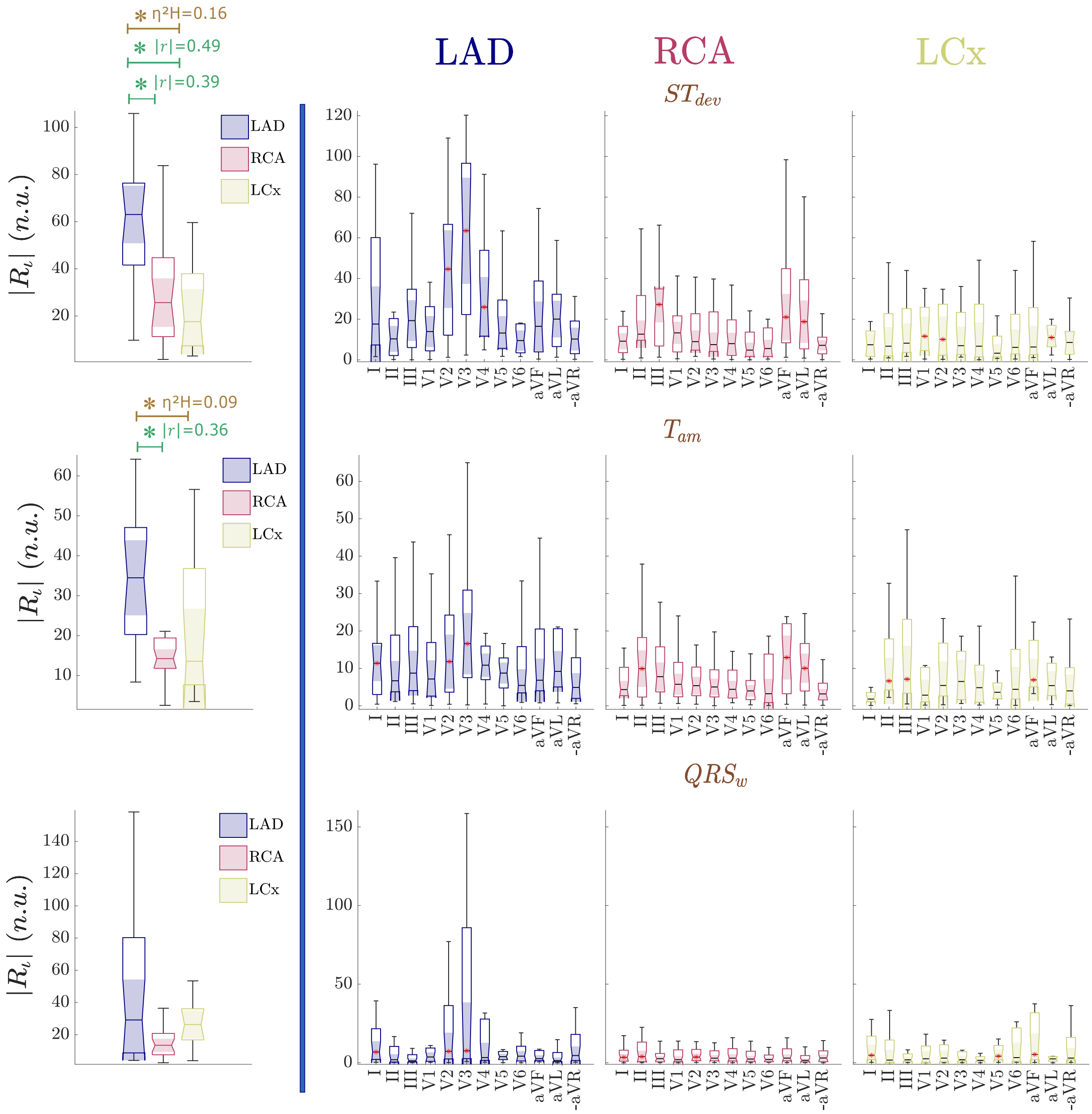

S5_Fig. **Box plots of $|R_\iota|$ stratified by LAD, RCA, and LCx occlusions across patients without prior MI.** Left: Box plots of $|R_\iota|$ across patients without a prior MI, computed from the 90th percentile of the 12 analyzed leads at 150 seconds from the onset of occlusion for ST_{dev}, T_{am}, and QRS_w, stratified by LAD, RCA, and LCx occlusions. * denotes statistically significant differences. Effect size $|r|$ and η_H^2 are reported. Right: Lead-wise $|R_\iota|$ values across patients without a prior MI at 150 seconds from the onset of occlusion for all 12 analyzed leads, shown for ST_{dev}, T_{am}, and QRS_w for each occluded artery.

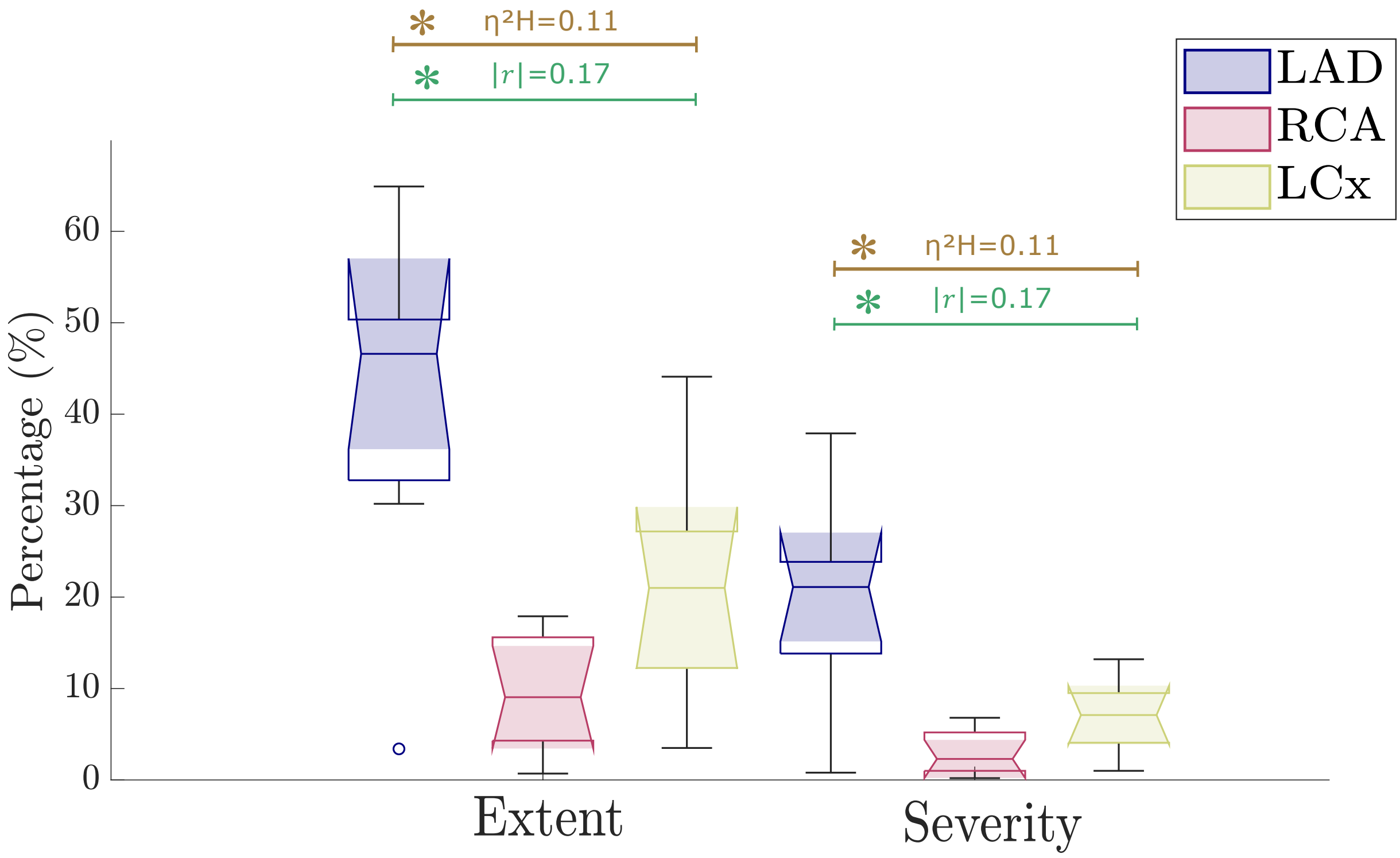

S6_Fig. **Box plots of the extent and severity of ischemia for patients without a prior MI and with occlusions in the LAD, RCA, and LCx coronary arteries.** * denotes statistically significant differences. Effect size $|r|$ and η^2_H are reported.

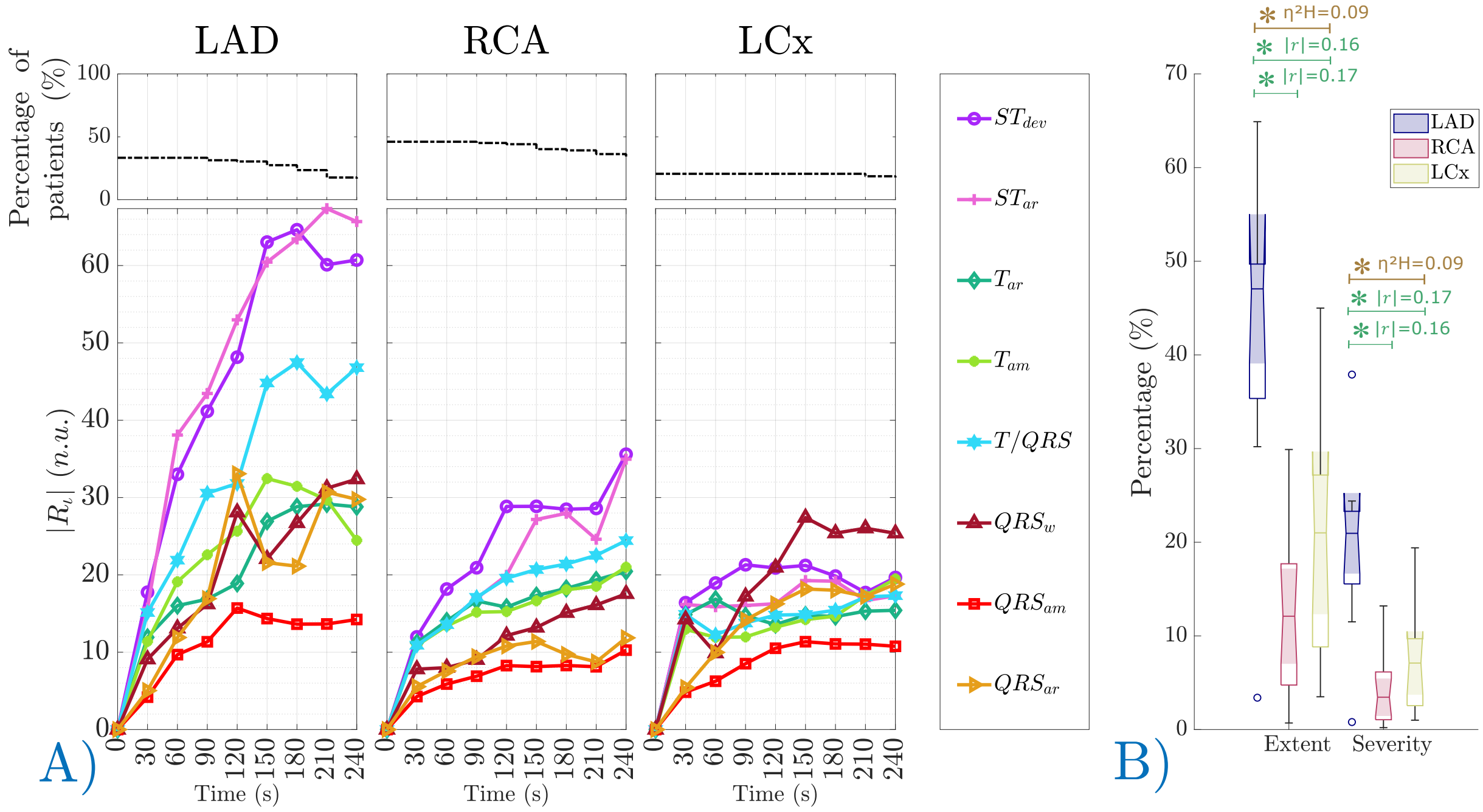

S7_Fig. **Temporal evolution of the ECG markers during the first 4 minutes of coronary occlusion, stratified by the occluded artery.** A) Top: Percentage of patients under occlusion over a 4-minute period. Below: Median $|R_\iota|$ values across all patients, with patient-specific values computed from the 90th percentile of the 12 analyzed leads. Values are reported every 30 seconds from the onset of the occlusion for patients with LAD (right), RCA (middle), and LCx (left) occlusions. B) Box plots of the extent and severity of ischemia for patients grouped by the occluded coronary artery. * denotes statistically significant differences. Effect size $|r|$ and η_H^2 are reported.

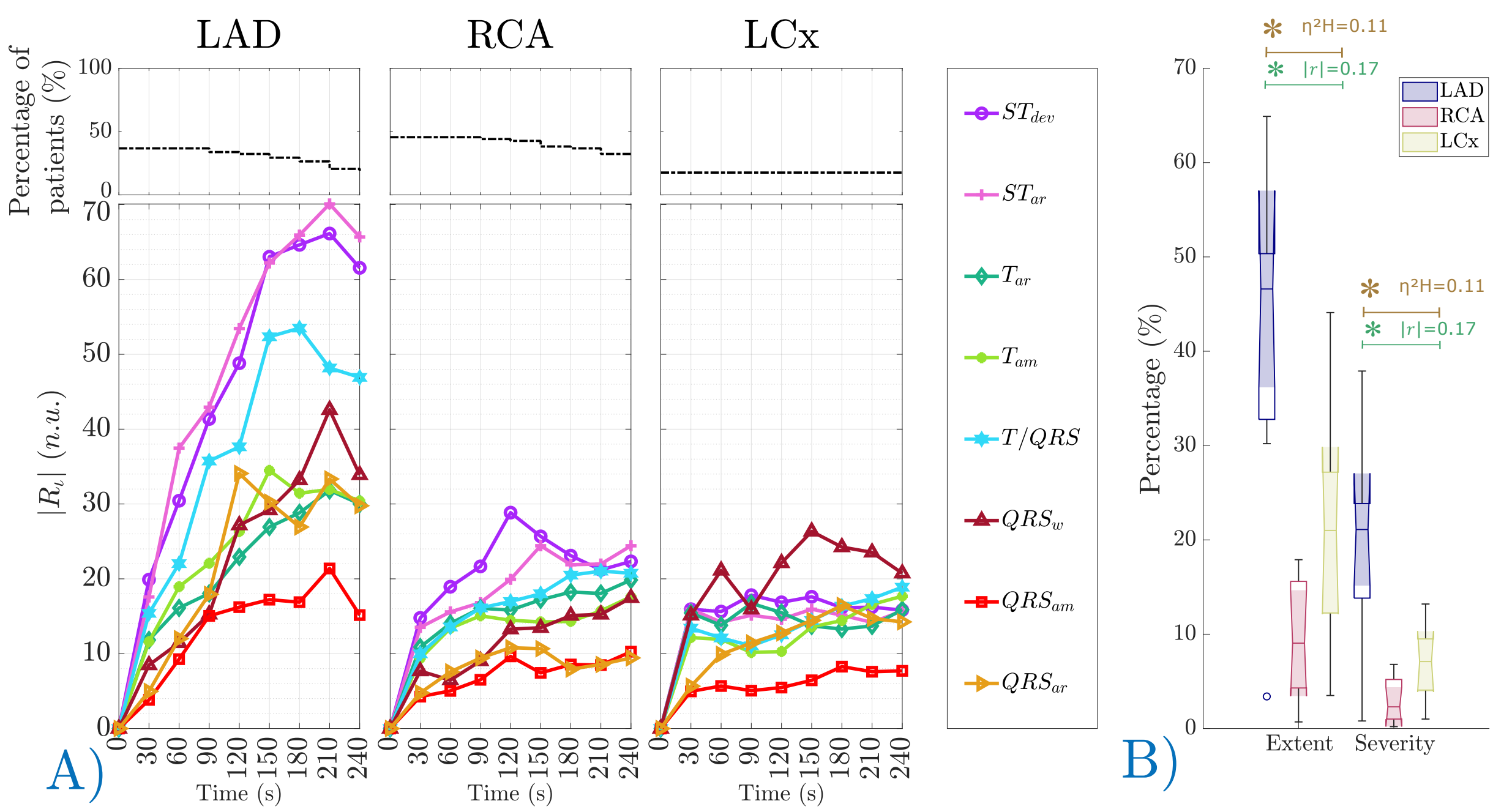

S8_Fig. **Temporal evolution of the ECG markers during the first 4 minutes of coronary occlusion across patients without a prior MI, stratified by occluded artery.** A) Top: Percentage of patients under occlusion over a 4-minute period. Below: Median $|R_\iota|$ values across patients without a prior MI, with patient-specific values computed from the 90th percentile of the 12 analyzed leads. Values are reported every 30 seconds from the onset of the occlusion for patients with LAD (left), RCA (middle), and LCx (right) occlusions. B) Box plots of the extent and severity of ischemia for patients without a prior MI, grouped by the occluded coronary artery. * denotes statistically significant differences. Effect size $|r|$ and η_H^2 are reported.
