# Supplementary material for: Characterizing acute ischemia via ECG: A study of severity, extent, and spatial variability in myocardial infarction and non-infarction groups
Source: PLoS One. 2026 Oct 29;21(10):e0361220. doi: 10.1371/journal.pone.0361220 (PMC13719058; doi:10.1371/journal.pone.0361220)
Supplement: Supplementary file 2 [file pone.0361220.s003.pdf]

June 25, 2026

Resubmission of manuscript: “Characterizing acute ischemia via ECG: a study of severity, extent, and spatial variability in myocardial infarction and non-infarction groups” with reference PONE-D-26-08831

Jimena Rodríguez-Carbó
University of Zaragoza
Zaragoza, Spain
jimena.rodriguez@unizar.es

Editor in Chief
PLOS One

Dear Editor,

We would like to thank you for the opportunity to revise our manuscript, “Characterizing acute ischemia via ECG: a study of severity, extent, and spatial variability in myocardial infarction and non-infarction groups”. We appreciate the review and the constructive suggestions provided by the Reviewers. We believe the manuscript has been strengthened by incorporating these modifications.

Journal Requirements:

1. When submitting your revision, we need you to address these additional requirements. Please ensure that your manuscript meets PLOS ONE’s style requirements, including those for file naming. The PLOS ONE style templates can be found at `https://journals.plos.org/plosone/s/file?id=wjVg/PLOSOne_formatting_sample_main_body.pdf` **and** `https://journals.plos.org/plosone/s/fileid=ba62/PLOSOne_formatting_sample_title_authors_affiliations.pdf`**.**

We made the corresponding modifications to meet the requirements. We modified the names of the figures within the text. References were moved to a section after the Acknowledgments.

---

2. Please note that PLOS One has specific guidelines on code sharing for submissions in which author-generated code underpins the findings in the manuscript. In these cases, we expect all author-generated code to be made available without restrictions upon publication of the work. Please review our guidelines at `https://journals.plos.org/plosone/s/materials-and-software-sharing#loc-sharing-code` **and ensure that your code is shared in a way that follows best practice and facilitates reproducibility and reuse.**

We made the software used to compute the values of the R index, which is used to present the results along the text, available in Github `https://github.com/JRDZC/R_index.git`.

---

3. We note that the grant information you provided in the ‘Funding Information’ and ‘Financial Disclosure’ sections do not match. When you resubmit, please ensure that you provide the correct grant numbers for the awards you received for your study in the ‘Funding Information’ section. In both sections, the text reads:

Thank you for pointing this out. We corrected the grant information to ensure consistency between the ‘Funding Information’ and ‘Financial Disclosure’ sections.

---

4. Thank you for stating in your Funding Statement:

This work was supported by projects PID2022-140556OB-I00 and PID2023-148975OB-I00 (`https://www.aei.gob.es/convocatorias/buscador-convocatorias/proyectos-generacion-conocimiento-2022/convocatoria`) awarded to EP and AM, CNS2022-135899 (`https://www.aei.gob.es/convocatorias/buscador-convocatorias/consolidacion-investigadora-2022`) awarded to AM, and TED2021-130459B-I00 funded by `MCIN/AEI/10.13039/501100011033` and ”ERDF A way of making Europe” (`https://www.aei.gob.es/convocatorias/buscador-convocatorias/proyectos-estrategicos-orientados-transicion-ecologica`) awarded to AM, EP, and JRC. The study was also supported by fellowship RYC2019-027420-I funded by the Ramón y Cajal Program (`https://www.aei.gob.es/convocatorias/buscador-convocatorias/ayudas-contratos-ramon-cajal-ryc-2019`) awarded to AM, by the BSICoS group T39_23R funded by the Aragón Government and FEDER 2014-2020 “Building Europe from Aragon” (`https://www.aragon.es/fondos-europeos/feder-fondo

-de-desarrollo-regional/2014-2020) awarded to AM, EP, and JRC, and by the PhD fellowship "Ayudas para iberoamericanos y ecuatoguineanos en Estudios de Doctorado - Curso 2023/2024" funded by the University of Zaragoza and Banco Santander (https://internacional.unizar.es/convocatoria-de-ayudas-para-iberoamericanos-y-ecuatoguineanos-en-estudios-de-doctorado-2324) awarded to JRC.

Please provide an amended statement that declares *all* the funding or sources of support (whether external or internal to your organization) received during this study, as detailed online in our guide for authors at http://journals.plos.org/plosone/s/submit-now. Please also include the statement "There was no additional external funding received for this study." in your updated Funding Statement. Please include your amended Funding Statement within your cover letter. We will change the online submission form on your behalf.

We added the statements at the end of the financial disclousure statement.

This work was supported by projects PID2022-140556OB-I00 and PID2023-148975OB-I00 (https://www.aei.gob.es/convocatorias/buscador-convocatorias/proyectos-generacion-conocimiento-2022/convocatoria) awarded to EP and AM, CNS2022-135899 (https://www.aei.gob.es/convocatorias/buscador-convocatorias/consolidacion-investigadora-2022) awarded to AM, and TED2021-130459B-I00 funded by MCIN/AEI/10.13039/501100011033 and "ERDF A way of making Europe" (https://www.aei.gob.es/convocatorias/buscador-convocatorias/proyectos-estrategicos-orientados-transicion-ecologica) awarded to AM, EP, and JRC. The study was also supported by fellowship RYC2019-027420-I funded by the Ramón y Cajal Program (https://www.aei.gob.es/convocatorias/buscador-convocatorias/ayudas-contratos-ramon-cajal-ryc-2019) awarded to AM, by the BSICoS group T39_23R funded by the Aragón Government and FEDER 2014-2020 "Building Europe from Aragon" (https://www.aragon.es/fondos-europeos/feder-fondo-de-desarrollo-regional/2014-2020) awarded to AM, EP, and JRC, and by the PhD fellowship "Ayudas para iberoamericanos y ecuatoguineanos en Estudios de Doctorado - Curso 2023/2024" funded by the University of Zaragoza and Banco Santander (https://internacional.unizar.es/convocatoria-de-ayudas-para-iberoamericanos-y-ecuatoguineanos-en-estudios-de-doctorado-2324) awarded to JRC. There was no additional external funding received for this study. The funders had no role in study design, data collection and analysis, decision to publish, or preparation of the manuscript.

---

5. Thank you for stating the following financial disclosure: This work was supported by projects PID2022-140556OB-I00 and PID2023-148975OB-I00 (https://www.aei.gob.es/convocatorias/buscador-convocatorias/proyectos-generacion-conocimiento-2022/convocatoria) awarded to EP and AM, CNS2022-135899 (https://www.aei.gob.es/convocatorias/buscador-convocatorias/consolidacion-investigadora-2022) awarded to AM, and TED2021-130459B-I00 funded by MCIN/AEI/10.13039/501100011033 and "ERDF A way of making Europe" (https://www.aei.gob.es/convocatorias/buscador-convocatorias/proyectos-estrategicos-orientados-transicion-ecologica) awarded to AM, EP, and JRC. The study was also supported by fellowship RYC2019-027420-I funded by the Ramón y Cajal Program (https://www.aei.gob.es/convocatorias/buscador-convocatorias/ayudas-contratos-ramon-cajal-ryc-2019) awarded to AM, by the BSICoS group T39_23R funded by the Aragón Government and FEDER 2014-2020 "Building Europe from Aragon" (https://www.aragon.es/fondos-europeos/feder-fondo-de-desarrollo-regional/2014-2020) awarded to AM, EP, and JRC, and by the PhD fellowship "Ayudas para iberoamericanos y ecuatoguineanos en Estudios de Doctorado - Curso 2023/2024" funded by the University of Zaragoza and Banco Santander (https://internacional.unizar.es/convocatoria-de-ayudas-para-iberoamericanos-y-ecuatoguineanos-en-estudios-de-doctorado-2324) awarded to JRC.

Please state what role the funders took in the study. If the funders had no role, please state: "The funders had no role in study design, data collection and analysis, decision to publish, or preparation of the manuscript." If this statement is not correct you must amend it as needed. Please include this amended Role of Funder statement in your cover letter; we will change the online submission form on your behalf.

We added the statement "The funders had no role in study design, data collection and analysis, decision to publish, or preparation of the manuscript." to the Funding statement and added the amended Role of Funders' statement in the cover letter.

---

6. We noted in your submission details that a portion of your manuscript may have been presented or published elsewhere. [Preliminary results of this study were presented in the

Computing in Cardiology Conference in 2023 and published in the conference's proceedings. These results and images were improved by using different metrics and further analyses were computed to create the study presented here. The results presented in the conference were uploaded in the Attached Files.] Please clarify whether this [conference proceeding or publication] was peer-reviewed and formally published. If this work was previously peer-reviewed and published, in the cover letter please provide the reason that this work does not constitute dual publication and should be included in the current manuscript.

Preliminary results from this study were presented at the Computing in Cardiology Conference in 2023 and published in `https://ieeexplore.ieee.org/abstract/document/10364136`. The conference presentation was selected on the basis of a short abstract of approximately 300 words, and the proceedings publication did not undergo peer review. Also, it should not be considered a dual publication because the previously published results were preliminary. The present manuscript includes improved signal preprocessing, new metrics, and additional analyses, including the assessment of ECG differences in response to induced ischemia according to sex, age, and prior myocardial infarction.

---

7. If the reviewer comments include a recommendation to cite specific previously published works, please review and evaluate these publications to determine whether they are relevant and should be cited. There is no requirement to cite these works unless the editor has indicated otherwise.

[Note: HTML markup is below. Please do not edit.]

Reviewers' comments:
Reviewer's Responses to Questions

Comments to the Author

1. Is the manuscript technically sound, and do the data support the conclusions?

The manuscript must describe a technically sound piece of scientific research with data that supports the conclusions. Experiments must have been conducted rigorously, with appropriate controls, replication, and sample sizes. The conclusions must be drawn appropriately based on the data presented.

Reviewer #1: Yes
Reviewer #2: No

---

2. Has the statistical analysis been performed appropriately and rigorously?

Reviewer #1: Yes
Reviewer #2: No

---

3. Have the authors made all data underlying the findings in their manuscript fully available?

The PLOS Data policy requires authors to make all data underlying the findings described in their manuscript fully available without restriction, with rare exception (please refer to the Data Availability Statement in the manuscript PDF file). The data should be provided as part of the manuscript or its supporting information, or deposited to a public repository. For example, in addition to summary statistics, the data points behind means, medians and variance measures should be available. If there are restrictions on publicly sharing data—e.g. participant privacy or use of data from a third party—those must be specified.

Reviewer #1: Yes
Reviewer #2: No

---

4. Is the manuscript presented in an intelligible fashion and written in standard English?

PLOS ONE does not copyedit accepted manuscripts, so the language in submitted articles must be clear, correct, and unambiguous. Any typographical or grammatical errors should be corrected at revision, so please note any specific errors here.

Reviewer #1: Yes
Reviewer #2: No

---

5. Review Comments to the Author Please use the space provided to explain your answers to the questions above. You may also include additional comments for the author, including concerns about dual publication, research ethics, or publication ethics. (Please upload your review as an attachment if it exceeds 20,000 characters)

Reviewer #1:

We are grateful to the Reviewer for the revision and for the helpful and constructive comments, which we addressed as follows.

Quite an interesting manuscript regarding ischemia induced ECG changes beyond the ST-T deviation. According to the authors, beyond ST-segment deviation, multiple ECG markers evolve over time during coronary artery occlusion. Repolarization features showed earlier and more pronounced alterations than depolarization features, reaching approximately 37-fold and 23-fold changes at 4 minutes of occlusion compared with baseline, respectively. Males showed faster, sustained ST increases (up to ∼ 47-fold vs ∼ 30-fold in females), while older patients had abrupt early ST peaks (∼ 16-fold at ∼ 2 min after occlusion), whereas younger patients exhibited a more gradual increase. Moreover, patients with a prior infarction showed a stronger association between ECG changes and both the extent and severity of ischemia, reaching maximum correlation coefficients of 0.75 for repolarization markers and 0.57 for depolarization markers. Distinct spatial lead patterns were also identified according to the occluded artery and the presence of a prior infarction. I find these data interesting and original. I would suggest only adding a figure of real-life ECGs in order to strengthen the study's conclusions and main findings.

We thank the Reviewer for this helpful suggestion. We agree that adding representative ECG examples improves the clarity of the *Results* section and helps illustrate the main findings of the study.

In response to this comment, we have added a new figure to the revised manuscript (Figure 8), included in the *Results* section under the subsection *Relationship Between ECG Changes, History of Prior MI and Ischemia Extent / Severity*. The figure presents illustrative averaged ECG heartbeats during ischemia progression in two representative patients, one without prior MI and one with prior MI. Accompanying text has also been added to explain the figure and its relevance to the observed differences in ST-segment and QRS-complex changes.

Specifically, the following text has been added to the revised manuscript:

Fig 8 shows averaged heartbeats during ischemia progression for a patient without prior MI and a patient with prior MI, both presenting LAD occlusion and comparable ischemia extent (48.1% and 51.3%, respectively) and ischemia severity (19.6% and 22.2%, respectively). ECG heartbeats at the beginning of the PCI recording are shown in black, whereas progressive gray shading represents advancing occlusion, with the lightest gray shade corresponding to approximately 3.5 minutes from the start of occlusion. As shown in the figure, the patient with prior MI exhibits more pronounced ST-segment changes than the patient without prior MI, whereas QRS-complex changes are more comparable between the two patients. Overall, this example highlights the steeper relationship between ECG repolarization changes and both ischemia extent and severity in patients with prior MI.

Reviewer #2:

We are grateful to the Reviewer for the revision and for the helpful and constructive comments, which we addressed as follows.

The sample size is relatively small, particularly for subgroup and correlation analyses. The limited number of LAD cases and patients with prior MI raises concerns about statistical power and the subgroup findings.

We acknowledge the Reviewer's concern regarding the relatively small sample size, particularly for sub-

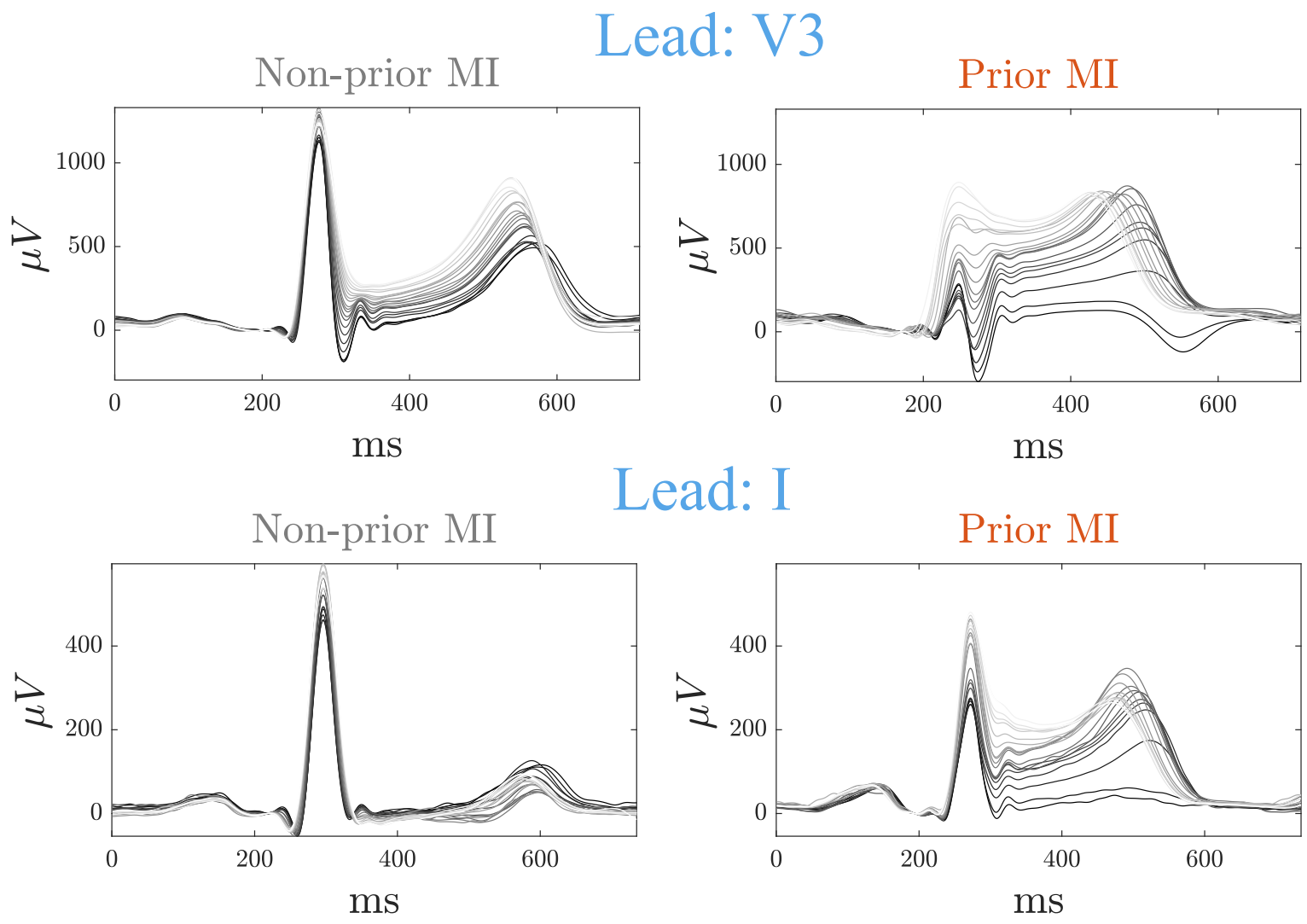

Figure 8: **ECG heartbeats during ischemia progression for a patient without prior MI (left) and a patient with prior MI (right), both with LAD occlusion, shown in leads V3 (top) and I (bottom).** Black indicates the beginning of the occlusion, and progressively lighter gray shades indicate ischemia progression, with the lightest gray corresponding to approximately 3.5 minutes from the start of the occlusion.

group and correlation analysis. Specifically, our study included 34 LAD cases and 34 patients with prior MI from the total study population ($N = 102$). Additionally, the correlation analyses with the ischemic burden were performed within the specific subset of 35 patients who underwent myocardial scintigraphy.

Nevertheless, the availability of this specific type of ECG recordings is inherently limited. The dataset analyzed in this manuscript was collected in the early 2000s and obtained from a unique human model involving approximately 4.5 minutes of controlled ischemia induced during PCI. In current clinical practice, balloon occlusion times are typically much shorter, usually around 1 minute. This is supported by clinical data showing median balloon inflation or total occlusion times of approximately 60 s [1]. Therefore, prolonged occlusions of approximately 4-5 minutes, as represented in this database, may no longer be feasible in contemporary settings [2].

To address this limitation and clarify the patient distribution across all sub-analyses, we have added clinical and demographic baseline characteristics of the study population as Table 1 (shown below) at the beginning of the *Results* section in the *Temporal Dynamics and Magnitude of Ischemia-Induced ECG Changes* subsection.

Additionally, we have added the following statement to the *Limitations and future work* section:

The sample size is relatively small, reflecting the limited availability of this specific type of ECG recordings, which was collected in the early 2000s. Nevertheless, this database represents a unique human model of nearly 5 minutes of controlled ischemia induced during PCI. Given the dataset size, particularly for specific subcohorts (LAD artery occlusions ($n = 34$, 33.3%) and patients with prior MI ($n = 34$, 33.3%), the statistical findings should be interpreted with appropriate caution.

Table 1: **Baseline demographic and clinical characteristics of the study population.**

Parameters		No.	Sex (Female)	Age	Occlusion time (s)	Prior MI (yes)	Extent %	Severity %
All		102	38.2% (39)	60 (52.7 - 69.2)	4.9 (3.3 - 5.0)	33.3% (34)	17.5 (7.8 - 30.0)%	5.5 (1.9 - 12.3)%
Occluded artery	LAD	34	44.1% (15)	62 (53 - 70)	3.9 (3 - 5)	26.5% (9)	47.0 (37.9 - 48.9)	20.9 (17.6 - 22.7)
	LCx	21	42.9% (9)	66 (52 - 75)	5 (4.8 - 5.0)	42.9% (9)	21 (9.8 - 27.0)	7.1 (3 - 9.5)
	RCA	47	31.9% (15)	58.5 (65.7 - 53)	5 (5.0 - 4)	34.0% (16)	12.1 (17.6-5)	3.4 (5.8-1.1)
Sex	F	39	100% (39)	63 (53 - 70)	4.8 (3.3 - 5)	23.1% (9)	17.9 (15.6 - 30.2)	6.8 (4.9 - 11.5)
	M	63	0% (0)	60 (51 - 67)	5 (3.4 - 5.0)	39.7% (25)	13.6 (7.8 - 29.6)	4.5 (1.8 - 12.7)
Prior MI	Yes	34	26.4% (9)	58 (52 - 67)	4.9 (3.8 - 5.0)	100%(34)	24.6 (8.4 - 29.2)	8.1 (1.9 - 12.3)
	No	68	44.1% (30)	62 (52.5 - 70)	4.9 (3.1 - 5)	0% (0)	16.5 (7.2 - 32.8)	5.3 (1.9 - 11.9)
Extent percentage	Yes	35	37.1% (13)	63 (53 - 73.5)	4.9 (4.8 - 5)	31.4% (11)	17.5 (7.8 - 30.0)	5.5 (1.9 - 12.3)
	No	67	38.8% (26)	59.5 (52.2 - 67)	4.9 (3 - 5)	34.3% (23)	- - -	- - -
Age	>60 yrs	49	42.9% (21)	70 (65 - 75)	5 (3.0 - 5.0)	24.5% (12)	21 (4.6 - 33.9)	7.1 (1.1 - 14.7)
	<60 yrs	51	35.3% (18)	53 (48 - 56.5)	4.9 (3.9 - 5)	41.2% (21)	17.4 (8.4 - 30.0)	4.9 (2.1 - 12.3)

Baseline characteristics of the study cohort during the PCI procedure. Values are reported as median (IQR). Extent and severity percentages showed statistically significant differences among the occluded artery groups.

The methodology for ECG processing and the definition of ECG markers require clearer and more concise description to ensure reproducibility.

Following the Reviewer's comment, we have thoroughly revised the *Materials and methods* section by expanding the information provided across all subsections. In particular, we added further details on the preprocessing, alignment and averaging procedures applied to the ECG recordings, and provided more comprehensive definitions of the ECG markers to improve clarity and reproducibility.

The subsection entitled *ECG Processing* has been revised as follows:

ECG preprocessing consisted of band-pass filtering between 0.5 and 40 Hz, QRS detection using a wavelet-based delineation algorithm [3], and baseline-wander reduction by beat-to-beat cubic spline interpolation, using the fiducial points minus 80 ms as baseline reference points [4]. After preprocessing, ECG signals were segmented into individual heartbeats according to the previously detected QRS fiducial points. Beats were temporally aligned using either the R or S wave, depending on which wave exhibited the largest amplitude during the recording. Cross-correlation analysis was then used to retain only those beats whose similarity to a template beat exceeded a predefined threshold, as described below. The selected beats were subsequently averaged to reduce noise and improve the robustness of ECG feature measurements. Ensemble averaging was applied to CR recordings, whereas exponential averaging was used for PCIR recordings [5, 6].
For ensemble averaging in CR recordings, each heartbeat was compared with the median beat computed from the entire recording, and only beats with a correlation coefficient greater than 0.9 were retained. The accepted beats were then averaged within non-overlapping 10-second segments to obtain representative beats for each segment for the CR ECG characterization.
For PCIR, heartbeats were first grouped into 10-second windows. Within each window, a median beat was computed, and beats in that time window with a correlation coefficient greater than 0.9 with the median

beat were selected. Exponential averaging was then applied to the selected beats to track dynamic ECG changes during coronary occlusion, as follows:

$$\overline{x}_i(n) = (1-\alpha)\,\overline{x}_{i-1}(n) + \alpha\, x_i(n), \tag{1}$$

where $\alpha = 0.25$, i *denotes the beat index,* $x_i(n)$, $n \in \{0, \ldots, N-1\}$ *represents the current beat, and* $\overline{x}_i(n)$ *denotes the updated averaged beat. For the PCIR ECG characterization, beats were extracted from the final exponentially averaged signal at 10-second intervals.*

The first paragraph of the subsection entitled *ECG Markers* was revised as follows:

The averaged beats obtained from the CR and PCIR recordings were delineated using a wavelet-based algorithm [7] to identify the onsets, peaks, and ends of the ECG waveforms. Based on these delineation points, the following ECG markers were extracted: QRS width (QRS_w*), measured from QRS onset to QRS end; QRS peak-to-peak amplitude (*QRS_{am}*), measured as the difference between the maximum and minimum amplitudes within the QRS complex; QRS area (*QRS_{ar}*), calculated as the integral of the absolute value of the voltage deflections over the duration of the QRS complex; T-wave peak-to-peak amplitude (*T_{am}*), measured as the difference between the maximum and minimum amplitudes within the T wave; T-wave area (*T_{ar}*), calculated as the integral of the absolute value of the voltage deflections over the T wave duration; ST-segment deviation (*ST_{dev}*), measured as the mean value of the segment from the J point to the J point + 50 ms, with the J point calculated as in [8]; ST area (*ST_{ar}*), calculated as the integral of the absolute value of the voltage deflections over the ST segment; and the ratio between the T-wave and QRS amplitudes (T/QRS).*

The final paragraphs of the subsection entitled *ECG Markers* were revised as follows:

To characterize the overall patient-level response of each ECG marker ι*, the* 90th *percentile of* R_ι *across the 12 leads was computed for each patient at each time point* t_j*. These values were then summarized by reporting the median across patients. Group comparisons at each time point were performed using the Wilcoxon rank-sum test or the Kruskal-Wallis test, with statistical significance set at* $p < 0.05$*.*

Finally, to assess the relationship between ischemic burden and ECG changes, Pearson correlation coefficients ρ *were calculated between scintigraphically derived measures of extent or severity and* $|R_\iota(t_j)|$*, with statistical significance set at* $p < 0.05$*. Correlations were evaluated at two clinically relevant time points:* $t_j = 30$ *s and* $t_j = 150$ *s after the onset of occlusion, representing the early and advanced phases of ischemia during the PCI procedure.*

All data analysis was performed using MATLAB R2022b [9].

The manuscript lacks tables in general, including a baseline characteristics table summarizing the study population and relevant subgroup comparisons.

Following the Reviewer's suggestion, we added a baseline characteristics table (shown below) as Table 1 at the beginning of the *Results* section in the *Temporal Dynamics and Magnitude of Ischemia-Induced ECG Changes* subsection. This table summarizes the main demographic and clinical characteristics of the study population, including sex distribution, age, prior myocardial infarction status, occlusion time during the PCI procedure, and scintigraphic measures of ischemic extent and severity. In addition, subgroup comparisons are provided according to occluded artery, sex, prior myocardial infarction status, ischemic extent and severity, and age group.

Table 1: **Baseline demographic and clinical characteristics of the study population.**

Parameters		No.	Sex (Female)	Age	Occlusion time (s)	Prior MI (yes)	Extent %	Severity %
All		102	38.2% (39)	60 (52.7 - 69.2)	4.9 (3.3 - 5.0)	33.3% (34)	17.5 (7.8 - 30.0)%	5.5 (1.9 - 12.3)%
Occluded artery	LAD	34	44.1% (15)	62 (53 - 70)	3.9 (3 - 5)	26.5% (9)	47.0 (37.9 - 48.9)	20.9 (17.6 - 22.7)
	LCx	21	42.9% (9)	66 (52 - 75)	5 (4.8 - 5.0)	42.9% (9)	21 (9.8 - 27.0)	7.1 (3 - 9.5)
	RCA	47	31.9% (15)	58.5 (65.7 - 53)	5 (5.0 - 4)	34.0% (16)	12.1 (17.6-5)	3.4 (5.8-1.1)
Sex	F	39	100% (39)	63 (53 - 70)	4.8 (3.3 - 5)	23.1% (9)	17.9 (15.6 - 30.2)	6.8 (4.9 - 11.5)
	M	63	0% (0)	60 (51 - 67)	5 (3.4 - 5.0)	39.7% (25)	13.6 (7.8 - 29.6)	4.5 (1.8 - 12.7)
Prior MI	Yes	34	26.4% (9)	58 (52 - 67)	4.9 (3.8 - 5.0)	100%(34)	24.6 (8.4 - 29.2)	8.1 (1.9 - 12.3)
	No	68	44.1% (30)	62 (52.5 - 70)	4.9 (3.1 - 5)	0% (0)	16.5 (7.2 - 32.8)	5.3 (1.9 - 11.9)
Extent percentage	Yes	35	37.1% (13)	63 (53 - 73.5)	4.9 (4.8 - 5)	31.4% (11)	17.5 (7.8 - 30.0)	5.5 (1.9 - 12.3)
	No	67	38.8% (26)	59.5 (52.2 - 67)	4.9 (3 - 5)	34.3% (23)	- - -	- - -
Age	>60 yrs	49	42.9% (21)	70 (65 - 75)	5 (3.0 - 5.0)	24.5% (12)	21 (4.6 - 33.9)	7.1 (1.1 - 14.7)
	<60 yrs	51	35.3% (18)	53 (48 - 56.5)	4.9 (3.9 - 5)	41.2% (21)	17.4 (8.4 - 30.0)	4.9 (2.1 - 12.3)

Baseline characteristics of the study cohort during the PCI procedure. Values are reported as median (IQR). Extent and severity percentages showed statistically significant differences among the occluded artery groups.

The Results section would benefit from more comprehensive descriptive statistics. Statistical reporting is incomplete. P-values and 95% confidence intervals should be reported for all main comparisons and correlation analyses.

Following the Reviewer's comment, p-values and 95% confidence intervals were incorporated into all main comparisons and correlation analyses. Statistical significance was added to the plots and described in the corresponding figure captions within the *Results* section. These modifications were applied to Figures 4, 5, 6, 9, and 10, as well as to corresponding figures in the *Supporting Information* section (Figures S2, S3, S5, S6, S7, and S8). Additionally, correlation coefficients (ρ) and their statistical significance were included in Figure 7, with equivalent updates made to the corresponding supplementary figure (Figure S4).

The manuscript relies heavily on fold-change metrics, but their clinical interpretation is not clearly explained.

We agree with the Reviewer and have clarified the clinical relevance and interpretation of our fold-change metrics. In this study, expressing ECG marker variations as fold changes (relative to baseline) is clinically important, as it mitigates inter-patient variability in baseline ECG amplitudes and durations, enabling standardized quantification of the acute ischemic effect. The clinical interpretation of our fold-change metrics has been further expanded in the *Materials and methods* section and in the *Discussion* section.

Specifically, the following paragraph was added to the subsection entitled *ECG Markers* in the *Materials and methods* section:

Clinically, $R_\iota(t_j)$ represents the magnitude of ischemia-induced changes relative to baseline variability, such that larger absolute values indicate deviations that are more likely attributable to ischemia rather than physiological fluctuations.

The following paragraph was added at the beginning of the subsection entitled *Temporal Evolution of ECG Changes* in the *Discussion* section:

Relative changes in ECG markers during ischemia were quantified using the normalized index $R_\iota(t_j)$*, which tracks acute ischemic deviations relative to each patient's own physiological fluctuations. Unlike traditional absolute ECG measurements, which are inherently confounded by high inter-individual baseline variability,* $R_\iota(t_j)$ *unmasks subtle temporal dynamics. Using this relative measure, we observed that ventricular repolarization markers responded more rapidly to ischemia than depolarization markers during the initial minutes of coronary occlusion. This observation aligns with the electrophysiological mechanisms underlying acute ischemia.*

Also, the following paragraph was added at the end of the subsection entitled *Temporal Evolution of ECG Changes* in the *Discussion* section:

From a clinical perspective, these findings demonstrate that repolarization markers such as ST_{dev}*,* ST_{ar}*, and* T/QRS *act as early indicators of acute myocardial ischemia, reflecting early electrical alterations following coronary occlusion. This rapid response is particularly relevant in time-critical settings, such as emergency or pre-hospital care, where earlier detection may facilitate timely triage and intervention. In contrast, the more gradual evolution of depolarization markers suggests a complementary role in monitoring ischemic evolution. Together, these observations support the potential value of combining early-responsive repolarization indices with slower depolarization markers to improve both detection and temporal characterization of ischemic events.*

The interpretation of prior MI effects may be overstated, given that this subgroup represents a relatively small portion of the cohort.

We thank the Reviewer for this insightful comment. Among the 102 patients included in the study, 34 patients (33.3%) had prior MI. Additionally, of the 102 patients, 35 patients (34.3%) underwent scintigraphic assessment from which 11 had a history of prior MI. To further examine the potential influence of prior MI on the relationship between ischemic characteristics and ECG changes, we have added a new supplementary figure (Figure S4) in the revised manuscript. While Figure 7 presents the relationship between ischemic extent and changes in ST_{dev}, T_{am}, and QRS_w, Figure S4 provides the analogous analysis for ischemic severity.

In response to the Reviewer's concern, and given that the effect of prior MI on ischemia-induced ECG changes appears limited, we have moderated our interpretation accordingly. Specifically, the slopes of the fitted linear models at 150 s were 2.93, 0.96, and 0.50 for ST_{dev}, T_{am}, and QRS_w, respectively, in patients with prior MI, compared with 0.74, 0.56, and 0.43 in patients without prior MI. These findings suggest modest differences between groups. The corresponding discussion has been revised in the subsection *Role of Prior MI and Ischemic Extent and Severity in Acute Ischemic ECG Dynamics* of the *Discussion* section, first, second and fourth paragraphs.

Specifically, the following texts were added to that subsection of the *Discussion* section:

Our results reveal a modest trend toward more pronounced and sustained ischemia-induced ECG responses in patients with a prior MI compared to those without, even when ischemic extent and severity are similar.

...

Collectively, these findings indicate that while the pre-existing myocardial substrate may modulate the electrical expression of acute ischemia, this modulating effect remains modest.

From a clinical perspective, these observations further suggest that relative changes in ECG markers may remain informative even in patients with pre-existing ECG abnormalities due to prior MI. This is particularly relevant in clinical practice, where baseline ECG alterations often complicate diagnosis. Moreover, the modest amplification of ischemia-induced responses in these patients may reflect increased electrical vulnerability, which could have implications for risk stratification and closer monitoring during

acute ischemic episodes.

Additionally, we performed univariable linear regression analyses to quantify the contribution of prior MI to ischemia-induced ECG changes. In these models, the relative change in each ECG marker measured 150 seconds after occlusion onset was used as the dependent variable, with prior MI as the sole independent variable. The proportion of explained variance was minimal, with $R^2 = 0.002$ for ST_{dev}, $R^2 = 0.033$ for T_{am}, and $R^2 = 0.005$ for QRS_w.

For comparison, we evaluated additional univariable models including other covariates (age, sex, occluded artery location, and ischemic extent and severity). Among these, only ischemic extent and severity explained a substantial fraction of the variance in ECG changes. At 150 seconds after occlusion, the R^2 values for ischemic extent/severity were 0.337/0.512 for ST_{dev}, 0.141/0.174 for T_{am}, and 0.255/0.340 for QRS_w.

We further performed multivariable regression analyses including prior MI together with ischemic extent (or severity). The corresponding R^2 values were 0.339 (extent) and 0.513 (severity) for ST_{dev}, 0.175 and 0.215 for T_{am}, and 0.260 and 0.349 for QRS_w, indicating marginal improvement compared with univariable models based on ischemic characteristics alone, with more accentuated effects found for T_{am}.

Finally, we explored combinations of predictors and found that ischemic extent and severity together provided the most complementary information. A model including both variables yielded R^2 values of 0.711 for ST_{dev}, 0.188 for T_{am}, and 0.395 for QRS_w. Adding prior MI as an additional covariate increased the explained variance slightly, with a more marked impact for T_{am}, resulting in R^2 values of 0.713, 0.236, and 0.412, respectively.

Taken together, these analyses indicate that, although prior MI explains only a small proportion of the variability in ischemia-induced ECG changes compared with scintigraphic measures, its presence appears to modestly reinforce the strength of the association between ECG changes and ischemic extent or severity.

The above results were incorporated into the manuscript and appear as the final paragraph of subsection *Relationship Between ECG Changes, History of Prior MI and Ischemia Extent / Severity* within the *Results* section. The added paragraph reads as follows:

To further quantify the relative contribution of prior MI to ischemia-induced ECG changes, we performed additional univariable and multivariable regression analyses. When considering prior MI as the sole independent variable, the proportion of explained variance for each ECG marker at 150 s of occlusion was negligible, with $R^2 = 0.002$, 0.033, *and* 0.005 *for* ST_{dev}, T_{am}, *and* QRS_w, *respectively. In contrast, ischemia extent and severity accounted for a substantially larger fraction of the variability, with* R^2 *values of 0.337/0.512, 0.141/0.174, and 0.255/0.340, respectively. Including prior MI together with ischemic extent or severity in multivariable models resulted in marginal improvements in explained variance, with more accentuated effects for* T_{am}. *Notably, the combination of ischemia extent and severity yielded the highest explanatory power (*$R^2 = 0.711$, 0.188, *and* 0.395*), while adding prior MI as an additional covariate produced a slight increase, most evident for* T_{am} *where* $R^2 = 0.236$. *These results indicate that, although prior MI has a limited direct contribution to the overall variability in ECG changes, its presence appears to modestly enhance the strength of the relationship between ECG changes and ischemic extent or severity.*

The reliance on baseline ECG recordings limits real-world applicability, as such data are not always available in clinical settings.

We thank the Reviewer for this important observation. We acknowledge that reliance on a baseline ECG acquired prior to coronary occlusion may limit direct applicability in some real-world clinical scenarios, where previous recordings are not consistently available. Our study was conducted in a controlled setting (cardiac catheterization laboratory), which enabled a precise characterization of ischemia-induced ECG changes relative to an individual baseline.

Nevertheless, we believe that this limitation does not necessarily preclude future clinical translation. In

practical settings, an alternative reference could be obtained from the earliest available ECG recording, such as those acquired in the ambulance or upon arrival at the emergency department. These recordings could serve as a reference ECG, allowing subsequent ECG changes to be quantified using the same framework proposed in this study, although they may not always represent a pre-ischemic baseline.

Importantly, the methodology presented here is inherently well suited for applications involving continuous or sequential ECG monitoring. For instance, it could be applied during patient transport, in chest pain units, intensive care settings, or during in-hospital evaluation of acute coronary syndromes, where temporal ECG evolution is routinely available. In addition, the approach may be particularly valuable in controlled ischemia-inducing scenarios, such as stress testing, where a baseline recording is naturally available and changes can be tracked over time.

By focusing on intra-patient relative variations (fold-changes) rather than absolute population-wide thresholds, our framework effectively mitigates confounding inter-individual differences, maximizing sensitivity to acute ischemic changes. We have incorporated these considerations into the revised manuscript (*Limitations and future work* section). Overall, we believe that the proposed methodology provides a solid foundation for the development of patient-specific ECG monitoring tools, with promising potential for real-time ischemia tracking. Future studies in more heterogeneous and less controlled clinical environments will be key to further validating and extending these applications.

Following the Reviewer's comment, the following paragraph was added to the *Limitations and future work* section in the updated manuscript.

A limitation of this study is the reliance on a baseline ECG acquired prior to coronary occlusion, which may not always be available in routine clinical practice. Although this requirement could restrict direct applicability in some settings, an alternative baseline may be obtained from the earliest ECG recorded during patient transport or admission or, alternatively, from previous ECG acquisitions available from the patient. However, these recordings may not necessarily reflect a true pre-ischemic state. Moreover, the proposed approach is particularly well suited for continuous monitoring scenarios, where sequential ECG recordings allow tracking of intra-individual changes over time. While inter-individual variability limits the use of these markers for absolute comparisons across patients, the relative stability of these metrics within the individuals supports their potential for personalized ischemia monitoring in real-world clinical environments.

The Discussion should further elaborate on the clinical implications and potential applications of the findings.

We agree with the Reviewer and have revised the *Discussion* section to provide clinical implications and potential application of the study findings as follows:

- In the subsection entitled *Temporal Evolution of ECG Changes* the following paragraph was added: *From a clinical perspective, these findings suggest that repolarization markers such as* ST_{dev}, ST_{ar}, *and* T/QRS *may serve as sensitive early indicators of acute myocardial ischemia, capturing electrical alterations within seconds of coronary occlusion. This rapid response is particularly relevant in time-critical settings, such as emergency or pre-hospital care, where earlier detection may facilitate timely triage and intervention. In contrast, the more gradual evolution of depolarization markers suggests a complementary role in monitoring ischemic evolution. Together, these observations support the potential value of combining early-responsive repolarization indices with slower depolarization markers to improve both detection and temporal characterization of ischemic events.*

- In the subsection entitled *Role of Sex and Age in Ischemic ECG Dynamics*, within the text related to sex, the following paragraph was added: *These results demonstrate that fixed, absolute ECG thresholds contribute to underestimating ischemic severity in female patients due to the lower magnitude of change and earlier plateau of their repolarization markers. This supports current guideline recommendations for sex-specific ST-elevation criteria and suggests that incorporating relative or dynamic ECG measures could further improve diagnostic sensitivity. Increased awareness of these differences may therefore help reduce disparities in the diagnosis and management of acute coronary syndromes in female patients.*

- In the subsection entitled *Role of Sex and Age in Ischemic ECG Dynamics*, within the text related to age, the following paragraph was added: *These age-related differences in temporal ECG behavior*

suggest that clinical interpretation should consider not only the magnitude but also the rate of change of ischemia-induced alterations. The more abrupt but transient responses observed in older patients may increase the likelihood of missed diagnoses if ECG recordings are not obtained early, whereas the more sustained progression in younger patients may provide a wider diagnostic window. This highlights the potential value of serial ECG monitoring and time-dependent analysis of ECG markers, particularly in older individuals in whom ischemic changes may be less persistent.

- In the subsection entitled *Role of Occluded Artery in Acute Ischemic ECG Dynamics* the following paragraph was added: *Clinically, our results highlight that quantitative ECG markers may not only aid in detecting ischemia but also provide information about its anatomical origin. The more pronounced and spatially localized changes observed in LAD occlusions suggest that the analyzed ECG indices could assist in identifying the likely affected coronary artery in acute settings. Conversely, the less distinctive patterns associated with LCx occlusion reinforce known limitations of the standard 12-lead ECG and support the use of additional posterior leads or complementary diagnostic tools when LCx involvement is suspected.*
- In the subsection entitled *Role of Prior MI and Ischemic Extent and Severity in Acute Ischemic ECG Dynamics* the following paragraph was added: *From a clinical perspective, these observations further suggest that relative changes in ECG markers may remain informative even in patients with pre-existing ECG abnormalities due to prior MI. This is particularly relevant in clinical practice, where baseline ECG alterations often complicate diagnosis. Moreover, the modest amplification of ischemia-induced responses in these patients may reflect increased electrical vulnerability, which could have implications for risk stratification and closer monitoring during acute ischemic episodes.*
- In the *Limitations and future work* section, the text describing future work was revised to include potential clinical applications. Specifically, the following paragraph was added: *Beyond these methodological considerations, the present findings also point toward several potential avenues for clinical translation. The use of normalized indices such as R_ι, which account for baseline variability, may enable more individualized ECG interpretation compared to conventional fixed thresholds. In addition, the integration of multiple ECG markers capturing both depolarization and repolarization processes could enhance diagnostic performance, particularly in early or ambiguous presentations of acute coronary syndromes. More broadly, combining ECG-derived features with clinical variables such as age, sex, and prior MI may support the development of predictive models for improved decision-making. Further prospective studies are warranted to assess the diagnostic utility and incremental value of these approaches in real-world settings.*

The authors should consider multivariable regression analysis to determine whether prior MI remains an independent predictor of ECG changes after adjusting for potential confounders (e.g., ischemia extent/severity, age, sex, occlusion site, angiographic data).

Following the Reviewer's comment, we performed univariable linear regression analyses to quantify the contribution of prior MI to ischemia-induced ECG changes. In these models, the relative change in each ECG marker measured 150 seconds after occlusion onset was used as the dependent variable, with prior MI as the sole independent variable. The proportion of explained variance was minimal, with $R^2 = 0.002$ for ST_{dev}, $R^2 = 0.033$ for T_{am}, and $R^2 = 0.005$ for QRS_w.

For comparison, we evaluated additional univariable models including other covariates (age, sex, occluded artery location, and ischemic extent and severity). Among these, only ischemic extent and severity explained a substantial fraction of the variance in ECG changes. At 150 seconds after occlusion, the R^2 values for ischemic extent/severity were 0.337/0.512 for ST_{dev}, 0.141/0.174 for T_{am}, and 0.255/0.340 for QRS_w.

We further performed multivariable regression analyses including prior MI together with ischemic extent (or severity). The corresponding R^2 values were 0.339 (extent) and 0.513 (severity) for ST_{dev}, 0.175 and 0.215 for T_{am}, and 0.260 and 0.349 for QRS_w, indicating only marginal improvement compared with univariable models based on ischemic characteristics alone.

Finally, we explored combinations of predictors and found that ischemic extent and severity together provided the most complementary information. A model including both variables yielded R^2 values of

0.711 for ST_{dev}, 0.188 for T_{am}, and 0.395 for QRS_w. Adding prior MI as an additional covariate increased the explained variance slightly, with a more marked impact for T_{am}, resulting in R^2 values of 0.713, 0.236, and 0.412, respectively.

Taken together, these analyses indicate that, although prior MI explains only a small proportion of the variability in ischemia-induced ECG changes compared with scintigraphic measures, its presence appears to modestly reinforce the strength of the association between ECG changes and ischemic extent or severity.

The above results were incorporate into the manuscript and appear as the final paragraph of subsection *Relationship Between ECG Changes, History of Prior MI and Ischemia Extent / Severity within the Results section. The added paragraph reads as follows:*

To further quantify the relative contribution of prior MI to ischemia-induced ECG changes, we performed additional univariable and multivariable regression analyses. When considering prior MI as the sole independent variable, the proportion of explained variance for each ECG marker at 150 s of occlusion was negligible, with $R^2 = 0.002$, 0.033, *and* 0.005 *for* ST_{dev}, T_{am}, *and* QRS_w, *respectively. In contrast, ischemia extent and severity accounted for a substantially larger fraction of the variability, with* R^2 *values of 0.337/0.512, 0.141/0.174, and 0.255/0.340, respectively. Including prior MI together with ischemic extent or severity in multivariable models resulted in only marginal improvements in explained variance. Notably, the combination of ischemia extent and severity yielded the highest explanatory power (*$R^2 = 0.711$, 0.188, *and* 0.395*), while adding prior MI as an additional covariate produced only a slight increase, most evident for* T_{am} *where* $R^2 = 0.236$. *These results indicate that, although prior MI has a limited direct contribution to the overall variability in ECG changes, its presence appears to modestly enhance the strength of the relationship between ECG changes and ischemic extent or severity.*

---

6. PLOS authors have the option to publish the peer review history of their article (what does this mean?). If published, this will include your full peer review and any attached files.

If you choose "no", your identity will remain anonymous but your review may still be made public.

Do you want your identity to be public for this peer review? For information about this choice, including consent withdrawal, please see our Privacy Policy.

Reviewer #1: Yes: Afendoulis Dimitrios

Reviewer #2: No

[NOTE: If reviewer comments were submitted as an attachment file, they will be attached to this email and accessible via the submission site. Please log into your account, locate the manuscript record, and check for the action link "View Attachments". If this link does not appear, there are no attachment files.]

To ensure your figures meet our technical requirements, please review our figure guidelines: `https://journals.plos.org/plosone/s/figures` **You may also use PLOS's free figure tool, NAAS, to help you prepare publication quality figures:** `https://journals.plos.org/plosone/s/figures#loc-tools-for-figure-preparation`. **NAAS will assess whether your figures meet our technical requirements by comparing each figure against our figure specifications.**

References

[1] Rhee TM, Lee JM, Shin ES, Hwang D, Park J, Jeon KH, et al. Impact of optimized procedure-related factors in drug-eluting balloon angioplasty for treatment of in-stent restenosis. JACC: Cardiovascular Interventions. 2018;11(10):969-78. doi:10.1016/j.jcin.2018.02.002.

[2] Gurgoglione FL, Gattuso D, Greco A, Donelli D, Niccoli G, Cortese B. Angiographic and clinical impact of balloon inflation time in percutaneous coronary interventions with sirolimus-coated balloon: A subanalysis of the EASTBOURNE study. Cardiovascular Revascularization Medicine. 2025;73:70-5. doi:10.1016/j.carrev.2024.07.021.

[3] Martínez JP, Almeida R, Olmos S, Rocha AP, Laguna P. A wavelet-based ECG delineator: evaluation on standard databases. IEEE Transactions on Biomedical Engineering. 2004;51(4):570-81. doi:10.1109/TBME.2003.821031.

[4] Meyer C, Keiser H. Electrocardiogram baseline noise estimation and removal using cubic splines and state-space computation techniques. Computers and Biomedical Research. 1977;10(5):459-70. doi:10.1016/0010-4809(77)90021-0.

[5] Pueyo E, Sörnmo L, Laguna P. QRS slopes for detection and characterization of myocardial ischemia. IEEE Transactions on Biomedical Engineering. 2008;55(2):468-77. doi:10.1109/TBME.2007.902228.

[6] Pahlm O, Sörnmo L. Data processing of exercise ECG's. IEEE Transactions on Biomedical Engineering. 1987;BME-34(2):158-65. doi:10.1109/TBME.1987.326040.

[7] Martínez JP, Pahlm O, Ringborn M, Warren S, Laguna P, Sörnmo L. The STAFF III database: ECGs recorded during acutely induced myocardial ischemia. In: 2017 Computing in Cardiology (CinC). vol. 44. IEEE; 2017. p. 1-4. doi:10.22489/CinC.2017.266-133.

[8] Mincholé A, Jager F, Laguna P. Discrimination between ischemic and artifactual ST segment events in Holter recordings. Biomedical Signal Processing and Control. 2010;5(1):21-31. doi:10.1016/j.bspc.2009.09.001.

[9] The MathWorks Inc . MATLAB version: 9.13.0 (R2022b). Natick, Massachusetts, United States: The MathWorks Inc.; 2022. Available from: `https://www.mathworks.com`.
