# Supplementary material for: Characterizing acute ischemia via ECG: A study of severity, extent, and spatial variability in myocardial infarction and non-infarction groups
Source: PLoS One. 2026 Oct 29;21(10):e0361220. doi: 10.1371/journal.pone.0361220 (PMC13719058; doi:10.1371/journal.pone.0361220)
Supplement: Supplementary file 3 [file pone.0361220.s004.pdf]

September 21st, 2026

Resubmission of manuscript: "Characterizing acute ischemia via ECG: a study of severity, extent, and spatial variability in myocardial infarction and non-infarction groups" with reference PONE-D-26-08831

Jimena Rodríguez-Carbó
University of Zaragoza
Zaragoza, Spain
jimena.rodriguez@unizar.es

Editor in Chief
PLOS One

Dear Editor,

We would like to thank you for the opportunity to revise our manuscript, "Characterizing acute ischemia via ECG: a study of severity, extent, and spatial variability in myocardial infarction and non-infarction groups". We appreciate the review and the constructive suggestions provided by the Reviewers. We believe the manuscript has been strengthened by incorporating these modifications.

Journal Requirements:
Reviewers' comments:
Reviewer's Responses to Questions

Comments to the Author

1. If the authors have adequately addressed your comments raised in a previous round of review and you feel that this manuscript is now acceptable for publication, you may indicate that here to bypass the "Comments to the Author" section, enter your conflict of interest statement in the "Confidential to Editor" section, and submit your "Accept" recommendation.

Reviewer #3: (No Response)
Reviewer #4: All comments have been addressed

---

2. Is the manuscript technically sound, and do the data support the conclusions?

The manuscript must describe a technically sound piece of scientific research with data that supports the conclusions. Experiments must have been conducted rigorously, with appropriate controls, replication, and sample sizes. The conclusions must be drawn appropriately based on the data presented.

Reviewer #3: No
Reviewer #4: Yes

---

3. Has the statistical analysis been performed appropriately and rigorously?

Reviewer #3: No
Reviewer #4: Yes

---

4. Have the authors made all data underlying the findings in their manuscript fully available?

The PLOS Data policy requires authors to make all data underlying the findings described in their manuscript fully available without restriction, with rare exception (please refer to the Data Availability Statement in the manuscript PDF file). The data should be provided as part of the manuscript or its supporting information, or deposited to a public repository. For example, in addition to summary statistics, the data points behind means, medians and variance measures should be available. If there are restrictions on publicly sharing data—e.g. participant privacy or use of data from a third party—those must be specified.

Reviewer #3: No
Reviewer #4: Yes

---

5. Is the manuscript presented in an intelligible fashion and written in standard English?

PLOS ONE does not copyedit accepted manuscripts, so the language in submitted articles must be clear, correct, and unambiguous. Any typographical or grammatical errors should be corrected at revision, so please note any specific errors here.

Reviewer #3: Yes

Reviewer #4: Yes

---

6. Review Comments to the Author

Please use the space provided to explain your answers to the questions above. You may also include additional comments for the author, including concerns about dual publication, research ethics, or publication ethics. (Please upload your review as an attachment if it exceeds 20,000 characters)

Reviewer #3:

We are grateful to the Reviewer for the revision and for the helpful and constructive comments, which we addressed as follows.

Perform multivariable analyses to identify independent predictors of ECG changes.

Following the Reviewer's comment, we performed multivariable linear regression analysis to quantify the independent contribution of sex, age, prior MI, and ischemia extent and severity to ischemia-induced ECG changes.

The updated paragraph in the *Results* section, subsection *Relationship Between ECG Changes, History of Prior MI and Ischemia Extent / Severity*, that presents the results of this multivariable analysis reads as follows:

Additional statistical analyses were performed to quantify the relative contribution of prior MI, age, sex, and ischemia extent and severity to ischemia-induced ECG changes using univariable and multivariable regression models. When considering prior MI as the sole independent variable, the proportion of explained variance for each ECG marker at 150 s of occlusion was negligible, with $R^2 = 0.003$, 0.034, *and* 0.005 *for* ST_{dev}, T_{am}, *and* QRS_w, *respectively. In contrast, ischemia extent and severity accounted for a substantially larger fraction of the variability, with* R^2 *values of 0.337/0.512, 0.141/0.175, and 0.255/0.340, respectively. Including prior MI together with ischemic extent or severity in multivariable models resulted in marginal improvements in explained variance, with more accentuated effects for* T_{am}*. Notably, the combination of ischemia extent and severity yielded higher explanatory power (*$R^2 = 0.711$, 0.188, *and* 0.395*), while adding prior MI as an additional covariate produced a slight increase, most evident for* T_{am}, *with* $R^2 = 0.236$. *These results indicate that, although prior MI has a limited direct contribution to the overall variability in ECG changes, its presence appears to modestly enhance the strength of the relationship between ECG changes and ischemic extent and severity. Including sex, age, prior MI, and ischemia extent and severity as independent variables in a multivariable model increased* R^2 *values to* 0.727, 0.303, *and* 0.466 *for* ST_{dev}, T_{am}, *and* QRS_w, *respectively. In this fully adjusted model, ischemia extent and severity were independently associated with* ST_{dev}, *whereas ischemia severity was independently associated with* QRS_w. *Age, sex, and prior MI were not independently associated with any of the evaluated ECG markers. For* T_{am}, *the overall multivariable model did not reach statistical significance.*

Address multiple-comparison testing.

We thank the Reviewer for this suggestion. To address the correction for multiple hypothesis testing, p-values were adjusted using the Benjamini–Hochberg method. Specifically, Figures 4, 5, 6, 9, and 10, as well as the corresponding supplementary figures, were updated to reflect the correction for multiple comparisons. After correction, significant comparisons remained in Figures 9 and 10, which are indicated directly in the corresponding panels. In addition, effect sizes were reported to complement the adjusted p-values and quantify the magnitude of the observed differences. The statistical methods were expanded to describe this correction as follows:

To characterize the overall patient-level response of each ECG marker ι, *the* 90th *percentile of* R_ι *across*

the 12 leads was computed for each patient at each time point t_j. These values were then summarized by reporting the median across patients. To account for multiple hypothesis testing, p-values were adjusted using the Benjamini–Hochberg procedure, applied within each family of related comparisons. Because statistical significance is influenced by sample size, effect sizes were additionally reported to quantify the magnitude of the observed differences. Specifically, the effect size r was reported for the Wilcoxon rank-sum test, whereas η_H^2 was reported for the Kruskal–Wallis test [1]. Following the guidelines in [1], the Wilcoxon rank-sum effect size r ranges from -1 to 1 and was interpreted based on its absolute value as small ($0.1 \leq |r| < 0.3$), medium ($0.3 \leq |r| < 0.5$), or large ($|r| \geq 0.5$). Similarly, the Kruskal–Wallis effect size η_H^2 ranges from 0 to 1 and was classified as small ($0.01 \leq \eta_H^2 < 0.06$), medium ($0.06 \leq \eta_H^2 < 0.14$), or large ($\eta_H^2 \geq 0.14$), with larger values indicating greater effect magnitude [1].

Clarify how prior MI was diagnosed.

We acknowledge the Reviewer's comment. We have clarified how prior MI was defined in the *Materials and methods* section under the *Study Population* subsection:

Prior MI was identified using ECG-based screening criteria applied to a standard ECG recorded at hospital admission [2]. These screening criteria were based on a subset of the Selvester QRS scoring system [3], which assigns weighted points according to the duration and amplitude of individual QRS deflections across the ECG leads. The screening criteria were: (1) a Q-wave duration ≥ 30 ms in lead aVF; (2) an R-wave duration ≤ 10 ms and R-wave amplitude ≤ 0.1 mV in lead V2; and (3) an R-wave duration ≥ 40 ms in lead V1. The remaining criteria of the full 32-point Selvester QRS scoring system [4] were considered non-screening criteria. Patients were classified as having no prior MI if none of the three screening criteria was present in any available cardiac cycle and the total score from the non-screening criteria was ≤ 2 points. Prior MI was defined by the presence of at least one screening criterion in all available cardiac cycles or by a total score ≥ 4 points from the non-screening criteria, with at least two of these points derived from leads other than V4–V6. Patients who met neither the no-MI nor the MI criteria were classified as borderline for previous MI and excluded from further analysis [5].

Provide more detailed statistical methods and effect sizes.

Following the Reviewer's comment, we have expanded the *Materials and methods* section, particularly the subsection describing the statistical methods, and added effect-size estimates to the relevant group-comparison figures (Figures 9 and 10 and Figures S5, S6, S7, and S8 in the Supplementary Material). The updated text reads as follows.

To characterize the overall patient-level response of each ECG marker ι, the 90th percentile of R_ι across the 12 leads was computed for each patient at each time point t_j. These values were then summarized by reporting the median across patients. To account for multiple hypothesis testing, p-values were adjusted using the Benjamini–Hochberg procedure, applied within each family of related comparisons. Because statistical significance is influenced by sample size, effect sizes were additionally reported to quantify the magnitude of the observed differences. Specifically, the effect size r was reported for the Wilcoxon rank-sum test, whereas η_H^2 was reported for the Kruskal–Wallis test [1]. Following the guidelines in [1], the Wilcoxon rank-sum effect size r ranges from -1 to 1 and was interpreted based on its absolute value as small ($0.1 \leq |r| < 0.3$), medium ($0.3 \leq |r| < 0.5$), or large ($|r| \geq 0.5$). Similarly, the Kruskal–Wallis effect size η_H^2 ranges from 0 to 1 and was classified as small ($0.01 \leq \eta_H^2 < 0.06$), medium ($0.06 \leq \eta_H^2 < 0.14$), or large ($\eta_H^2 \geq 0.14$), with larger values indicating greater effect magnitude [1].

Additionally, we have added the following phrases in the *Results* section, subsection *Relationship Between ECG Changes and Site of Occlusion*, to comment on the effect size of the group comparison and the pairwise comparisons:

To examine whether the site of coronary occlusion influences the magnitude and spatial distribution of ischemia-induced ECG alterations, Fig 9 presents selected ECG markers (ST_{dev}, T_{am}, and QRS_w) measured 150 seconds after occlusion for patients with LAD, RCA, and LCx artery occlusions. The left panels show the box plots of R_ι for each marker, computed as the 90th percentile across the 12 ECG leads at 150 s post-occlusion for each artery group. Repolarization markers (ST_{dev} and T_{am}) exhibited the highest median $|R_\iota|$ values in patients with LAD occlusion, followed by RCA, with the lowest values

observed in the LCx group. For example, in ST_{dev}, patients with LAD occlusion reached median values of 68-fold compared to baseline, 29-fold in patients with RCA occlusion, and 21-fold for patients with LCx occlusion. Statistically significant differences in $|R_\iota|$ were observed across artery groups for ST_{dev}, with a medium overall effect size ($\eta_H^2 = 0.08$). The significant LAD versus LCx comparison also showed a medium effect size ($|r| = 0.40$). Differences in depolarization markers were less pronounced, although QRS_w showed slightly higher median values in the LCx group.

The right panels of Fig 9 illustrate the spatial distribution of $|R_\iota|$ across the 12 leads for each artery group. Distinct lead-specific patterns emerged depending on the occluded artery. LAD occlusion produced prominent changes in leads V2–V5, consistent with anterior myocardial ischemia. RCA occlusion was associated with the largest changes in leads II, III, and aVF, whereas LCx occlusion did not yield a clearly distinctive spatial pattern.

Consistent with these ECG findings, patients with LAD occlusion exhibited the highest median ischemia extent and severity, followed by LCx and RCA occlusions (Fig 10). Significant differences were observed across artery groups for both ischemia extent and severity, with medium overall effect sizes ($\eta_H^2 = 0.09$ for both variables). Pairwise comparisons involving LAD showed small effect sizes ($|r| = 0.16$–0.17). No significant age differences were observed among the artery groups, suggesting that age was unlikely to account for these differences.

Reduce overstatement regarding clinical applicability.

We have modified the *Discussion* to address the Reviewer's comment. In particular, the following subsections have been rephrased: *Temporal Evolution of ECG Changes*, *Role of Sex and Age in Ischemic ECG Dynamics*, *Role of Occluded Artery in Acute Ischemic ECG Dynamics*, and *Role of Prior MI and Ischemic Extent and Severity in Acute Ischemic ECG Dynamics.* The revised text addressing the potential clinical implications now reads as follows:

- In the subsection entitled *Temporal Evolution of ECG Changes*, the following paragraph has been rephrased:

  From a clinical perspective, these findings suggest that repolarization markers such as ST_{dev}, ST_{ar}, and T/QRS may serve as sensitive early indicators of acute myocardial ischemia, capturing electrical alterations within seconds of coronary occlusion. However, whether this rapid response translates into improved ischemia detection in clinical practice, particularly in emergency or pre-hospital settings, remains unknown. In contrast, the more gradual evolution of depolarization markers suggests a complementary role in tracking the progression of ischemia over time. Together, these observations indicate that combining early-responsive repolarization indices with more slowly evolving depolarization markers may provide complementary information for the detection and temporal characterization of ischemic events. Nevertheless, the clinical utility of such a combined approach remains to be studied in prospective clinical studies.

- In the subsection entitled *Role of Sex and Age in Ischemic ECG Dynamics*, within the text related to sex, the following paragraph has been rephrased:

  These results suggest that fixed, absolute ECG thresholds may not fully capture sex-related differences in the magnitude and temporal evolution of ischemia-induced ECG changes. This observation is consistent with current guideline recommendations for sex-specific ST-elevation criteria and suggests that incorporating relative or dynamic ECG measures warrants further investigation as a means of improving diagnostic sensitivity. Validation in larger and more diverse cohorts is required to determine whether these differences have implications for the diagnosis and management of acute coronary syndromes in female patients.

- In the subsection entitled *Role of Occluded Artery in Acute Ischemic ECG Dynamics*, the following paragraph was modified:

  In terms of their potential clinical relevance, our results indicate that quantitative ECG markers capture differences related to the anatomical location of coronary occlusion. The more pronounced and spatially localized changes observed in LAD occlusions suggest that the analyzed ECG indices may contain information relevant to identifying the affected coronary territory. Conversely, the less distinctive patterns associated with LCx occlusion are consistent with known limitations of the standard 12-lead ECG and support the potential value of additional posterior leads or complementary diagnostic tools when LCx involvement is suspected.

- In the subsection entitled *Role of Prior MI and Ischemic Extent and Severity in Acute Ischemic ECG Dynamics*, the following paragraph was modified:

  Nevertheless, these observations suggest that relative changes in ECG markers may remain informative even in patients with pre-existing ECG abnormalities due to prior MI. This may be particularly relevant for characterizing ischemia-induced ECG changes in the presence of an altered baseline ECG. Moreover, the modest amplification of ischemia-induced responses observed in these patients may reflect increased electrical vulnerability, potentially providing additional insight into the electrophysiological consequences of prior MI. However, further validation in larger patient populations and in other clinical settings is needed before the clinical significance and potential utility of these findings can be established.

Include additional numerical summary tables.

Following the Reviewer's suggestion, we have added a summary table in the *Results* section, specifically in the subsection *Relationship Between ECG Changes, History of Prior MI and Ischemia Extent / Severity.* Table 1 summarizes the patients' maximum changes in ST_{dev}, T_{am}, and QRS_w during the first four minutes of occlusion and quantified in both absolute $|\Delta_\iota|$ and relative $|R_\iota|$ terms. Numerical values show median (IQR) values separately for patients with and without prior MI.

Table 1: **Median (IQR) values of the patients' maximum relative $|R_\iota|$ and absolute $|\Delta_\iota|$ changes in ST_{dev}, T_{am}, and QRS_w during the first four minutes of occlusion, stratified by prior MI status.**

$\|R_\iota\|$	Non-prior MI (N = 68)	Prior MI (N = 34)
ST_{dev}	39.7915 [19.47 , 73.67]	49.83 [15.20 , 98.92]
T_{am}	23.29 [15.85 , 42.71]	30.67 [17.31 , 53.56]
QRS_w	29.63 [14.11 , 50.68]	31.11 [11.83 , 99.53]
$\|\Delta_\iota\|$	Non-prior MI	Prior MI
ST_{dev} [μV]	155.79 [72.65 , 304.67]	168.19 [95.49 , 245.53]
T_{am} [μV]	212.84 [145.83 , 299.52]	212.21 [131.15 , 361.85]
QRS_w [ms]	31.54 [21.51 , 45.73]	38.94 [15.30 , 67.33]

Expand the limitations to address generalizability, small subgroup sizes, and absence of external validation.
Clarify whether medications or procedural variables could have influenced ECG responses.

We acknowledge the Reviewer's comments on the limitations of the study. We have expanded the *Limitations and future work* section to more explicitly address the generalizability of the findings, the limited size of some subgroups, the absence of external validation, and the potential influence of pharmacological and procedural factors. The new text reads:

Several limitations should be considered when interpreting the present findings:

- *A limitation of this study is the reliance on a baseline ECG acquired prior to coronary occlusion, which may not always be available in routine clinical practice. Although alternative baseline recordings may be obtained from the earliest ECG recorded during patient transport or hospital admission, or from previous ECG examinations, such recordings may not fully represent a true pre-ischemic state. The present approach may therefore be most applicable in settings where serial ECG recordings are available, allowing assessment of intra-individual changes over time. While inter-individual variability limits direct comparison of marker values across patients, the relatively stable behavior of these markers within individuals suggests potential utility for longitudinal ischemia assessment. However, the feasibility and clinical value of this approach require further evaluation in real-world settings.*

- *The sample size is relatively small, reflecting the limited availability of this specific type of ECG recordings, which were collected in the early 2000s. Nevertheless, this database represents a unique human model of nearly five minutes of controlled ischemia induced during PCI. Given the small*

size of certain subcohorts, including patients with LAD artery occlusions ($n = 34$, 33.3%) and those with prior MI ($n = 34$, 33.3%), as well as the subgroup for whom information on ischemia extent and severity was available, the statistical findings, in particular those derived from subgroup and multivariate analyses, should be interpreted with appropriate caution.

- *Occlusions occurred at varying anatomical levels (proximal, mid, or distal), and data on the extent of ischemia were unavailable for a substantial proportion of patients. These procedural differences may have influenced the magnitude and temporal evolution of the ECG response and could not be fully accounted for in the present analysis.*

- *Potential confounders, including left ventricular hypertrophy, left anterior fascicular block, long QT syndrome, other comorbidities, and pharmacological therapy, may have influenced the observed results. Because the investigated markers were evaluated relative to each patient's own baseline ECG, these factors were present in both recordings, and their impact may therefore have been partially mitigated. Nevertheless, it remains possible that comorbidities and medication influenced the electrophysiological response to acute ischemia. Further studies are needed to assess the effects of these factors on the proposed ECG markers.*

- *Information regarding medical therapy in patients with a prior MI, as well as the timing of the previous infarction relative to the ischemic event, was not available, limiting a more detailed assessment of their potential influence on the ECG response.*

- *As noted above, the recordings used in this study originate from a highly specific human model of acute ischemia induced during PCI, which enabled controlled assessment of ischemia-related ECG changes. However, the controlled nature of this model may limit the generalizability of the findings to routine clinical practice. External validation in a comparable independent cohort is currently challenging, as datasets including similarly prolonged, controlled coronary occlusions during PCI are not readily available under current clinical practice. Further studies in contemporary real-world clinical settings and larger patient cohorts are therefore needed to confirm the robustness, generalizability, and applicability of these observations.*

- *Although recordings were acquired in the supine position, in which the standard 12-lead ECG may represent cardiac orientation differently than in the standing position, this factor is unlikely to account for the artery-specific spatial patterns observed in the present study.*

- *Finally, the documented location of the prior MI was less precise than that of the acute ischemic event, limiting detailed interpretation.*

Beyond these methodological considerations, the present findings also point toward several potential avenues for clinical translation. The use of normalized indices such as R_ι, which account for baseline variability, may facilitate the assessment of intra-individual ECG changes compared with conventional fixed thresholds. In addition, the integration of multiple ECG markers capturing both depolarization and repolarization processes may provide complementary information on the temporal evolution of acute ischemia. More broadly, combining ECG-derived features with clinical variables such as age, sex, and prior MI may support the development of multivariable models for improved characterization of acute ischemia. Further prospective studies in larger patient cohorts and across diverse clinical settings are warranted to determine the diagnostic utility and incremental value of these approaches in real-world practice.

Reviewer #4:

We are grateful to the Reviewer for the revision and for the helpful and constructive comments.

The authors have satisfactorily addressed all comments raised by both reviewers in the previous round. In particular:
The addition of Table 1 (baseline characteristics) and Figure 8 (representative ECG examples) substantially improves clarity and reproducibility. The revised Materials and Methods section now provides sufficiently detailed and reproducible descriptions of ECG preprocessing and marker definitions. The newly performed univariable and multivariable regression

analyses appropriately and transparently characterize the limited independent contribution of prior MI to ischemia-induced ECG changes, and the authors have moderated their interpretation accordingly. Statistical reporting (p-values, 95% CIs, correlation coefficients) has been incorporated throughout the Results section as requested. The Discussion section now adequately addresses clinical implications and limitations, including the reliance on baseline ECG recordings and the constraints of sample size.

---

7. PLOS authors have the option to publish the peer review history of their article (what does this mean?). If published, this will include your full peer review and any attached files.

If you choose “no”, your identity will remain anonymous but your review may still be made public.

Do you want your identity to be public for this peer review? For information about this choice, including consent withdrawal, please see our Privacy Policy.

Reviewer #3: Yes: Isha Abdullah Ali

Reviewer #4: No

References

[1] Fiel Peres F. Effect sizes for nonparametric tests. Biochemia Medica. 2026;36(1):5-16.

[2] Martínez JP, Pahlm O, Ringborn M, Warren S, Laguna P, Sörnmo L. The STAFF III database: ECGs recorded during acutely induced myocardial ischemia. In: 2017 Computing in Cardiology (CinC). vol. 44. IEEE; 2017. p. 1-4. doi:10.22489/CinC.2017.266-133.

[3] Selvester RH, Wagner GS, Hindman NB. The Selvester QRS scoring system for estimating myocardial infarct size: the development and application of the system. Archives of Internal Medicine. 1985;145(10):1877-81.

[4] Anderson WD, Wagner NB, Lee KL, White RD, Yuschak J, Behar VS, et al. Evaluation of a QRS scoring system for estimating myocardial infarct size. VI: Identification of screening criteria for non-acute myocardial infarcts. The American Journal of Cardiology. 1988;61(10):729-33.

[5] Pettersson J, Carro E, Edenbrandt L, Maynard C, Pahlm O, Ringborn M, et al. Spatial, individual, and temporal variation of the high-frequency QRS amplitudes in the 12 standard electrocardiographic leads. American Heart Journal. 2000;139(2):352-8.
