# Supplementary material for: Characterizing acute ischemia via ECG: A study of severity, extent, and spatial variability in myocardial infarction and non-infarction groups
Source: PLoS One. 2026 Oct 29;21(10):e0361220. doi: 10.1371/journal.pone.0361220 (PMC13719058; doi:10.1371/journal.pone.0361220)
Supplement: Peer review history

# Peer review: Decision Letter 0

**DOI:** 10.1371/journal.pone.0361220.r001

**Authors:** Redoy Ranjan

11 May 2026

Dear Dr. Rodríguez-Carbó,

Thank you for submitting your manuscript to PLOS ONE. After careful consideration, we feel that it has merit but does not fully meet PLOS ONE’s publication criteria as it currently stands. Therefore, we invite you to submit a revised version of the manuscript that addresses the points raised during the review process.

Please submit your revised manuscript by Jun 25 2026 11:59PM. If you will need more time than this to complete your revisions, please reply to this message or contact the journal office at plosone@plos.org. When you're ready to submit your revision, log on to https://www.editorialmanager.com/pone/ and select the 'Submissions Needing Revision' folder to locate your manuscript file.

- A letter that responds to each point raised by the academic editor and reviewer(s). You should upload this letter as a separate file labeled 'Response to Reviewers'.
- A marked-up copy of your manuscript that highlights changes made to the original version. You should upload this as a separate file labeled 'Revised Manuscript with Track Changes'.
- An unmarked version of your revised paper without tracked changes. You should upload this as a separate file labeled 'Manuscript'.

If you would like to make changes to your financial disclosure, please include your updated statement in your cover letter. Guidelines for resubmitting your figure files are available below the reviewer comments at the end of this letter.

If applicable, we recommend that you deposit your laboratory protocols in protocols.io to enhance the reproducibility of your results. Protocols.io assigns your protocol its own identifier (DOI) so that it can be cited independently in the future. For instructions see: https://journals.plos.org/plosone/s/submission-guidelines#loc-laboratory-protocols. Additionally, PLOS ONE offers an option for publishing peer-reviewed Lab Protocol articles, which describe protocols hosted on protocols.io. Read more information on sharing protocols at https://plos.org/protocols?utm_medium=editorial-email&utm_source=authorletters&utm_campaign=protocols.

As the corresponding author, your ORCID iD is verified in the submission system and will appear in the published article. PLOS supports the use of ORCID, and we encourage all coauthors to register for an ORCID iD and use it as well. Please encourage your coauthors to verify their ORCID iD within the submission system before final acceptance, as unverified ORCID iDs will not appear in the published article. *Only*  the individual author can complete the verification step; PLOS staff *cannot*  verify ORCID iDs on behalf of authors.

We look forward to receiving your revised manuscript.

Kind regards,

Redoy Ranjan, MS (CV&TS), Ch.M. (Edin), PhD

Academic Editor

PLOS One

**Journal Requirements:**

1. When submitting your revision, we need you to address these additional requirements.

Please ensure that your manuscript meets PLOS ONE's style requirements, including those for file naming. The PLOS ONE style templates can be found at

https://journals.plos.org/plosone/s/file?id=wjVg/PLOSOne_formatting_sample_main_body.pdf and

https://journals.plos.org/plosone/s/file?id=ba62/PLOSOne_formatting_sample_title_authors_affiliations.pdf

2. Please note that PLOS One has specific guidelines on code sharing for submissions in which author-generated code underpins the findings in the manuscript. In these cases, we expect all author-generated code to be made available without restrictions upon publication of the work. Please review our guidelines at https://journals.plos.org/plosone/s/materials-and-software-sharing#loc-sharing-code and ensure that your code is shared in a way that follows best practice and facilitates reproducibility and reuse.

3. We note that the grant information you provided in the ‘Funding Information’ and ‘Financial Disclosure’ sections do not match.

When you resubmit, please ensure that you provide the correct grant numbers for the awards you received for your study in the ‘Funding Information’ section.

4. Thank you for stating in your Funding Statement:

This work was supported by projects PID2022-140556OB-I00 (https://www.aei.gob.es/convocatorias/buscador-convocatorias/proyectos-generacion-conocimiento-2022/convocatoria) awarded to EP, CNS2022-135899 (https://www.aei.gob.es/convocatorias/buscador-convocatorias/consolidacion-investigadora-2022) awarded to AM, and TED2021-130459B-I00 funded by MCIN/AEI/10.13039/501100011033 and "ERDF A way of making Europe" (https://www.aei.gob.es/convocatorias/buscador-convocatorias/proyectos-estrategicos-orientados-transicion-ecologica) awarded AM, EP, and JRC. The study was also supported by fellowship RYC2019-027420-I funded by the Ramón y Cajal Program (https://www.aei.gob.es/convocatorias/buscador-convocatorias/ayudas-contratos-ramon-cajal-ryc-2019) awarded to AM, by the BSICoS group T39\_23R funded by the Aragón Government and FEDER 2014-2020 "Building Europe from Aragon" (https://www.aragon.es/fondos-europeos/feder-fondo-de-desarrollo-regional/2014-2020) awarded to AM, EP, and JRC, and by the PhD fellowship "Ayudas para iberoamericanos y ecuatoguineanos en Estudios de Doctorado ‐ Curso 2023/2024" funded by the University of Zaragoza and Banco Santander (https://internacional.unizar.es/convocatoria-de-ayudas-para-iberoamericanos-y-ecuatoguineanos-en-estudios-de-doctorado-2324) awarded to JRC.

Please provide an amended statement that declares *all* the funding or sources of support (whether external or internal to your organization) received during this study, as detailed online in our guide for authors at http://journals.plos.org/plosone/s/submit-now.  Please also include the statement “There was no additional external funding received for this study.” in your updated Funding Statement.

Please include your amended Funding Statement within your cover letter. We will change the online submission form on your behalf.

5. Thank you for stating the following financial disclosure:

This work was supported by projects PID2022-140556OB-I00 (https://www.aei.gob.es/convocatorias/buscador-convocatorias/proyectos-generacion-conocimiento-2022/convocatoria) awarded to EP, CNS2022-135899 (https://www.aei.gob.es/convocatorias/buscador-convocatorias/consolidacion-investigadora-2022) awarded to AM, and TED2021-130459B-I00 funded by MCIN/AEI/10.13039/501100011033 and "ERDF A way of making Europe" (https://www.aei.gob.es/convocatorias/buscador-convocatorias/proyectos-estrategicos-orientados-transicion-ecologica) awarded AM, EP, and JRC. The study was also supported by fellowship RYC2019-027420-I funded by the Ramón y Cajal Program (https://www.aei.gob.es/convocatorias/buscador-convocatorias/ayudas-contratos-ramon-cajal-ryc-2019) awarded to AM, by the BSICoS group T39\_23R funded by the Aragón Government and FEDER 2014-2020 "Building Europe from Aragon" (https://www.aragon.es/fondos-europeos/feder-fondo-de-desarrollo-regional/2014-2020) awarded to AM, EP, and JRC, and by the PhD fellowship "Ayudas para iberoamericanos y ecuatoguineanos en Estudios de Doctorado ‐ Curso 2023/2024" funded by the University of Zaragoza and Banco Santander (https://internacional.unizar.es/convocatoria-de-ayudas-para-iberoamericanos-y-ecuatoguineanos-en-estudios-de-doctorado-2324) awarded to JRC.

Please state what role the funders took in the study.  If the funders had no role, please state: "The funders had no role in study design, data collection and analysis, decision to publish, or preparation of the manuscript."

If this statement is not correct you must amend it as needed.

Please include this amended Role of Funder statement in your cover letter; we will change the online submission form on your behalf.

6. We noted in your submission details that a portion of your manuscript may have been presented or published elsewhere. [Preliminary results of this study were presented in the Computing in Cardiology Conference in 2023 and published in the conference's proceedings. These results and images were improved by using different metrics and further analyses were computed to create the study presented here. The results presented in the conference were uploaded in the Attached Files.] Please clarify whether this [conference proceeding or publication] was peer-reviewed and formally published. If this work was previously peer-reviewed and published, in the cover letter please provide the reason that this work does not constitute dual publication and should be included in the current manuscript.

7. If the reviewer comments include a recommendation to cite specific previously published works, please review and evaluate these publications to determine whether they are relevant and should be cited. There is no requirement to cite these works unless the editor has indicated otherwise.

[Note: HTML markup is below. Please do not edit.]

Reviewers' comments:

**Comments to the Author**

1. Is the manuscript technically sound, and do the data support the conclusions?

Reviewer #1: Yes

Reviewer #2: No

**********

2. Has the statistical analysis been performed appropriately and rigorously?

Reviewer #1: Yes

Reviewer #2: No

**********

3. Have the authors made all data underlying the findings in their manuscript fully available?

The PLOS Data policy

Reviewer #1: Yes

Reviewer #2: No

**********

4. Is the manuscript presented in an intelligible fashion and written in standard English?

Reviewer #1: Yes

Reviewer #2: No

**********

**Reviewer #1:**  Quite an interesting manuscript regarding ischemia induced ECG changes beyond the ST-T deviation.

According to the authors, beyond ST-segment deviation, multiple ECG markers evolve

over time during coronary artery occlusion. Repolarization features showed earlier and

more pronounced alterations than depolarization features, reaching approximately

37-fold and 23-fold changes at 4 minutes of occlusion compared with baseline,

respectively. Males showed faster, sustained ST increases (up to ∼47-fold vs ∼30-fold in

females), while older patients had abrupt early ST peaks (∼16-fold at ∼2 min after

occlusion), whereas younger patients exhibited a more gradual increase. Moreover,

patients with a prior infarction showed a stronger association between ECG changes

and both the extent and severity of ischemia, reaching maximum correlation coefficients

of 0.75 for repolarization markers and 0.57 for depolarization markers. Distinct spatial

lead patterns were also identified according to the occluded artery and the presence of a

prior infarction.

I find these data interesting and original. I would suggest only adding a figure of real -life ECGs in order to strengthen the study's conclusions and main findings.

**Reviewer #2:**  the sample size is relatively small, particularly for subgroup and correlation analyses. The limited number of LAD cases and patients with prior MI raises concerns about statistical power and the subgroup findings.

The methodology for ECG processing and the definition of ECG markers require clearer and more concise description to ensure reproducibility.

The manuscript lacks tables in general, including a baseline characteristics table summarizing the study population and relevant subgroup comparisons.

The Results section would benefit from more comprehensive descriptive statistics.

Statistical reporting is incomplete. P-values and 95% confidence intervals should be reported for all main comparisons and correlation analyses.

The manuscript relies heavily on fold-change metrics, but their clinical interpretation is not clearly explained.

The interpretation of prior MI effects may be overstated, given that this subgroup represents a relatively small portion of the cohort.

The reliance on baseline ECG recordings limits real-world applicability, as such data are not always available in clinical settings.

The Discussion should further elaborate on the clinical implications and potential applications of the findings.

The authors should consider multivariable regression analysis to determine whether prior MI remains an independent predictor of ECG changes after adjusting for potential confounders (e.g., ischemia extent/severity, age, sex, occlusion site, angiographic data).

**********

what does this mean?). If published, this will include your full peer review and any attached files.

If you choose “no”, your identity will remain anonymous but your review may still be made public.

**Do you want your identity to be public for this peer review?**  For information about this choice, including consent withdrawal, please see our Privacy Policy

Reviewer #1: **Yes:**  Afendoulis Dimitrios

Reviewer #2: No

**********

[NOTE: If reviewer comments were submitted as an attachment file, they will be attached to this email and accessible via the submission site. Please log into your account, locate the manuscript record, and check for the action link "View Attachments". If this link does not appear, there are no attachment files.]

To ensure your figures meet our technical requirements, please review our figure guidelines: https://journals.plos.org/plosone/s/figures

You may also use PLOS’s free figure tool, NAAS, to help you prepare publication quality figures: https://journals.plos.org/plosone/s/figures#loc-tools-for-figure-preparation.

NAAS will assess whether your figures meet our technical requirements by comparing each figure against our figure specifications.

# Author comment: Author response to Decision Letter 1

**DOI:** 10.1371/journal.pone.0361220.r002

25 Jun 2026

Dear Editor,

We would like to thank you for the opportunity to revise our manuscript, “Characterizing acute ischemia via ECG: a study of severity, extent, and spatial variability in myocardial infarction and non-infarction groups”. We appreciate the review and the constructive suggestions provided by the Reviewers. We believe the manuscript has been strengthened by incorporating these modifications.

Journal Requirements:

1. When submitting your revision, we need you to address these additional require-ments. Please ensure that your manuscript meets PLOS ONE’s style requirements, in-cluding those for file naming. The PLOS ONE style templates can be found at https://journals.plos.org/plosone/s/file?id=wjVg/PLOSOne_formatting_sample_main_body.pdf and https://journals.plos.org/plosone/s/fileid=ba62/PLOSOne_formatting_sample_title_author s_affiliations.pdf.

We made the corresponding modifications to meet the requirements. We modified the names of the figures within the text. References were moved to a section after the Acknowledgments.

2. Please note that PLOS One has specific guidelines on code sharing for submissions in which author-generated code underpins the findings in the manuscript. In these cases, we expect all author-generated code to be made available without restrictions upon publication of the work. Please review our guidelines at https://journals.plos.org/plosone/s/materi als-and-software-sharing#loc-sharing-code and ensure that your code is shared in a way that follows best practice and facilitates reproducibility and reuse.

We made the software used to compute the values of the R index, which is used to present the results along the text, available in Github https://github.com/JRDZC/R_index.git.

3. We note that the grant information you provided in the ‘Funding Information’ and ‘Financial Disclosure’ sections do not match. When you resubmit, please ensure that you provide the correct grant numbers for the awards you received for your study in the ‘Funding Information’ section.

Thank you for pointing this out. We corrected the grant information to ensure consistency between the ‘Funding Information’ and ‘Financial Disclosure’ sections.

4. Thank you for stating in your Funding Statement:

This work was supported by projects PID2022-140556OB-I00 and PID2023-148975OB-I00 (https://www.aei.gob.es/convocatorias/buscador-convocatorias/proyectos-generacion-conocimie nto-2022/convocatoria) awarded to EP and AM, CNS2022-135899 (https://www.aei.gob.es/con vocatorias/buscador-convocatorias/consolidacion-investigadora-2022) awarded to AM, and TED2021-130459B-I00 funded by MCIN/AEI/10.13039/501100011033 and ”ERDF A way of making Europe” (https://www.aei.gob.es/convocatorias/buscador-convocatorias/proyectos-estra tegicos-orientados-transicion-ecologica) awarded to AM, EP, and JRC. The study was also supported by fellowship RYC2019-027420-I funded by the Ram´on y Cajal Program (https://www.ae i.gob.es/convocatorias/buscador-convocatorias/ayudas-contratos-ramon-cajal-ryc-2019) awarded to AM, by the BSICoS group T39 23R funded by the Arag´on Government and FEDER 2014-2020 “Building Europe from Aragon” (https://www.aragon.es/fondos-europeos/feder-fondo

-de-desarrollo-regional/2014-2020) awarded to AM, EP, and JRC, and by the PhD fellowship ”Ayudas para iberoamericanos y ecuatoguineanos en Estudios de Doctorado - Curso 2023/2024” funded by the University of Zaragoza and Banco Santander (https://internacional.unizar.es/convocat oria-de-ayudas-para-iberoamericanos-y-ecuatoguineanos-en-estudios-de-doctorado-2324) awarded to JRC.

Please provide an amended statement that declares *all* the funding or sources of support (whether external or internal to your organization) received during this study, as detailed online in our guide for authors at http://journals.plos.org/plosone/s/submit-now. Please also include the statement “There was no additional external funding received for this study.” in your updated Funding Statement. Please include your amended Funding Statement within your cover letter. We will change the online submission form on your behalf.

We added the statements at the end of the financial disclousure statement.

This work was supported by projects PID2022-140556OB-I00 and PID2023-148975OB-I00 (https://ww w.aei.gob.es/convocatorias/buscador-convocatorias/proyectos-generacion-conocimient o-2022/convocatoria) awarded to EP and AM, CNS2022-135899 (https://www.aei.gob.es/convo catorias/buscador-convocatorias/consolidacion-investigadora-2022) awarded to AM, and TED2021-130459B-I00 funded by MCIN/AEI/10.13039/501100011033 and ”ERDF A way of making Europe” (https://www.aei.gob.es/convocatorias/buscador-convocatorias/proyectos-estra tegicos-orientados-transicion-ecologica) awarded to AM, EP, and JRC. The study was also supported by fellowship RYC2019-027420-I funded by the Ram´on y Cajal Program (https://www.ae i.gob.es/convocatorias/buscador-convocatorias/ayudas-contratos-ramon-cajal-ryc-2019) awarded to AM, by the BSICoS group T39 23R funded by the Arag´on Government and FEDER 2014-2020 “Building Europe from Aragon” (https://www.aragon.es/fondos-europeos/feder-fondo

-de-desarrollo-regional/2014-2020) awarded to AM, EP, and JRC, and by the PhD fellowship ”Ayudas para iberoamericanos y ecuatoguineanos en Estudios de Doctorado - Curso 2023/2024” funded by the University of Zaragoza and Banco Santander (https://internacional.unizar.es/convocat oria-de-ayudas-para-iberoamericanos-y-ecuatoguineanos-en-estudios-de-doctorado-2324) awarded to JRC. There was no additional external funding received for this study. The funders had no role in study design, data collection and analysis, decision to publish, or preparation of the manuscript.

5. Thank you for stating the following financial disclosure:

This work was supported by projects PID2022-140556OB-I00 (https://www.aei.gob.es/convocatorias/buscador-convocatorias/proyectos-generacion-conocimiento-2022/convocatoria) awarded to EP, CNS2022-135899 (https://www.aei.gob.es/convocatorias/buscador-convocatorias/consolidacion-investigadora-2022) awarded to AM, and TED2021-130459B-I00 funded by MCIN/AEI/10.13039/501100011033 and "ERDF A way of making Europe" (https://www.aei.gob.es/convocatorias/buscador-convocatorias/proyectos-estrategicos-orientados-transicion-ecologica) awarded AM, EP, and JRC. The study was also supported by fellowship RYC2019-027420-I funded by the Ramón y Cajal Program (https://www.aei.gob.es/convocatorias/buscador-convocatorias/ayudas-contratos-ramon-cajal-ryc-2019) awarded to AM, by the BSICoS group T39\_23R funded by the Aragón Government and FEDER 2014-2020 "Building Europe from Aragon" (https://www.aragon.es/fondos-europeos/feder-fondo-de-desarrollo-regional/2014-2020) awarded to AM, EP, and JRC, and by the PhD fellowship "Ayudas para iberoamericanos y ecuatoguineanos en Estudios de Doctorado ‐ Curso 2023/2024" funded by the University of Zaragoza and Banco Santander (https://internacional.unizar.es/convocatoria-de-ayudas-para-iberoamericanos-y-ecuatoguineanos-en-estudios-de-doctorado-2324) awarded to JRC.

Please state what role the funders took in the study. If the funders had no role, please state: "The funders had no role in study design, data collection and analysis, decision to publish, or preparation of the manuscript."

If this statement is not correct you must amend it as needed.

Please include this amended Role of Funder statement in your cover letter; we will change the online submission form on your behalf.

We added the statement “The funders had no role in study design, data collection and analysis, decision to publish, or preparation of the manuscript.” to the Funding statement and added the amended Role of Funders’ statement in the cover letter.

6. We noted in your submission details that a portion of your manuscript may have been presented or published elsewhere. [Preliminary results of this study were presented in the Computing in Cardiology Conference in 2023 and published in the conference's proceedings. These results and images were improved by using different metrics and further analyses were computed to create the study presented here. The results presented in the conference were uploaded in the Attached Files.] Please clarify whether this [conference proceeding or publication] was peer-reviewed and formally published. If this work was previously peer-reviewed and published, in the cover letter please provide the reason that this work does not constitute dual publication and should be included in the current manuscript.

Preliminary results from this study were presented at the Computing in Cardiology Conference in 2023 and published in https://ieeexplore.ieee.org/abstract/document/10364136. The conference presentation was selected on the basis of a short abstract of approximately 300 words, and the proceedings publication did not undergo peer review. Also, it should not be considered a dual publication because the previously published results were preliminary. The present manuscript includes improved signal preprocessing, new metrics, and additional analyses, including the assessment of ECG differences in response to induced ischemia according to sex, age, and prior myocardial infarction.

7. If the reviewer comments include a recommendation to cite specific previously published works, please review and evaluate these publications to determine whether they are relevant and should be cited. There is no requirement to cite these works unless the editor has indicated otherwise.

[Note: HTML markup is below. Please do not edit.]

Reviewers' comments:

Reviewer's Responses to Questions

Comments to the Author

1. Is the manuscript technically sound, and do the data support the conclusions?

The manuscript must describe a technically sound piece of scientific research with data that supports the conclusions. Experiments must have been conducted rigorously, with appropriate controls, replication, and sample sizes. The conclusions must be drawn appropriately based on the data presented.

Reviewer #1: Yes

Reviewer #2: No

2. Has the statistical analysis been performed appropriately and rigorously?

Reviewer #1: Yes

Reviewer #2: No

3. Have the authors made all data underlying the findings in their manuscript fully available?

The PLOS Data policy requires authors to make all data underlying the findings described in their manuscript fully available without restriction, with rare exception (please refer to the Data Availability Statement in the manuscript PDF file). The data should be provided as part of the manuscript or its supporting information, or deposited to a public repository. For example, in addition to summary statistics, the data points behind means, medians and variance measures should be available. If there are restrictions on publicly sharing data—e.g. participant privacy or use of data from a third party—those must be specified.

Reviewer #1: Yes

Reviewer #2: No

4. Is the manuscript presented in an intelligible fashion and written in standard English?

PLOS ONE does not copyedit accepted manuscripts, so the language in submitted articles must be clear, correct, and unambiguous. Any typographical or grammatical errors should be corrected at revision, so please note any specific errors here.

Reviewer #1: Yes

Reviewer #2: No

5. Review Comments to the Author

Please use the space provided to explain your answers to the questions above. You may also include additional comments for the author, including concerns about dual publication, research ethics, or publication ethics. (Please upload your review as an attachment if it exceeds 20,000 characters)

--Reviewer #1:

We are grateful to the Reviewer for the revision and for the helpful and constructive comments, which we addressed as follows.

- Quite an interesting manuscript regarding ischemia induced ECG changes beyond the ST-T deviation. According to the authors, beyond ST-segment deviation, multiple ECG markers evolve over time during coronary artery occlusion. Repolarization features showed earlier and more pronounced alterations than depolarization features, reaching approximately 37-fold and 23-fold changes at 4 minutes of occlusion compared with baseline, respectively. Males showed faster, sustained ST increases (up to ∼47-fold vs ∼30-fold in females), while older patients had abrupt early ST peaks (∼16-fold at ∼2 min after occlusion), whereas younger patients exhibited a more gradual increase. Moreover, patients with a prior infarction showed a stronger association between ECG changes and both the extent and severity of ischemia, reaching maximum correlation coefficients of 0.75 for repolarization markers and 0.57 for depolarization markers. Distinct spatial lead patterns were also identified according to the occluded artery and the presence of a prior infarction. I find these data interesting and original. I would suggest only adding a figure of real -life ECGs in order to strengthen the study's conclusions and main findings.

We thank the Reviewer for this helpful suggestion. We agree that adding representative ECG examples improves the clarity of the Results section and helps illustrate the main findings of the study.

In response to this comment, we have added a new figure to the revised manuscript (Figure 8), included in the Results section under the subsection Relationship Between ECG Changes, History of Prior MI and Ischemia Extent / Severity. The figure presents illustrative averaged ECG heartbeats during ischemia progression in two representative patients, one without prior MI and one with prior MI. Accompanying text has also been added to explain the figure and its relevance to the observed differences in ST-segment and QRS-complex changes.

Specifically, the following text has been added to the revised manuscript:

Fig 8 shows averaged heartbeats during ischemia progression for a patient without prior MI and a pa-tient with prior MI, both presenting LAD occlusion and comparable ischemia extent (48.1% and 51.3%, respectively) and ischemia severity (19.6% and 22.2%, respectively). ECG heartbeats at the beginning of the PCI recording are shown in black, whereas progressive gray shading represents advancing occlusion, with the lightest gray shade corresponding to approximately 3.5 minutes from the start of occlusion. As shown in the figure, the patient with prior MI exhibits more pronounced ST-segment changes than the patient without prior MI, whereas QRS-complex changes are more comparable between the two patients. Overall, this example highlights the steeper relationship between ECG repolarization changes and both ischemia extent and severity in patients with prior MI.

-- Reviewer #2:

We are grateful to the Reviewer for the revision and for the helpful and constructive comments, which we addressed as follows.

- The sample size is relatively small, particularly for subgroup and correlation analyses. The limited number of LAD cases and patients with prior MI raises concerns about statistical power and the subgroup findings.

We acknowledge the Reviewer’s concern regarding the relatively small sample size, particularly for sub-group and correlation analysis. Specifically, our study included 34 LAD cases and 34 patients with prior MI from the total study population (N = 102). Additionally, the correlation analyses with the ischemic burden were performed within the specific subset of 35 patients who underwent myocardial scintigraphy.

Nevertheless, the availability of this specific type of ECG recordings is inherently limited. The dataset analyzed in this manuscript was collected in the early 2000s and obtained from a unique human model involving approximately 4.5 minutes of controlled ischemia induced during PCI. In current clinical prac-tice, balloon occlusion times are typically much shorter, usually around 1 minute. This is supported by clinical data showing median balloon inflation or total occlusion times of approximately 60 s [1]. Therefore, prolonged occlusions of approximately 4-5 minutes, as represented in this database, may no longer be feasible in contemporary settings [2].

To address this limitation and clarify the patient distribution across all sub-analyses, we have added clinical and demographic baseline characteristics of the study population as Table 1 (shown below) at the beginning of the Results section in the Temporal Dynamics and Magnitude of Ischemia-Induced ECG Changes subsection.

Additionally, we have added the following statement to the Limitations and future work section:

The sample size is relatively small, reflecting the limited availability of this specific type of ECG record-ings, which was collected in the early 2000s. Nevertheless, this database represents a unique human model of nearly 5 minutes of controlled ischemia induced during PCI. Given the dataset size, particularly for specific subcohorts (LAD artery occlusions (n = 34, 33.3%) and patients with prior MI (n = 34, 33.3%), the statistical findings should be interpreted with appropriate caution.

- The methodology for ECG processing and the definition of ECG markers require clearer and more concise description to ensure reproducibility.

Following the Reviewer’s comment, we have thoroughly revised the Materials and methods section by expanding the information provided across all subsections. In particular, we added further details on the preprocessing, alignment and averaging procedures applied to the ECG recordings, and provided more comprehensive definitions of the ECG markers to improve clarity and reproducibility.

The subsection entitled ECG Processing has been revised as follows:

ECG preprocessing consisted of band-pass filtering between 0.5 and 40 Hz, QRS detection using a wavelet-based delineation algorithm [3], and baseline-wander reduction by beat-to-beat cubic spline interpolation, using the fiducial points minus 80 ms as baseline reference points [4]. After preprocessing, ECG signals were segmented into individual heartbeats according to the previously detected QRS fiducial points. Beats were temporally aligned using either the R or S wave, depending on which wave exhibited the largest amplitude during the recording. Cross-correlation analysis was then used to retain only those beats whose similarity to a template beat exceeded a predefined threshold, as described below. The selected beats were subsequently averaged to reduce noise and improve the robustness of ECG feature measurements. Ensemble averaging was applied to CR recordings, whereas exponential averaging was used for PCIR recordings [5, 6].

For ensemble averaging in CR recordings, each heartbeat was compared with the median beat computed from the entire recording, and only beats with a correlation coefficient greater than 0.9 were retained. The accepted beats were then averaged within non-overlapping 10-second segments to obtain representative beats for each segment for the CR ECG characterization.

For PCIR, heartbeats were first grouped into 10-second windows. Within each window, a median beat was computed, and beats in that time window with a correlation coefficient greater than 0.9 with the median beat were selected. Exponential averaging was then applied to the selected beats to track dynamic ECG changes during coronary occlusion, as follows: xi(n) = (1 − α)xi−1(n) + α xi(n),

where α = 0.25, i denotes the beat index, xi(n), n ∈ {0, . . . , N − 1} represents the current beat, and xi(n) denotes the updated averaged beat. For the PCIR ECG characterization, beats were extracted from the final exponentially averaged signal at 10-second intervals.

The first paragraph of the subsection entitled ECG Markers was revised as follows:

The averaged beats obtained from the CR and PCIR recordings were delineated using a wavelet-based algorithm [7] to identify the onsets, peaks, and ends of the ECG waveforms. Based on these delineation points, the following ECG markers were extracted: QRS width (QRSw), measured from QRS onset to QRS end; QRS peak-to-peak amplitude (QRSam), measured as the difference between the maximum and minimum amplitudes within the QRS complex; QRS area (QRSar), calculated as the integral of the abso-lute value of the voltage deflections over the duration of the QRS complex; T-wave peak-to-peak amplitude (Tam), measured as the difference between the maximum and minimum amplitudes within the T wave; T-wave area (Tar), calculated as the integral of the absolute value of the voltage deflections over the T wave duration; ST-segment deviation (STdev), measured as the mean value of the segment from the J point to the J point + 50 ms, with the J point calculated as in [8]; ST area (STar), calculated as the integral of the absolute value of the voltage deflections over the ST segment; and the ratio between the T-wave and QRS amplitudes (T/QRS).

The final paragraphs of the subsection entitled ECG Markers were revised as follows:

To characterize the overall patient-level response of each ECG marker ι, the 90th percentile of Rι across the 12 leads was computed for each patient at each time point tj. These values were then summarized by reporting the median across patients. Group comparisons at each time point were performed using the Wilcoxon rank-sum test or the Kruskal-Wallis test, with statistical significance set at p < 0.05.

Finally, to assess the relationship between ischemic burden and ECG changes, Pearson correlation coefficients ρ were calculated between scintigraphically derived measures of extent or severity and |Rι(tj)|,

with statistical significance set at p < 0.05. Correlations were evaluated at two clinically relevant time points: tj = 30 s and tj = 150 s after the onset of occlusion, representing the early and advanced phases of ischemia during the PCI procedure.

All data analysis was performed using MATLAB R2022b [9].

- The manuscript lacks tables in general, including a baseline characteristics table summarizing the study population and relevant subgroup comparisons.

Following the Reviewer’s suggestion, we added a baseline characteristics table (shown below) as Table 1 at the beginning of the Results section in the Temporal Dynamics and Magnitude of Ischemia-Induced ECG Changes subsection. This table summarizes the main demographic and clinical characteristics of the study population, including sex distribution, age, prior myocardial infarction status, occlusion time during the PCI procedure, and scintigraphic measures of ischemic extent and severity. In addition, sub-group comparisons are provided according to occluded artery, sex, prior myocardial infarction status, ischemic extent and severity, and age group.

- The Results section would benefit from more comprehensive descriptive statistics.

Statistical reporting is incomplete. P-values and 95% confidence intervals should be reported for all main comparisons and correlation analyses.

Following the Reviewer’s comment, p-values and 95% confidence intervals were incorporated into all main comparisons and correlation analyses. Statistical significance was added to the plots and described in the corresponding figure captions within the Results section. These modifications were applied to Figures 4, 5, 6, 9, and 10, as well as to corresponding figures in the Supporting Information section (Figures S2, S3, S5, S6, S7, and S8). Additionally, correlation coefficients (ρ) and their statistical significance were in-cluded in Figure 7, with equivalent updates made to the corresponding supplementary figure (Figure S4).

- The manuscript relies heavily on fold-change metrics, but their clinical interpretation is not clearly explained.

We agree with the Reviewer and have clarified the clinical relevance and interpretation of our fold-change metrics. In this study, expressing ECG marker variations as fold changes (relative to baseline) is clinically important, as it mitigates inter-patient variability in baseline ECG amplitudes and durations, enabling standardized quantification of the acute ischemic effect. The clinical interpretation of our fold-change metrics has been further expanded in the Materials and methods section and in the Discussion section.

Specifically, the following paragraph was added to the subsection entitled ECG Markers in the Materials and methods section:

Clinically, Rι(tj) represents the magnitude of ischemia-induced changes relative to baseline variabil-ity, such that larger absolute values indicate deviations that are more likely attributable to ischemia rather than physiological fluctuations.

The following paragraph was added at the beginning of the subsection entitled Temporal Evolution of ECG Changes in the Discussion section:

Relative changes in ECG markers during ischemia were quantified using the normalized index Rι(tj), which tracks acute ischemic deviations relative to each patient’s own physiological fluctuations. Un-like traditional absolute ECG measurements, which are inherently confounded by high inter-individual baseline variability, Rι(tj) unmasks subtle temporal dynamics. Using this relative measure, we observed that ventricular repolarization markers responded more rapidly to ischemia than depolarization markers during the initial minutes of coronary occlusion. This observation aligns with the electrophysiological mechanisms underlying acute ischemia.

Also, the following paragraph was added at the end of the subsection entitled Temporal Evolution of ECG Changes in the Discussion section:

From a clinical perspective, these findings demonstrate that repolarization markers such as STdev, STar, and T/QRS act as early indicators of acute myocardial ischemia, reflecting early electrical alter-ations following coronary occlusion. This rapid response is particularly relevant in time-critical settings, such as emergency or pre-hospital care, where earlier detection may facilitate timely triage and interven-tion. In contrast, the more gradual evolution of depolarization markers suggests a complementary role in monitoring ischemic evolution. Together, these observations support the potential value of combining early-responsive repolarization indices with slower depolarization markers to improve both detection and temporal characterization of ischemic events.

- The interpretation of prior MI effects may be overstated, given that this subgroup represents a relatively small portion of the cohort.

We thank the Reviewer for this insightful comment. Among the 102 patients included in the study, 34 patients (33.3%) had prior MI. Additionally, of the 102 patients, 35 patients (34.3%) underwent scinti-graphic assessment from which 11 had a history of prior MI. To further examine the potential influence of prior MI on the relationship between ischemic characteristics and ECG changes, we have added a new supplementary figure (Figure S4) in the revised manuscript. While Figure 7 presents the relationship between ischemic extent and changes in STdev, Tam, and QRSw, Figure S4 provides the analogous anal-ysis for ischemic severity.

In response to the Reviewer’s concern, and given that the effect of prior MI on ischemia-induced ECG changes appears limited, we have moderated our interpretation accordingly. Specifically, the slopes of the fitted linear models at 150 s were 2.93, 0.96, and 0.50 for STdev, Tam, and QRSw, respectively, in patients with prior MI, compared with 0.74, 0.56, and 0.43 in patients without prior MI. These findings suggest modest differences between groups. The corresponding discussion has been revised in the sub-section Role of Prior MI and Ischemic Extent and Severity in Acute Ischemic ECG Dynamics of the Discussion section, first, second and fourth paragraphs.

Specifically, the following texts were added to that subsection of the Discussion section:

Our results reveal a modest trend toward more pronounced and sustained ischemia-induced ECG responses in patients with a prior MI compared to those without, even when ischemic extent and severity are similar.…

Collectively, these findings indicate that while the pre-existing myocardial substrate may modulate the electrical expression of acute ischemia, this modulating effect remains modest.

From a clinical perspective, these observations further suggest that relative changes in ECG markers may remain informative even in patients with pre-existing ECG abnormalities due to prior MI. This is particularly relevant in clinical practice, where baseline ECG alterations often complicate diagnosis. Moreover, the modest amplification of ischemia-induced responses in these patients may reflect increased electrical vulnerability, which could have implications for risk stratification and closer monitoring during acute ischemic episodes.

Additionally, we performed univariable linear regression analyses to quantify the contribution of prior MI to ischemia-induced ECG changes. In these models, the relative change in each ECG marker mea-sured 150 seconds after occlusion onset was used as the dependent variable, with prior MI as the sole independent variable. The proportion of explained variance was minimal, with R2 = 0.002 for STdev, R2 = 0.033 for Tam, and R2 = 0.005 for QRSw.

For comparison, we evaluated additional univariable models including other covariates (age, sex, occluded artery location, and ischemic extent and severity). Among these, only ischemic extent and severity ex-plained a substantial fraction of the variance in ECG changes. At 150 seconds after occlusion, the R2 values for ischemic extent/severity were 0.337/0.512 for STdev, 0.141/0.174 for Tam, and 0.255/0.340 for QRSw.

We further performed multivariable regression analyses including prior MI together with ischemic extent (or severity). The corresponding R2 values were 0.339 (extent) and 0.513 (severity) for STdev, 0.175 and 0.215 for Tam, and 0.260 and 0.349 for QRSw, indicating marginal improvement compared with univariable models based on ischemic characteristics alone, with more accentuated effects found for Tam. Finally, we explored combinations of predictors and found that ischemic extent and severity together provided the most complementary information. A model including both variables yielded R2 values of 0.711 for STdev, 0.188 for Tam, and 0.395 for QRSw. Adding prior MI as an additional covariate in-creased the explained variance slightly, with a more marked impact for Tam, resulting in R2 values of 0.713, 0.236, and 0.412, respectively.

Taken together, these analyses indicate that, although prior MI explains only a small proportion of the variability in ischemia-induced ECG changes compared with scintigraphic measures, its presence appears to modestly reinforce the strength of the association between ECG changes and ischemic extent or severity.

The above results were incorporated into the manuscript and appear as the final paragraph of subsection Relationship Between ECG Changes, History of Prior MI and Ischemia Extent / Severity within the Results section. The added paragraph reads as follows:

To further quantify the relative contribution of prior MI to ischemia-induced ECG changes, we per-formed additional univariable and multivariable regression analyses. When considering prior MI as the sole independent variable, the proportion of explained variance for each ECG marker at 150 s of occlusion was negligible, with R2 = 0.002, 0.033, and 0.005 for STdev, Tam, and QRSw, respectively. In contrast, ischemia extent and severity accounted for a substantially larger fraction of the variability, with R2 values of 0.337/0.512, 0.141/0.174, and 0.255/0.340, respectively. Including prior MI together with ischemic extent or severity in multivariable models resulted in marginal improvements in explained variance, with more accentuated effects for Tam. Notably, the combination of ischemia extent and severity yielded the highest explanatory power (R2 = 0.711, 0.188, and 0.395), while adding prior MI as an additional covariate produced a slight increase, most evident for Tam where R2 = 0.236. These results indicate that, although prior MI has a limited direct contribution to the overall variability in ECG changes, its presence appears to modestly enhance the strength of the relationship between ECG changes and ischemic extent or severity.

- The reliance on baseline ECG recordings limits real-world applicability, as such data are not always available in clinical settings.

We thank the Reviewer for this important observation. We acknowledge that reliance on a baseline ECG acquired prior to coronary occlusion may limit direct applicability in some real-world clinical scenarios, where previous recordings are not consistently available. Our study was conducted in a controlled setting (cardiac catheterization laboratory), which enabled a precise characterization of ischemia-induced ECG changes relative to an individual baseline.

Nevertheless, we believe that this limitation does not necessarily preclude future clinical translation. In practical settings, an alternative reference could be obtained from the earliest available ECG recording, such as those acquired in the ambulance or upon arrival at the emergency department. These record-ings could serve as a reference ECG, allowing subsequent ECG changes to be quantified using the same framework proposed in this study, although they may not always represent a pre-ischemic baseline.

Importantly, the methodology presented here is inherently well suited for applications involving contin-uous or sequential ECG monitoring. For instance, it could be applied during patient transport, in chest pain units, intensive care settings, or during in-hospital evaluation of acute coronary syndromes, where temporal ECG evolution is routinely available. In addition, the approach may be particularly valuable in controlled ischemia-inducing scenarios, such as stress testing, where a baseline recording is naturally available and changes can be tracked over time.

By focusing on intra-patient relative variations (fold-changes) rather than absolute population-wide thresholds, our framework effectively mitigates confounding inter-individual differences, maximizing sen-sitivity to acute ischemic changes. We have incorporated these considerations into the revised manuscript (Limitations and future work section). Overall, we believe that the proposed methodology provides a solid foundation for the development of patient-specific ECG monitoring tools, with promising potential for real-time ischemia tracking. Future studies in more heterogeneous and less controlled clinical envi-ronments will be key to further validating and extending these applications.

Followin the Reviewer’s comment, the following paragraph was added to the Limitations and future work section in the updated manuscript:

A limitation of this study is the reliance on a baseline ECG acquired prior to coronary occlusion, which may not always be available in routine clinical practice. Although this requirement could restrict direct applicability in some settings, an alternative baseline may be obtained from the earliest ECG recorded during patient transport or admission or, alternatively, from previous ECG acquisitions available from the patient. However, these recordings may not necessarily reflect a true pre-ischemic state. Moreover, the proposed approach is particularly well suited for continuous monitoring scenarios, where sequential ECG recordings allow tracking of intra-individual changes over time. While inter-individual variability limits the use of these markers for absolute comparisons across patients, the relative stability of these metrics within the individuals supports their potential for personalized ischemia monitoring in real-world clinical environments.

- The Discussion should further elaborate on the clinical implications and potential applications of the findings.

We agree with the Reviewer and have revised the Discussion section to provide clinical implications and potential application of the study findings as follows:

• In the subsection entitled Temporal Evolution of ECG Changes the following paragraph was added: From a clinical perspective, these findings suggest that repolarization markers such as STdev, STar, and T/QRS may serve as sensitive early indicators of acute myocardial ischemia, capturing elec-trical alterations within seconds of coronary occlusion. This rapid response is particularly relevant in time-critical settings, such as emergency or pre-hospital care, where earlier detection may fa-cilitate timely triage and intervention. In contrast, the more gradual evolution of depolarization markers suggests a complementary role in monitoring ischemic evolution. Together, these obser-vations support the potential value of combining early-responsive repolarization indices with slower depolarization markers to improve both detection and temporal characterization of ischemic events.

• In the subsection entitled Role of Sex and Age in Ischemic ECG Dynamics, within the text related to sex, the following paragraph was added: These results demonstrate that fixed, absolute ECG thresholds contribute to underestimating ischemic severity in female patients due to the lower mag-nitude of change and earlier plateau of their repolarization markers. This supports current guideline recommendations for sex-specific ST-elevation criteria and suggests that incorporating relative or dynamic ECG measures could further improve diagnostic sensitivity. Increased awareness of these differences may therefore help reduce disparities in the diagnosis and management of acute coronary syndromes in female patients.

• In the subsection entitled Role of Sex and Age in Ischemic ECG Dynamics, within the text related to age, the following paragraph was added: These age-related differences in temporal ECG behavior suggest that clinical interpretation should consider not only the magnitude but also the rate of change of ischemia-induced alterations. The more abrupt but transient responses observed in older patients may increase the likelihood of missed diagnoses if ECG recordings are not obtained early, whereas the more sustained progression in younger patients may provide a wider diagnostic window. This highlights the potential value of serial ECG monitoring and time-dependent analysis of ECG markers, particularly in older individuals in whom ischemic changes may be less persistent.

• In the subsection entitled Role of Occluded Artery in Acute Ischemic ECG Dynamics the following paragraph was added: Clinically, our results highlight that quantitative ECG markers may not only aid in detecting ischemia but also provide information about its anatomical origin. The more pro-nounced and spatially localized changes observed in LAD occlusions suggest that the analyzed ECG indices could assist in identifying the likely affected coronary artery in acute settings. Conversely, the less distinctive patterns associated with LCx occlusion reinforce known limitations of the stan-dard 12-lead ECG and support the use of additional posterior leads or complementary diagnostic tools when LCx involvement is suspected.

• In the subsection entitled Role of Prior MI and Ischemic Extent and Severity in Acute Ischemic ECG Dynamics the following paragraph was added: From a clinical perspective, these observations further suggest that relative changes in ECG markers may remain informative even in patients with pre-existing ECG abnormalities due to prior MI. This is particularly relevant in clinical practice, where baseline ECG alterations often complicate diagnosis. Moreover, the modest amplification of ischemia-induced responses in these patients may reflect increased electrical vulnerability, which could have implications for risk stratification and closer monitoring during acute ischemic episodes.

• In the Limitations and future work section, the text describing future work was revised to include potential clinical applications. Specifically, the following paragraph was added: Beyond these methodological considerations, the present findings also point toward several potential avenues for clinical translation. The use of normalized indices such as Rι, which account for baseline variability, may enable more individualized ECG interpretation compared to conventional fixed thresholds. In addition, the integration of multiple ECG markers capturing both depolarization and repolarization processes could enhance diagnostic performance, particularly in early or ambiguous presentations of acute coronary syndromes. More broadly, combining ECG-derived features with clinical variables such as age, sex, and prior MI may support the development of predictive models for improved decision-making. Further prospective studies are warranted to assess the diagnostic utility and incremental value of these approaches in real-world settings.

- The authors should consider multivariable regression analysis to determine whether prior MI remains an independent predictor of ECG changes after adjusting for potential confounders (e.g., ischemia extent/severity, age, sex, occlusion site, angiographic data).

Following the Reviewer’s comment, we performed univariable linear regression analyses to quantify the contribution of prior MI to ischemia-induced ECG changes. In these models, the relative change in each ECG marker measured 150 seconds after occlusion onset was used as the dependent variable, with prior MI as the sole independent variable. The proportion of explained variance was minimal, with R2 = 0.002 for STdev, R2 = 0.033 for Tam, and R2 = 0.005 for QRSw.

For comparison, we evaluated additional univariable models including other covariates (age, sex, occluded artery location, and ischemic extent and severity). Among these, only ischemic extent and severity ex-plained a substantial fraction of the variance in ECG changes. At 150 seconds after occlusion, the R2 values for ischemic extent/severity were 0.337/0.512 for STdev, 0.141/0.174 for Tam, and 0.255/0.340 for QRSw.

We further performed multivariable regression analyses including prior MI together with ischemic extent (or severity). The corresponding R2 values were 0.339 (extent) and 0.513 (severity) for STdev, 0.175 and 0.215 for Tam, and 0.260 and 0.349 for QRSw, indicating only marginal improvement compared with univariable models based on ischemic characteristics alone.

Finally, we explored combinations of predictors and found that ischemic extent and severity together provided the most complementary information. A model including both variables yielded R2 values of 0.711 for STdev, 0.188 for Tam, and 0.395 for QRSw. Adding prior MI as an additional covariate in-creased the explained variance slightly, with a more marked impact for Tam, resulting in R2 values of 0.713, 0.236, and 0.412, respectively.

Taken together, these analyses indicate that, although prior MI explains only a small proportion of the variability in ischemia-induced ECG changes compared with scintigraphic measures, its presence appears to modestly reinforce the strength of the association between ECG changes and ischemic extent or severity.

The above results were incorporate into the manuscript and appear as the final paragraph of subsection Relationship Between ECG Changes, History of Prior MI and Ischemia Extent / Severity within the Results section. The added paragraph reads as follows:

To further quantify the relative contribution of prior MI to ischemia-induced ECG changes, we per-formed additional univariable and multivariable regression analyses. When considering prior MI as the sole independent variable, the proportion of explained variance for each ECG marker at 150 s of occlusion was negligible, with R2 = 0.002, 0.033, and 0.005 for STdev, Tam, and QRSw, respectively. In contrast, ischemia extent and severity accounted for a substantially larger fraction of the variability, with R2 values of 0.337/0.512, 0.141/0.174, and 0.255/0.340, respectively. Including prior MI together with ischemic extent or severity in multivariable models resulted in only marginal improvements in explained variance. Notably, the combination of ischemia extent and severity yielded the highest explanatory power (R2 = 0.711, 0.188, and 0.395), while adding prior MI as an additional covariate produced only a slight increase, most evident for Tam where R2 = 0.236. These results indicate that, although prior MI has a limited direct contribution to the overall variability in ECG changes, its presence appears to modestly enhance the strength of the relationship between ECG changes and ischemic extent or severity.

References

[1] Rhee TM, Lee JM, Shin ES, Hwang D, Park J, Jeon KH, et al. Impact of optimized procedure-related factors in drug-eluting balloon angioplasty for treatment of in-stent restenosis. JACC: Cardiovascular Interventions. 2018;11(10):969-78. doi:10.1016/j.jcin.2018.02.002.

[2] Gurgoglione FL, Gattuso D, Greco A, Donelli D, Niccoli G, Cortese B. Angiographic and clinical impact of balloon inflation time in percutaneous coronary interventions with sirolimus-coated balloon: A subanalysis of the EASTBOURNE study. Cardiovascular Revascularization Medicine. 2025;73:70-5. doi:10.1016/j.carrev.2024.07.021.

[3] Martínez JP, Almeida R, Olmos S, Rocha AP, Laguna P. A wavelet-based ECG delineator: eval-uation on standard databases. IEEE Transactions on Biomedical Engineering. 2004;51(4):570-81. doi:10.1109/TBME.2003.821031.

[4] Meyer C, Keiser H. Electrocardiogram baseline noise estimation and removal using cubic splines and state-space computation techniques. Computers and Biomedical Research. 1977;10(5):459-70. doi:10.1016/0010-4809(77)90021-0.

[5] Pueyo E, S¨ornmo L, Laguna P. QRS slopes for detection and characterization of myocardial ischemia. IEEE Transactions on Biomedical Engineering. 2008;55(2):468-77. doi:10.1109/TBME.2007.902228.

[6] Pahlm O, S¨ornmo L. Data processing of exercise ECG’s. IEEE Transactions on Biomedical Engi-neering. 1987;BME-34(2):158-65. doi:10.1109/TBME.1987.326040.

[7] Martínez JP, Pahlm O, Ringborn M, Warren S, Laguna P, S¨ornmo L. The STAFF III database: ECGs recorded during acutely induced myocardial ischemia. In: 2017 Computing in Cardiology (CinC). vol. 44. IEEE; 2017. p. 1-4. doi:10.22489/CinC.2017.266-133.

[8] Minchol´e A, Jager F, Laguna P. Discrimination between ischemic and artifactual ST seg-ment events in Holter recordings. Biomedical Signal Processing and Control. 2010;5(1):21-31. doi:10.1016/j.bspc.2009.09.001.

[9] The MathWorks Inc . MATLAB version: 9.13.0 (R2022b). Natick, Massachusetts, United States: The MathWorks Inc.; 2022. Available from: https://www.mathworks.com.

Attachment Submitted filename: Response_to_Reviewers.pdf

# Peer review: Decision Letter 1

**DOI:** 10.1371/journal.pone.0361220.r003

**Authors:** Redoy Ranjan

18 Aug 2026

Dear Dr. Rodríguez-Carbó,

Thank you for submitting your manuscript to PLOS One. After careful consideration, we feel that it has merit but does not fully meet PLOS One’s publication criteria as it currently stands. Therefore, we invite you to submit a revised version of the manuscript that addresses the points raised during the review process.

Please submit your revised manuscript by Oct 02 2026 11:59PM. If you will need more time than this to complete your revisions, please reply to this message or contact the journal office at plosone@plos.org. When you're ready to submit your revision, log on to https://www.editorialmanager.com/pone/ and select the 'Submissions Needing Revision' folder to locate your manuscript file.

- A letter that responds to each point raised by the academic editor and reviewer(s). You should upload this letter as a separate file labeled 'Response to Reviewers'.
- A marked-up copy of your manuscript that highlights changes made to the original version. You should upload this as a separate file labeled 'Revised Manuscript with Track Changes'.
- An unmarked version of your revised paper without tracked changes. You should upload this as a separate file labeled 'Manuscript'.

If you would like to make changes to your financial disclosure, please include your updated statement in your cover letter. Guidelines for resubmitting your figure files are available below the reviewer comments at the end of this letter.

If applicable, we recommend that you deposit your laboratory protocols in protocols.io to enhance the reproducibility of your results. Protocols.io assigns your protocol its own identifier (DOI) so that it can be cited independently in the future. For instructions see: https://journals.plos.org/plosone/s/submission-guidelines#loc-laboratory-protocols. Additionally, PLOS One offers an option for publishing peer-reviewed Lab Protocol articles, which describe protocols hosted on protocols.io. Read more information on sharing protocols at https://plos.org/protocols?utm_medium=editorial-email&utm_source=authorletters&utm_campaign=protocols.

As the corresponding author, your ORCID iD is verified in the submission system and will appear in the published article. PLOS supports the use of ORCID, and we encourage all coauthors to register for an ORCID iD and use it as well. Please encourage your coauthors to verify their ORCID iD within the submission system before final acceptance, as unverified ORCID iDs will not appear in the published article. *Only*  the individual author can complete the verification step; PLOS staff *cannot*  verify ORCID iDs on behalf of authors.

We look forward to receiving your revised manuscript.

Kind regards,

Redoy Ranjan, MS (CV&TS), Ch.M. (Edin), PhD

Academic Editor

PLOS One

Journal Requirements:

If the reviewer comments include a recommendation to cite specific previously published works, please review and evaluate these publications to determine whether they are relevant and should be cited. There is no requirement to cite these works unless the editor has indicated otherwise.

Please review your reference list to ensure that it is complete and correct. If you have cited papers that have been retracted, please include the rationale for doing so in the manuscript text, or remove these references and replace them with relevant current references. Any changes to the reference list should be mentioned in the rebuttal letter that accompanies your revised manuscript. If you need to cite a retracted article, indicate the article’s retracted status in the References list and also include a citation and full reference for the retraction notice.

[Note: HTML markup is below. Please do not edit.]

Reviewers' comments:

Reviewer's Responses to Questions

**Comments to the Author**

Reviewer #3: (No Response)

Reviewer #4: All comments have been addressed

**********

2. Is the manuscript technically sound, and do the data support the conclusions?

Reviewer #3: No

Reviewer #4: Yes

**********

3. Has the statistical analysis been performed appropriately and rigorously?

Reviewer #3: No

Reviewer #4: Yes

**********

4. Have the authors made all data underlying the findings in their manuscript fully available?

The PLOS Data policy

Reviewer #3: No

Reviewer #4: Yes

**********

5. Is the manuscript presented in an intelligible fashion and written in standard English?

Reviewer #3: Yes

Reviewer #4: Yes

**********

Reviewer #3: Perform multivariable analyses to identify independent predictors of ECG changes.

Address multiple-comparison testing.

Clarify how prior MI was diagnosed.

Provide more detailed statistical methods and effect sizes.

Reduce overstatement regarding clinical applicability.

Include additional numerical summary tables.

Expand the limitations to address generalizability, small subgroup sizes, and absence of external validation.

Clarify whether medications or procedural variables could have influenced ECG responses.

Reviewer #4: The authors have satisfactorily addressed all comments raised by both reviewers in the previous round. In particular:

The addition of Table 1 (baseline characteristics) and Figure 8 (representative ECG examples) substantially improves clarity and reproducibility.

The revised Materials and Methods section now provides sufficiently detailed and reproducible descriptions of ECG preprocessing and marker definitions.

The newly performed univariable and multivariable regression analyses appropriately and transparently characterize the limited independent contribution of prior MI to ischemia-induced ECG changes, and the authors have moderated their interpretation accordingly.

Statistical reporting (p-values, 95% CIs, correlation coefficients) has been incorporated throughout the Results section as requested.

The Discussion section now adequately addresses clinical implications and limitations, including the reliance on baseline ECG recordings and the constraints of sample size.

**********

what does this mean?). If published, this will include your full peer review and any attached files.

If you choose “no”, your identity will remain anonymous but your review may still be made public.

**Do you want your identity to be public for this peer review?**  For information about this choice, including consent withdrawal, please see our Privacy Policy

Reviewer #3: **Yes:**  Isha Abdullah Ali

Reviewer #4: No

**********

[NOTE: If reviewer comments were submitted as an attachment file, they will be attached to this email and accessible via the submission site. Please log into your account, locate the manuscript record, and check for the action link "View Attachments". If this link does not appear, there are no attachment files.]

To ensure your figures meet our technical requirements, please review our figure guidelines: https://journals.plos.org/plosone/s/figures

You may also use PLOS’s free figure tool, NAAS, to help you prepare publication quality figures: https://journals.plos.org/plosone/s/figures#loc-tools-for-figure-preparation.

NAAS will assess whether your figures meet our technical requirements by comparing each figure against our figure specifications.

# Author comment: Author response to Decision Letter 2

**DOI:** 10.1371/journal.pone.0361220.r004

28 Sep 2026

Editor in Chief

PLOS One

Dear Editor,

We would like to thank you for the opportunity to revise our manuscript, “Characterizing acute ischemia via ECG: a study of severity, extent, and spatial variability in myocardial infarction and non-infarction groups”. We appreciate the review and the constructive suggestions provided by the Reviewers. We believe the manuscript has been strengthened by incorporating these modifications.

Journal Requirements:

If the reviewer comments include a recommendation to cite specific previously published works, please review and evaluate these publications to determine whether they are relevant and should be cited. There is no requirement to cite these works unless the editor has indicated otherwise.

Please review your reference list to ensure that it is complete and correct. If you have cited papers that have been retracted, please include the rationale for doing so in the manuscript text, or remove these references and replace them with relevant current references. Any changes to the reference list should be mentioned in the rebuttal letter that accompanies your revised manuscript. If you need to cite a retracted article, indicate the article’s retracted status in the References list and also include a citation and full reference for the retraction notice.

Reviewers' comments:

Reviewer's Responses to Questions

Comments to the Author

1. If the authors have adequately addressed your comments raised in a previous round of review and you feel that this manuscript is now acceptable for publication, you may indicate that here to bypass the “Comments to the Author” section, enter your conflict of interest statement in the “Confidential to Editor” section, and submit your "Accept" recommendation.

Reviewer #3: (No Response)

Reviewer #4: All comments have been addressed

2. Is the manuscript technically sound, and do the data support the conclusions?

The manuscript must describe a technically sound piece of scientific research with data that supports the conclusions. Experiments must have been conducted rigorously, with appropriate controls, replication, and sample sizes. The conclusions must be drawn appropriately based on the data presented.

Reviewer #3: No

Reviewer #4: Yes

3. Has the statistical analysis been performed appropriately and rigorously?

Reviewer #3: No

Reviewer #4: Yes

4. Have the authors made all data underlying the findings in their manuscript fully available?

The PLOS Data policy requires authors to make all data underlying the findings described in their manuscript fully available without restriction, with rare exception (please refer to the Data Availability Statement in the manuscript PDF file). The data should be provided as part of the manuscript or its supporting information, or deposited to a public repository. For example, in addition to summary statistics, the data points behind means, medians and variance measures should be available. If there are restrictions on publicly sharing data—e.g. participant privacy or use of data from a third party—those must be specified.

Reviewer #3: No

Reviewer #4: Yes

5. Is the manuscript presented in an intelligible fashion and written in standard English?

PLOS ONE does not copyedit accepted manuscripts, so the language in submitted articles must be clear, correct, and unambiguous. Any typographical or grammatical errors should be corrected at revision, so please note any specific errors here.

Reviewer #3: Yes

Reviewer #4: Yes

6. Review Comments to the Author

Please use the space provided to explain your answers to the questions above. You may also include additional comments for the author, including concerns about dual publication, research ethics, or publication ethics. (Please upload your review as an attachment if it exceeds 20,000 characters)

Reviewer #3:

We are grateful to the Reviewer for the revision and for the helpful and constructive comments, which we addressed as follows.

--- Perform multivariable analyses to identify independent predictors of ECG changes.

Following the Reviewer's comment, we performed multivariable linear regression analysis to quantify the independent contribution of sex, age, prior MI, and ischemia extent and severity to ischemia-induced ECG changes.

The updated paragraph in the Results section, subsection \textit{Relationship Between ECG Changes, History of Prior MI and Ischemia Extent / Severity}, that presents the results of this multivariable analysis reads as follows:

Additional statistical analyses were performed to quantify the relative contribution of prior MI, age, sex, and ischemia extent and severity to ischemia-induced ECG changes using univariable and multivariable regression models. When considering prior MI as the sole independent variable, the proportion of explained variance for each ECG marker at 150 s of occlusion was negligible, with R2 = 0.003, 0.034, and 0.005 for STdev , Tam, and QRSw, respectively. In contrast, ischemia extent and severity accounted

for a substantially larger fraction of the variability, with R2 values of 0.337/0.512, 0.141/0.175, and 0.255/0.340, respectively. Including prior MI together with ischemic extent or severity in multivariable models resulted in marginal improvements in explained variance, with more accentuated effects for Tam. Notably, the combination of ischemia extent and severity yielded higher explanatory power (R2 = 0.711, 0.188, and 0.395), while adding prior MI as an additional covariate produced a slight increase, most evident for Tam, with R2 = 0.236. These results indicate that, although prior MI has a limited direct contribution to the overall variability in ECG changes, its presence appears to modestly enhance the strength of the relationship between ECG changes and ischemic extent and severity. Including sex, age, prior MI, and ischemia extent and severity as independent variables in a multivariable model increased R2 values to 0.727, 0.303, and 0.466 for STdev , Tam, and QRSw, respectively. In this fully adjusted model, ischemia extent and severity were independently associated with STdev , whereas ischemia severity was independently associated with QRSw. Age, sex, and prior MI were not independently associated with any of the evaluated ECG markers. For Tam, the overall multivariable model did not reach statistical significance

--- Address multiple-comparison testing.

We thank the Reviewer for this suggestion. To address the correction for multiple hypothesis testing, p-values were adjusted using the Benjamini–Hochberg method. Specifically, Figures 4, 5, 6, 9, and 10, as well as the corresponding supplementary figures, were updated to reflect the correction for multiple comparisons. After correction, significant comparisons remained in Figures 9 and 10, which are indicated directly in the corresponding panels. In addition, effect sizes were reported to complement the adjusted p-values and quantify the magnitude of the observed differences. The statistical methods were expanded to describe this correction as follows:

To characterize the overall patient-level response of each ECG marker ι, the 90th percentile of Ri across the 12 leads was computed for each patient at each time point tj . These values were then summarized by reporting the median across patients. To account for multiple hypothesis testing, p-values were adjusted using the Benjamini–Hochberg procedure, applied within each family of related comparisons. Because statistical significance is influenced by sample size, effect sizes were additionally reported to quantify the magnitude of the observed differences. Specifically, the effect size r was reported for the Wilcoxon rank-sum test, whereas η2H was reported for the Kruskal–Wallis test [1]. Following the guidelines in [1], the Wilcoxon rank-sum effect size r ranges from −1 to 1 and was interpreted based on its absolute value as small (0.1 ≤ |r| < 0.3), medium (0.3 ≤ |r| < 0.5), or large (|r| ≥ 0.5). Similarly, the Kruskal–Wallis effect size η2H ranges from 0 to 1 and was classified as small (0.01 ≤ η2H < 0.06), medium (0.06 ≤ η2H < 0.14), or large (η2H ≥ 0.14), with larger values indicating greater effect magnitude [1].

--- Clarify how prior MI was diagnosed.

We acknowledge the Reviewer’s comment. We have clarified how prior MI was defined in the Materials

and methods section under the Study Population subsection:

Prior MI was identified using ECG-based screening criteria applied to a standard ECG recorded at hospital admission [2]. These screening criteria were based on a subset of the Selvester QRS scoring system [3], which assigns weighted points according to the duration and amplitude of individual QRS deflections across the ECG leads. The screening criteria were: (1) a Q-wave duration ≥ 30 ms in lead aVF; (2) an R-wave duration ≤ 10 ms and R-wave amplitude ≤ 0.1 mV in lead V2; and (3) an R-wave duration

≥ 40 ms in lead V1. The remaining criteria of the full 32-point Selvester QRS scoring system [4] were considered non-screening criteria. Patients were classified as having no prior MI if none of the three screening criteria was present in any available cardiac cycle and the total score from the non-screening criteria was ≤ 2 points. Prior MI was defined by the presence of at least one screening criterion in all available cardiac cycles or by a total score ≥ 4 points from the non-screening criteria, with at least two of these points derived from leads other than V4–V6. Patients who met neither the no-MI nor the MI criteria were classified as borderline for previous MI and excluded from further analysis [5].

--- Provide more detailed statistical methods and effect sizes.

Following the Reviewer’s comment, we have expanded the Materials and methods section, particularly the subsection describing the statistical methods, and added effect-size estimates to the relevant group-comparison figures (Figures 9 and 10 and Figures S5, S6, S7, and S8 in the Supplementary Material).The updated text reads as follows:

To characterize the overall patient-level response of each ECG marker ι, the 90th percentile of Rι across the 12 leads was computed for each patient at each time point tj . These values were then summarized by reporting the median across patients. To account for multiple hypothesis testing, p-values were adjusted using the Benjamini–Hochberg procedure, applied within each family of related comparisons. Because statistical significance is influenced by sample size, effect sizes were additionally reported to quantify the magnitude of the observed differences. Specifically, the effect size r was reported for the Wilcoxon rank-sum test, whereas η2H was reported for the Kruskal–Wallis test [1]. Following the guidelines in [1], the Wilcoxon rank-sum effect size r ranges from −1 to 1 and was interpreted based on its absolute value as small (0.1 ≤ |r| < 0.3), medium (0.3 ≤ |r| < 0.5), or large (|r| ≥ 0.5). Similarly, the Kruskal–Wallis effect size η2H ranges from 0 to 1 and was classified as small (0.01 ≤ η2H < 0.06), medium (0.06 ≤ η2H < 0.14), or large (η2H ≥ 0.14), with larger values indicating greater effect magnitude [1].

Additionally, we have added the following phrases in the Results section, subsection Relationship Between ECG Changes and Site of Occlusion, to comment on the effect size of the group comparison and the pairwise comparisons:

o examine whether the site of coronary occlusion influences the magnitude and spatial distribution of ischemia-induced ECG alterations, Fig 9 presents selected ECG markers (STdev , Tam, and QRSw) measured 150 seconds after occlusion for patients with LAD, RCA, and LCx artery occlusions. The left panels show the box plots of Rι for each marker, computed as the 90th percentile across the 12 ECG leads at 150 s post-occlusion for each artery group. Repolarization markers (STdev and Tam) exhibited the highest median |Rι| values in patients with LAD occlusion, followed by RCA, with the lowest values observed in the LCx group. For example, in STdev , patients with LAD occlusion reached median values of 68-fold compared to baseline, 29-fold in patients with RCA occlusion, and 21-fold for patients with LCx occlusion. Statistically significant differences in |Rι| were observed across artery groups for STdev , with a medium overall effect size (η2H = 0.08). The significant LAD versus LCx comparison also showed a medium effect size (|r| = 0.40). Differences in depolarization markers were less pronounced, although QRSw showed slightly higher median values in the LCx group.

The right panels of Fig 9 illustrate the spatial distribution of |Rι| across the 12 leads for each artery group. Distinct lead-specific patterns emerged depending on the occluded artery. LAD occlusion produced prominent changes in leads V2–V5, consistent with anterior myocardial ischemia. RCA occlusion was associated with the largest changes in leads II, III, and aVF, whereas LCx occlusion did not yield a clearly distinctive spatial pattern.

Consistent with these ECG findings, patients with LAD occlusion exhibited the highest median ischemia extent and severity, followed by LCx and RCA occlusions (Fig 10). Significant differences were observed across artery groups for both ischemia extent and severity, with medium overall effect sizes (η2H = 0.09 for both variables). Pairwise comparisons involving LAD showed small effect sizes (|r| = 0.16–0.17). No significant age differences were observed among the artery groups, suggesting that

age was unlikely to account for these differences.

--- Reduce overstatement regarding clinical applicability.

We have modified the Discussion to address the Reviewer’s comment. In particular, the following subsections have been rephrased: Temporal Evolution of ECG Changes, Role of Sex and Age in Ischemic ECG Dynamics, Role of Occluded Artery in Acute Ischemic ECG Dynamics, and Role of Prior MI and Ischemic Extent and Severity in Acute Ischemic ECG Dynamics. The revised text addressing the potential clinical implications now reads as follows:

- In the subsection entitled Temporal Evolution of ECG Changes, the following paragraph has been rephrased:

From a clinical perspective, these findings suggest that repolarization markers such as STdev , STar , and T /QRS may serve as sensitive early indicators of acute myocardial ischemia, capturing electrical alterations within seconds of coronary occlusion. However, whether this rapid response translates into improved ischemia detection in clinical practice, particularly in emergency or pre-hospital settings, remains unknown. In contrast, the more gradual evolution of depolarization markers suggests a complementary role in tracking the progression of ischemia over time. Together, these observations indicate that combining early-responsive repolarization indices with more slowly evolving depolarization markers may provide complementary information for the detection and temporal characterization of ischemic events. Nevertheless, the clinical utility of such a combined approach remains to be studied in prospective clinical studies.

- In the subsection entitled Role of Sex and Age in Ischemic ECG Dynamics, within the text related to sex, the following paragraph has been rephrased:

These results suggest that fixed, absolute ECG thresholds may not fully capture sex-related differences in the magnitude and temporal evolution of ischemia-induced ECG changes. This observation is consistent with current guideline recommendations for sex-specific ST-elevation criteria and suggests that incorporating relative or dynamic ECG measures warrants further investigation as a means of improving diagnostic sensitivity. Validation in larger and more diverse cohorts is required to determine whether these differences have implications for the diagnosis and management of acute coronary syndromes in female patients.

- In the subsection entitled Role of Occluded Artery in Acute Ischemic ECG Dynamics, the following paragraph was modified:

In terms of their potential clinical relevance, our results indicate that quantitative ECG markers capture differences related to the anatomical location of coronary occlusion. The more pronounced and spatially localized changes observed in LAD occlusions suggest that the analyzed ECG indices may contain information relevant to identifying the affected coronary territory. Conversely, the less distinctive patterns associated with LCx occlusion are consistent with known limitations of the standard 12-lead ECG and support the potential value of additional posterior leads or complementary diagnostic tools when LCx involvement is suspected.

- In the subsection entitled Role of Prior MI and Ischemic Extent and Severity in Acute Ischemic ECG Dynamics, the following paragraph was modified:

Nevertheless, these observations suggest that relative changes in ECG markers may remain informative even in patients with pre-existing ECG abnormalities due to prior MI. This may be particularly relevant for characterizing ischemia-induced ECG changes in the presence of an altered baseline ECG. Moreover, the modest amplification of ischemia-induced responses observed in these patients may reflect increased electrical vulnerability, potentially providing additional insight into the electrophysiological consequences of prior MI. However, further validation in larger patient populations and in other clinical settings is needed before the clinical significance and potential utility of these findings can be established.

--- Include additional numerical summary tables.

Following the Reviewer’s suggestion, we have added a summary table in the Results section, specifically in the subsection Relationship Between ECG Changes, History of Prior MI and Ischemia Extent / Severity. Table 1 summarizes the patients’ maximum changes in STdev , Tam, and QRSw during the first four minutes of occlusion and quantified in both absolute |∆ι| and relative |Rι| terms. Numerical values show median (IQR) values separately for patients with and without prior MI.

Table 1: Median (IQR) values of the patients’ maximum relative |Rι| and absolute |∆ι| changes in STdev , Tam, and QRSw during the first four minutes of occlusion, stratified by prior MI status.

|

Rι| Non-prior MI (N = 68) Prior MI (N = 34)

STdev 39.7915 [19.47 , 73.67] 49.83 [15.20 , 98.92]

Tam 23.29 [15.85 , 42.71] 30.67 [17.31 , 53.56]

QRSw 29.63 [14.11 , 50.68] 31.11 [11.83 , 99.53]

|∆ι| Non-prior MI Prior MI

STdev [μV ] 155.79 [72.65 , 304.67] 168.19 [95.49 , 245.53]

Tam [μV ] 212.84 [145.83 , 299.52] 212.21 [131.15 , 361.85]

QRSw [ms] 31.54 [21.51 , 45.73] 38.94 [15.30 , 67.33]

--- Expand the limitations to address generalizability, small subgroup sizes, and absence of external validation. Clarify whether medications or procedural variables could have influenced ECG responses.

We acknowledge the Reviewer’s comments on the limitations of the study. We have expanded the Limitations and future work section to more explicitly address the generalizability of the findings, the limited size of some subgroups, the absence of external validation, and the potential influence of pharmacological and procedural factors. The new text reads:

Several limitations should be considered when interpreting the present findings:

• A limitation of this study is the reliance on a baseline ECG acquired prior to coronary occlusion, which may not always be available in routine clinical practice. Although alternative baseline recordings may be obtained from the earliest ECG recorded during patient transport or hospital admission, or from previous ECG examinations, such recordings may not fully represent a true pre-ischemic state. The present approach may therefore be most applicable in settings where serial ECG recordings are available, allowing assessment of intra-individual changes over time. While inter-individual variability limits direct comparison of marker values across patients, the relatively stable behavior of these markers within individuals suggests potential utility for longitudinal ischemia assessment. However, the feasibility and clinical value of this approach require further evaluation in real-world settings.

• The sample size is relatively small, reflecting the limited availability of this specific type of ECG recordings, which were collected in the early 2000s. Nevertheless, this database represents a unique human model of nearly five minutes of controlled ischemia induced during PCI. Given the small size of certain subcohorts, including patients with LAD artery occlusions (n = 34, 33.3%) and those with prior MI (n = 34, 33.3%), as well as the subgroup for whom information on ischemia extent and severity was available, the statistical findings, in particular those derived from subgroup and multivariate analyses, should be interpreted with appropriate caution.

• Occlusions occurred at varying anatomical levels (proximal, mid, or distal), and data on the extent of ischemia were unavailable for a substantial proportion of patients. These procedural differences may have influenced the magnitude and temporal evolution of the ECG response and could not be fully accounted for in the present analysis.

• Potential confounders, including left ventricular hypertrophy, left anterior fascicular block, long QT syndrome, other comorbidities, and pharmacological therapy, may have influenced the observed results. Because the investigated markers were evaluated relative to each patient’s own baseline ECG, these factors were present in both recordings, and their impact may therefore have been partially mitigated. Nevertheless, it remains possible that comorbidities and medication influenced the electrophysiological response to acute ischemia. Further studies are needed to assess the effects of these factors on the proposed ECG markers.

• Information regarding medical therapy in patients with a prior MI, as well as the timing of the previous infarction relative to the ischemic event, was not available, limiting a more detailed assessment of their potential influence on the ECG response.

• As noted above, the recordings used in this study originate from a highly specific human model of acute ischemia induced during PCI, which enabled controlled assessment of ischemia-related ECG changes. However, the controlled nature of this model may limit the generalizability of the findings to routine clinical practice. External validation in a comparable independent cohort is currently challenging, as datasets including similarly prolonged, controlled coronary occlusions during PCI are not readily available under current clinical practice. Further studies in contemporary real-world clinical settings and larger patient cohorts are therefore needed to confirm the robustness, generalizability, and applicability of these observations.

• Although recordings were acquired in the supine position, in which the standard 12-lead ECG may represent cardiac orientation differently than in the standing position, this factor is unlikely to account for the artery-specific spatial patterns observed in the present study.

• Finally, the documented location of the prior MI was less precise than that of the acute ischemic event, limiting detailed interpretation.

Beyond these methodological considerations, the present findings also point toward several potential avenues for clinical translation. The use of normalized indices such as Rι, which account for baseline variability, may facilitate the assessment of intra-individual ECG changes compared with conventional fixed thresholds. In addition, the integration of multiple ECG markers capturing both depolarization and repolarization processes may provide complementary information on the temporal evolution of acute

ischemia. More broadly, combining ECG-derived features with clinical variables such as age, sex, and prior MI may support the development of multivariable models for improved characterization of acute ischemia. Further prospective studies in larger patient cohorts and across diverse clinical settings are warranted to determine the diagnostic utility and incremental value of these approaches in real-world practice.

Reviewer #4:

We are grateful to the Reviewer for the revision and for the helpful and constructive comments.

The authors have satisfactorily addressed all comments raised by both reviewers in the previous round. In particular:

The addition of Table 1 (baseline characteristics) and Figure 8 (representative ECG examples) substantially improves clarity and reproducibility.

The revised Materials and Methods section now provides sufficiently detailed and reproducible descriptions of ECG preprocessing and marker definitions.

The newly performed univariable and multivariable regression analyses appropriately and transparently characterize the limited independent contribution of prior MI to ischemia-induced ECG changes, and the authors have moderated their interpretation accordingly.

Statistical reporting (p-values, 95% CIs, correlation coefficients) has been incorporated throughout the Results section as requested.

The Discussion section now adequately addresses clinical implications and limitations, including the reliance on baseline ECG recordings and the constraints of sample size.

7. PLOS authors have the option to publish the peer review history of their article (what does this mean?). If published, this will include your full peer review and any attached files.

If you choose “no”, your identity will remain anonymous but your review may still be made public.

Do you want your identity to be public for this peer review? For information about this choice, including consent withdrawal, please see our Privacy Policy.

Reviewer #3: Yes: Isha Abdullah Ali

Reviewer #4: No

References

[1] Fiel Peres F. Effect sizes for nonparametric tests. Biochemia Medica. 2026;36(1):5-16.

[2] Martínez JP, Pahlm O, Ringborn M, Warren S, Laguna P, S¨ornmo L. The STAFF III database: ECGs recorded during acutely induced myocardial ischemia. In: 2017 Computing in Cardiology (CinC). vol. 44. IEEE; 2017. p. 1-4. doi:10.22489/CinC.2017.266-133.

[3] Selvester RH, Wagner GS, Hindman NB. The Selvester QRS scoring system for estimating myocardial infarct size: the development and application of the system. Archives of Internal Medicine. 1985;145(10):1877-81.

[4] Anderson WD, Wagner NB, Lee KL, White RD, Yuschak J, Behar VS, et al. Evaluation of a QRS scoring system for estimating myocardial infarct size. VI: Identification of screening criteria for non-acute myocardial infarcts. The American Journal of Cardiology. 1988;61(10):729-33.

[5] Pettersson J, Carro E, Edenbrandt L, Maynard C, Pahlm O, Ringborn M, et al. Spatial, individual, and temporal variation of the high-frequency QRS amplitudes in the 12 standard electrocardiographic leads. American Heart Journal. 2000;139(2):352-8.

Attachment Submitted filename: Revisions.pdf

# Peer review: Decision Letter 2

**DOI:** 10.1371/journal.pone.0361220.r005

**Authors:** Redoy Ranjan

7 Oct 2026

<p>Characterizing acute ischemia via ECG: a study of severity, extent, and spatial variability in myocardial infarction and non-infarction groups

PONE-D-26-08831R2

Dear Dr. Rodríguez-Carbó,

We’re pleased to inform you that your manuscript has been judged scientifically suitable for publication and will be formally accepted for publication once it meets all outstanding technical requirements.

Within one week, you’ll receive an e-mail detailing the required amendments. When these have been addressed, you’ll receive a formal acceptance letter and your manuscript will be scheduled for publication.

An invoice will be generated when your article is formally accepted. Please note, if your institution has a publishing partnership with PLOS and your article meets the relevant criteria, all or part of your publication costs will be covered. Please make sure your user information is up-to-date by logging into Editorial Manager at Editorial Manager® and clicking the ‘Update My Information' link at the top of the page. For questions related to billing, please contact billing support.

If your institution or institutions have a press office, please notify them about your upcoming paper to help maximize its impact. If they’ll be preparing press materials, please inform our press team as soon as possible -- no later than 48 hours after receiving the formal acceptance. Your manuscript will remain under strict press embargo until 2 pm Eastern Time on the date of publication. For more information, please contact onepress@plos.org.

Kind regards,

Dr Redoy Ranjan, MS (CV&TS), Ch.M. (Edin), PhD

Academic Editor

PLOS One

Additional Editor Comments (optional):

Reviewers' comments:

Reviewer's Responses to Questions

**Comments to the Author**

Reviewer #3: All comments have been addressed

Reviewer #4: All comments have been addressed

**********

2. Is the manuscript technically sound, and do the data support the conclusions?

Reviewer #3: Yes

Reviewer #4: Yes

**********

3. Has the statistical analysis been performed appropriately and rigorously?

Reviewer #3: Yes

Reviewer #4: Yes

**********

4. Have the authors made all data underlying the findings in their manuscript fully available?

The PLOS Data policy

Reviewer #3: Yes

Reviewer #4: (No Response)

**********

5. Is the manuscript presented in an intelligible fashion and written in standard English?

Reviewer #3: Yes

Reviewer #4: Yes

**********

Reviewer #3: Thank you for addressing all the issues. I believe it would be a wonderful publication. And it should be published in Plos journal.

Reviewer #4: Thank you for the careful revision. The authors have adequately addressed my previous comments, including the additional multivariable analyses, correction for multiple comparisons, clarification of the definition of prior myocardial infarction, improved statistical reporting, and expanded discussion of study limitations. The interpretation has also been appropriately moderated.

I have no further major concerns and consider the manuscript suitable for publication in its current form.

**********

what does this mean?). If published, this will include your full peer review and any attached files.

If you choose “no”, your identity will remain anonymous but your review may still be made public.

**Do you want your identity to be public for this peer review?**  For information about this choice, including consent withdrawal, please see our Privacy Policy

Reviewer #3: **Yes:**  Dr. Isha Abdullah Ali

Reviewer #4: No

**********

# Editor report: Acceptance letter

**DOI:** 10.1371/journal.pone.0361220.r006

**Authors:** Redoy Ranjan

PONE-D-26-08831R2

PLOS One

Dear Dr. Rodríguez-Carbó,

I'm pleased to inform you that your manuscript has been deemed suitable for publication in PLOS One. Congratulations! Your manuscript is now being handed over to our production team.

At this stage, our production department will prepare your paper for publication. This includes ensuring the following:

* All references, tables, and figures are properly cited

* All relevant supporting information is included in the manuscript submission,

* There are no issues that prevent the paper from being properly typeset

You will receive further instructions from the production team, including instructions on how to review your proof when it is ready. Please keep in mind that we are working through a large volume of accepted articles, so please give us a few days to review your paper and let you know the next and final steps.

Lastly, if your institution or institutions have a press office, please let them know about your upcoming paper now to help maximize its impact. If they'll be preparing press materials, please inform our press team within the next 48 hours. Your manuscript will remain under strict press embargo until 2 pm Eastern Time on the date of publication. For more information, please contact onepress@plos.org.

You will receive an invoice from PLOS for your publication fee after your manuscript has reached the completed accept phase. If you receive an email requesting payment before acceptance or for any other service, this may be a phishing scheme. Learn how to identify phishing emails and protect your accounts at https://explore.plos.org/phishing.

If we can help with anything else, please email us at customercare@plos.org.

Thank you for submitting your work to PLOS One and supporting open access.

Kind regards,

PLOS One Editorial Office Staff

on behalf of

Dr. Redoy Ranjan

Academic Editor

PLOS One
